# *Z*-Selective semihydrogenation of alkynes via Ni/Lewis acid synergistic catalyzed system using DMF as hydrogen source and solvent

**DOI:** 10.3762/bjoc.22.79

**Published:** 2026-06-30

**Authors:** Lei Kang, Haifeng Gao, Luo Yang

**Affiliations:** 1 School of Surveying & Testing, Shaanxi Railway Institute, Weinan, 714000, China; 2 Key Laboratory for Green Organic Synthesis and Application of Hunan Province, College of Chemistry, Xiangtan University, Hunan, 411105, China

**Keywords:** alkynes, DMF, Ni/Lewis acid catalysis, *Z*-selective semihydrogenation

## Abstract

A Ni/Lewis acid dual-catalytic system has been developed for the *Z*-selective semihydrogenation of alkynes. Utilizing DMF as both the hydrogen donor and reaction medium, this method affords *Z*-alkenes in high yield with excellent stereoselectivity under mild conditions. The protocol employs cost-effective and readily available catalysts, and demonstrates broad applicability across a wide range of substrates.

## Introduction

*Z*-Olefins represent important structural units in natural products, pharmaceuticals, and functional materials, making the stereoselective semihydrogenation of alkynes a fundamental transformation in synthesis [1–5]. Among available methods, transition-metal catalysis has played a dominant role. The classical Lindlar catalyst remains the most widely applied heterogeneous system [6], yet it suffers from several limitations – including batch variability, alkene isomerization, and over-reduction to alkanes, which are particularly problematic for terminal and polar substrates [7–10]. Recent review articles have comprehensively summarized advances in this research field. For instance, Gregori et al. outlined the state-of-the-art progress on stereoselective semihydrogenation of alkynes catalyzed by first-row (3d) transition metals, which attract substantial attention owing to their natural abundance and low toxicity [5].

In recent years, homogeneous catalysts based on Ni, Cu, Rh, and Co have shown promise in addressing some of these issues [11–16]. Nickel, in particular, has emerged as an attractive candidate for transfer semihydrogenation [17–22]. Common hydrogen donors employed in transfer hydrogenation include isopropanol, formic acid, methanol, and water; these reagents are generally safer and easier to handle than high-pressure gaseous hydrogen [23]. Still, many of these systems depend on expensive ligands, toxic reductants, or relatively forcing conditions, which can limit their practical utility [24–27]. Single-metal catalytic approaches also frequently exhibit modest stereocontrol. While cobalt-based systems have demonstrated some ability to tune *Z*/*E* selectivity, they often rely on ammonia borane as the hydrogen donor, which presents certain atom-economic disadvantages [5,28–33]. Clearly, new strategies are needed to advance this field.

Synergistic catalysis, which combines two distinct catalytic components, has become a valuable approach for overcoming the limitations of single-catalyst systems [34–36]. Lewis acids are well known for their ability to activate polar functional groups and stabilize reactive intermediates, yet their combination with nickel catalysts has been little explored in alkyne semihydrogenation. We envisioned that merging nickel’s competence in alkyne activation and hydrogen transfer with a Lewis acid’s capacity to modulate reaction pathways could enhance both selectivity and efficiency.

Dimethylformamide (DMF), a commonly utilized polar aprotic solvent, has recently attracted interest as a hydrogen donor in catalytic reductions [37–40]. While DMF is classified as a substance of high concern (SVHC) due to its reproductive toxicity and potential health hazards upon improper handling [41], it offers practical advantages over traditional hydrogen sources such as H₂, including high stability, operational simplicity, and ease of handling under ambient conditions – features that align with the practical requirements of synthetic chemistry [24,42]. Its compatibility with both Lewis acids and nickel catalysts further suggests its potential as a combined solvent and hydrogen donor in a synergistic catalytic manifold [43–44]. Related amide derivatives, such as formamide, have also been employed as safe cyanide sources or hydrogen-donor precursors, offering useful precedents for their application in reduction chemistry [45–46].

In this work, we describe a new synergistic catalytic system for the *cis*-selective semihydrogenation of alkynes, utilizing a Lewis acid together with a nickel catalyst and DMF as the hydrogen source. This method achieves excellent *Z*-selectivity (up to 98:2 *Z*/*E* ratio), operates under mild conditions, and avoids noble metals, toxic reductants, and specialized pressure equipment. The synergy between the Lewis acid and the nickel catalyst, coupled with the dual role of DMF, enables efficient conversion of diverse internal and terminal alkynes to the corresponding *Z*-olefins. Beyond expanding the toolbox for stereocontrolled alkyne semihydrogenation, this study offers fresh perspectives on the design of Lewis acid–metal synergistic catalytic systems.

## Results and Discussion

We began by developing a base metal catalytic system for the semihydrogenation of diphenylacetylene (**1a**), motivated by the practical limitations associated with previous precious-metal Pd catalysts. Capitalizing on DMF's capacity to act as both solvent and hydrogen source, we investigated a Ni/Lewis acid co-catalytic approach. Employing Co(OAc)_2_·4H_2_O as the Lewis acid co-catalyst, we first evaluated the influence of the nickel precursor. As summarized in Table 1, the reaction of **1a** was performed in DMF (0.8 mL) at 150 °C for 24 h, using various Ni sources (20 mol %) and Co(OAc)_2_·4H_2_O (50 mol %).

**Table 1:** Evaluation of nickel precursors in the semihydrogenation of 1,2-diphenylacetylene (**1a**) catalyzed by Ni/Co(OAc)_2_·4H_2_O.

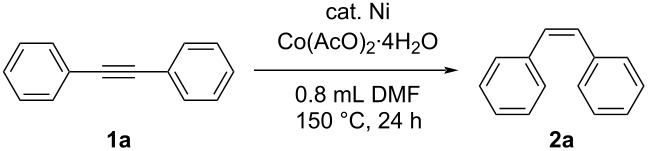			

Entry	[Ni] (20 mol %)	Lewis acid (50 mol %)	Yield [%]^a^

1	NiCl_2_	Co(AcO)_2_**·**4H_2_O	68
2	NiF_2_	Co(AcO)_2_**·**4H_2_O	23
3	Ni(acac)_2_	Co(AcO)_2_**·**4H_2_O	6
4	Ni(NO_3_)_2_**·**6H_2_O	Co(AcO)_2_**·**4H_2_O	34
5	Ni(Cp)_2_	Co(AcO)_2_**·**4H_2_O	30
6	(dppe)NiCl_2_	Co(AcO)_2_**·**4H_2_O	20
7	(Ph_3_P)_2_**·**NiCl_2_	Co(AcO)_2_**·**4H_2_O	43
8	(Ph_3_P)_2_**·**NiBr_2_	Co(AcO)_2_**·**4H_2_O	45
9	(dppp)NiCl_2_	Co(AcO)_2_**·**4H_2_O	50
10	NiCO_3_**·**2Ni(OH)_2_**·**4H_2_O	Co(AcO)_2_**·**4H_2_O	2
11	–	Co(AcO)_2_**·**4H_2_O	trace

^a^Yields were determined by GC analysis using mesitylene as an internal standard.

When NiCl₂ was used, **2a** (*cis*-stilbene) was obtained in 68% GC yield (Table 1, entry 1). In contrast, NiF_2_ and Ni(acac)_2_ proved considerably less effective, yielding only 23% and 6% of **2a**, respectively (Table 1, entries 2–3). Moderate conversions were observed with Ni(NO_3_)_2_·6H_2_O, NiCp_2_, (Ph_3_P)_2_NiCl_2_, (Ph_3_P)_2_NiBr_2_, and (dppp)NiCl_2_ (34%, 30%, 43%, 45% and 50% yields; Table 1, entries 4, 5, 7, 8, 9). Notably, phosphine-ligated Ni complexes such as (dppe)NiCl_2_ showed poor activity (20% yield, Table 1, entry 6), while NiCO_3_·2Ni(OH)_2_·4H_2_O afforded **2a** in 2% yield (entry 10). Unsurprisingly, control experiments confirmed that the reaction does not proceed in the absence of a nickel source (Table 1, entry 11). These findings underscore a strong dependence of catalytic activity on the nature of the Ni precursor, with NiCl_2_ emerging as the most effective candidate under the conditions screened.

Having identified NiCl_2_ as a suitable nickel source (Table 1), we next fixed this component (20 mol %) and proceeded to screen various Lewis acids (50 mol %) for the semihydrogenation of diphenylacetylene (**1a**). The reactions were conducted in DMF at 150 °C for 24 h, and the results are compiled in Table 2.

**Table 2:** Screening of Lewis acids for the NiCl_2_-co-catalyzed semihydrogenation of 1,2-diphenylacetylene (**1a**).

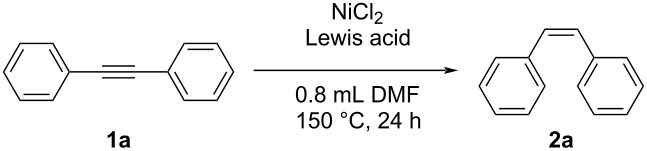				

Entry	[Ni] (20 mol %)	Lewis acid (50 mol %)	Temp (°C)	Yield [%]^a^

1	NiCl_2_	–	150	9
2	NiCl_2_	Co(AcO)_2_·4H_2_O	150	68
3	NiCl_2_	Fe(NO_3_)_3_·9H_2_O	150	trace
4	NiCl_2_	ZnCl_2_	150	96
5	NiCl_2_	CoCl_2_·6H_2_O	150	17
6	NiCl_2_	Co(acac)_2_	150	7
7	NiCl_2_	BF_3_·Et_2_O	150	trace
8	NiCl_2_	Zn(AcO)_2_·2H_2_O	150	98 (95; *Z*/*E* = 98:2)^b^
9	NiCl_2_	SnCl_2_·2H_2_O	150	11

^a^Yields were determined by GC analysis using mesitylene as an internal standard. Value in parentheses is the isolated yield. ^b^The *Z*/*E* ratio of the product was determined via GC of the reaction mixture.

Unsurprisingly, the Lewis acid proved crucial: in its absence, only a 9% GC yield of **2a** was obtained (Table 2, entry 1). Using Co(OAc)_2_·4H_2_O, our initial co-catalyst from the previous screening, gave a 68% yield (Table 1, entry 2), consistent with earlier data. Screening other Lewis acids, however, revealed considerable variation in performance. Fe(NO_3_)_3_·9H_2_O and BF_3_·Et_2_O, for instance, afforded merely trace product (Table 2, entries 3 and 7). Other candidates, including CoCl_2_·6H_2_O, Co(acac)_2_, and SnCl_2_·2H_2_O, showed low activity, yielding **2a** in 7–17% (Table 2, entries 5, 6, and 9).

Interestingly, zinc-based Lewis acids stood out. ZnCl_2_ significantly improved the yield to 96% (Table 2, entry 4). Most notably, Zn(OAc)_2_·2H_2_O delivered the best performance, achieving a 98% GC yield (95% isolated yield) of **2a** with an excellent *Z*/*E* selectivity of >98:2 (Table 2, entry 8). These results clearly demonstrate that zinc salts, particularly Zn(OAc)_2_·2H_2_O, form a highly effective synergistic pair with NiCl₂, dramatically enhancing both the efficiency and stereoselectivity of the transformation.

With Zn(OAc)_2_·2H_2_O established as the optimal Lewis acid (Table 2), we proceeded to optimize the remaining reaction parameters, keeping the Lewis acid loading fixed at 50 mol %. The outcomes of this study are summarized in Table 3. Consistent with our earlier findings, the nickel catalyst proved indispensable; omitting it resulted in only a trace of product **2a** (Table 3, entry 1). Screening various nickel precursors confirmed NiCl_2_ as the most effective, delivering **2a** in 98% GC yield (95% isolated) with excellent *Z*-selectivity (Table 3, entry 2). Other nickel sources, including Ni(acac)_2_ and phosphine-ligated complexes such as (dppe)NiCl_2_, gave notably lower yields (Table 3, entries 3–12). We then examined the catalyst loading. The combination of 20 mol % NiCl_2_ and 50 mol % Zn(OAc)_2_·2H_2_O (Table 3, entry 2) remained optimal. Reducing either the nickel loading (Table 3, entry 13) or the Lewis acid amount (Table 3, entries 14–15) led to a clear decrease in yield. While increasing both loadings slightly improved the yield (Table 3, entries 16–17), the marginal gain did not justify the higher catalyst use.

**Table 3:** Optimization of reaction conditions for the semihydrogenation of 1,2-diphenylacetylene (**1a**) catalyzed by NiCl_2_/Zn(OAc)_2_·2H_2_O.

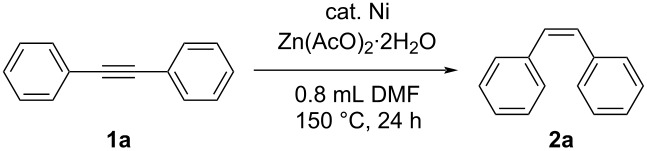				

Entry	[Ni] (20 mol %)	Lewis acid (50 mol %)	Temp (°C)	Yield [%]^a^

1	–	Zn(AcO)_2_**·**2H_2_O	150	trace
2	NiCl_2_	Zn(AcO)_2_**·**2H_2_O	150	98 (95)
3	NiF_2_	Zn(AcO)_2_**·**2H_2_O	150	73
4	Ni(acac)_2_	Zn(AcO)_2_**·**2H_2_O	150	45
5	Ni(NO_3_)_2_**·**6H_2_O	Zn(AcO)_2_**·**2H_2_O	150	4
6	Ni(AcO)_2_**·**4H_2_O	Zn(AcO)_2_**·**2H_2_O	150	89
7	(dppe)NiCl_2_	Zn(AcO)_2_**·**2H_2_O	150	73
8	(Ph_3_P)_2_**·**NiCl_2_	Zn(AcO)_2_**·**2H_2_O	150	69
9	(Ph_3_P)_2_**·**NiBr_2_	Zn(AcO)_2_**·**2H_2_O	150	50
10	NiCO_3_**·**2Ni(OH)_2_**·**4H_2_O	Zn(AcO)_2_**·**2H_2_O	150	79
11	(dppp)NiCl_2_	Zn(AcO)_2_**·**2H_2_O	150	72
12	NiSO_4_**·**6H_2_O	Zn(AcO)_2_**·**2H_2_O	150	78
13	NiCl_2_ (10 mol %)	Zn(AcO)_2_**·**2H_2_O (50 mol %)	150	65
14	NiCl_2_ (20 mol %)	Zn(AcO)_2_**·**2H_2_O (30 mol %)	150	54
15	NiCl_2_ (20 mol %)	Zn(AcO)_2_**·**2H_2_O (40 mol %)	150	85
16	NiCl_2_ (30 mol %)	Zn(AcO)_2_**·**2H_2_O (30 mol %)	150	86
17	NiCl_2_ (30 mol %)	Zn(AcO)_2_**·**2H_2_O (40 mol %)	150	92
18	NiCl_2_	Zn(AcO)_2_**·**2H_2_O	130	9
19	NiCl_2_	Zn(AcO)_2_**·**2H_2_O	140	88
20^b^	NiCl_2_	Zn(AcO)_2_**·**2H_2_O	150	46
21^c^	NiCl_2_	Zn(AcO)_2_**·**2H_2_O	150	85
22^d^	NiCl_2_	Zn(AcO)_2_**·**2H_2_O	150	13
23^e^	NiCl_2_	Zn(AcO)_2_**·**2H_2_O	150	60
24^f^	NiCl_2_	Zn(AcO)_2_**·**2H_2_O	150	trace
25^g^	NiCl_2_	Zn(AcO)_2_**·**2H_2_O	150	ND

^a^Yields were determined by GC analysis using mesitylene as an internal standard. Value in parentheses is the isolated yield. ^b^Reaction time was 12 h. ^c^Reaction time was 18 h. ^d^Formamide was used as the solvent/H-donor. ^e^*N*-Methylformamide was used as the solvent/H-donor. ^f^Anhydrous DMF was used as the reaction solvent. ^g^*N*,*N*-Dimethylacetamide (DMA) was used as a substitute for DMF as the solvent; not detected (ND).

The reaction temperature was also critical. A significant drop in yield was observed at 130 °C (Table 3, entry 18), and although 140 °C provided an 88% yield (entry 19), optimal conversion required 150 °C (entry 2). The reaction time was optimized to 24 hours; shorter (12 h, Table 3, entry 20) or moderately shorter (18 h, entry 21) durations resulted in incomplete conversion.

Finally, the role of DMF was confirmed. Replacing DMF with formamide afforded markedly reduced yields (Table 3, entry 22), primarily because severe competitive alkyne hydrocyanation consumes substrates, which was comprehensively explored in our previous work [47]. Similarly, poor yields were obtained when *N*-methylformamide was used as the alternative solvent (Table 3, entry 23). Notably, only trace target product was detected when rigorously dried anhydrous DMF (Table 3, entry 24) was applied, and no desired product could be isolated after switching the solvent to DMA (entry 25). These results further confirm the unique advantages of DMF acting as both solvent and hydrogen source.

After the above full-parameter condition optimization, the optimized conditions were established as: 20 mol % NiCl_2_, 50 mol % Zn(OAc)_2_·2H_2_O, in DMF at 150 °C for 24 h (Table 3, entry 2). This protocol afforded product **2a** in 98% GC yield (95% isolated) with >98:2 *Z*/*E* selectivity. Prolonging the reaction duration to 48 h did not cause obvious accumulation of the *E*-isomer product.

**Scheme 1:**
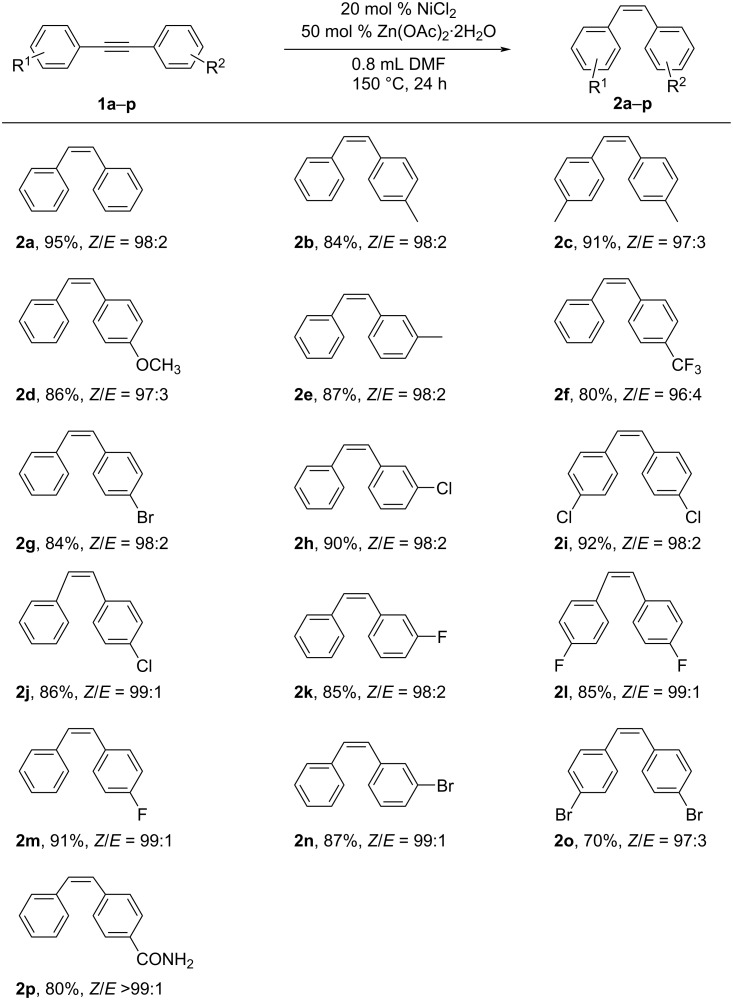
NiCl_2_/Zn(AcO)_2_·2H_2_O-co-catalyzed *Z*-selective semihydrogenation of internal alkynes to *Z*-internal alkenes.

Under the optimized conditions (20 mol % NiCl_2_, 50 mol % Zn(OAc)_2_·2H_2_O, DMF, 150 °C, 24 h), we evaluated the generality of this *Z*-selective semihydrogenation with various internal alkynes (Scheme 1). The system proved broadly effective for both symmetrical and unsymmetrical diarylacetylenes **1a**–**p**. Substrates bearing electron-donating groups (methyl **2b**, **2c**, **2e**; methoxy **2d**), electron-withdrawing groups (trifluoromethyl **2f**; halogens **2g**–**o**), and even an amide moiety (**2p**) were smoothly converted to the corresponding (*Z*)-stilbene derivatives in good to excellent yields (70–95%), with uniformly high stereoselectivity (*Z*/*E* = 97:3 to >99:1).

The reaction demonstrated excellent functional group tolerance. Ethers, halides (C–Cl, C–Br, C–F), and amides remained intact under the reaction conditions. Notably, substrates with strong electron-withdrawing substituents (e.g., –CF_3_ in **2f**) or multiple halogen atoms (**2i**, **2o**) were also well tolerated, affording the products in good yields and highlighting the robust applicability of this catalytic system.

Having optimized the conditions for internal alkynes such as diphenylacetylene, we next sought to apply this Ni/Zn dual-catalytic system to the semihydrogenation of terminal alkynes. Using phenylacetylene (**3a**) as the model substrate, we focused on screening the reaction temperature while maintaining a fixed reaction time of 24 h. The results are summarized in Table 4.

**Table 4:** Screening of reaction temperatures for the semihydrogenation of terminal alkynes.

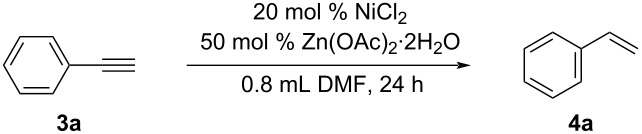		

Entry	Temp (°C)	Yield [%]^a^

1	130	30
2	135	58
3	140	70 (65)
4	145	67
5	150	51

^a^Yields were determined by GC using mesitylene as an internal standard. The value in parentheses is the isolated yield.

At 130 °C, the GC yield of styrene (**4a**) was only 30% (Table 4, entry 1). Raising the temperature to 135 °C improved the yield to 58% (Table 4, entry 2) and the optimal performance was observed at 140 °C, providing **4a** in 70% GC yield (64% isolated yield, entry 3). Further increases to 145 °C or 150 °C led to diminished yields of 67% and 51%, respectively (Table 4, entries 4–5). These results suggest that the reduction of terminal alkynes is more temperature-sensitive than that of internal alkynes, with an optimum at 140 °C compared to 150 °C for diphenylacetylene.

**Scheme 2:**
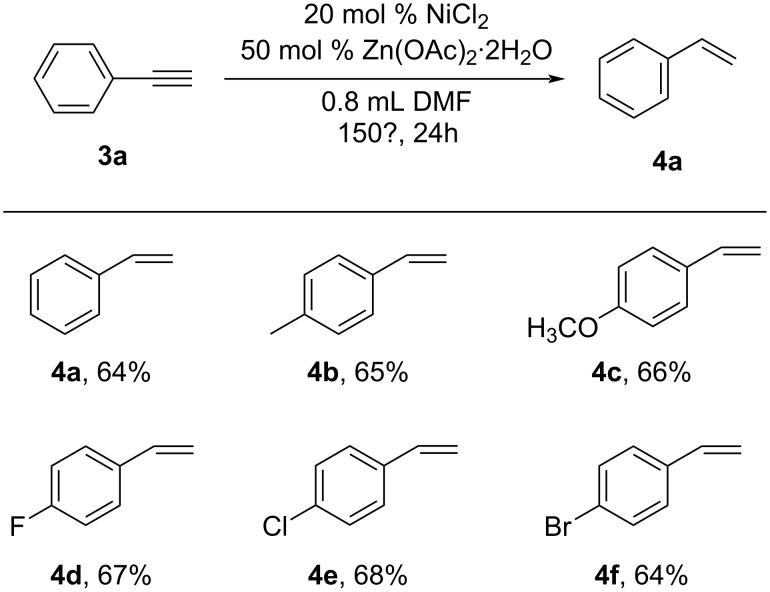
NiCl_2_/Zn(AcO)_2_·2H_2_O-co-catalyzed semihydrogenation of terminal alkynes to terminal alkenes.

With suitable conditions for the terminal alkyne **3a** in hand, we then examined the scope of this Ni/Zn system for various substituted terminal aryl alkynes (Scheme 2). Employing the standard protocol (20 mol % NiCl_2_, 50 mol % Zn(OAc)_2_·2H_2_O, DMF, 150 °C, 24 h), substrates bearing methyl (**4b**), methoxy (**4c**), and halogen (F for **4d**, Cl for **4e**, Br for **4f**) substituents were smoothly converted to the corresponding styrene derivatives in 64–68% yields. Both electron-donating groups (methyl, methoxy) and halogen functional groups (F, Cl, Br) remained intact under the reaction conditions without side reactions, fully demonstrating the good functional group tolerance and substrate generality of this catalytic system. We speculate that the relatively moderate isolated yields mainly originate from the thermal polymerization of the generated alkene products under the relatively high reaction temperature of 150 °C, which partially consumes the target olefin products and reduces the final isolated yields.

**Scheme 3:**
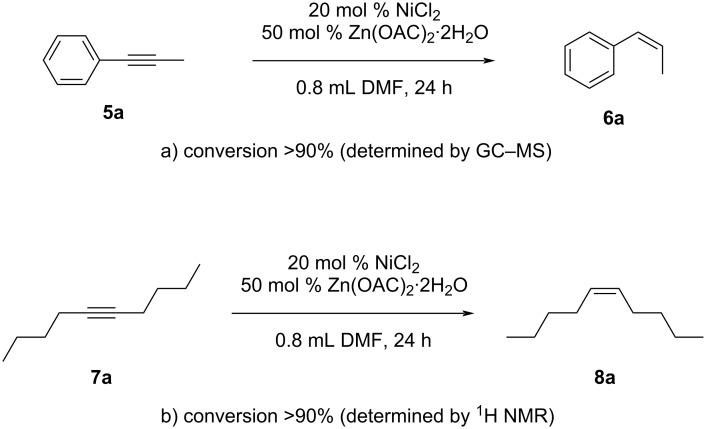
NiCl_2_/Zn(AcO)_2_·2H_2_O-co-catalyzed semihydrogenation of alkylarylacetylene and dialkylacetylene to (*Z*)-internal alkenes. See a) Figure S2 and b) Figure S3 in Supporting Information File 1.

In a preliminary experiment, we tested 1-phenyl-1-propyne (**5a**) and 5-decyne (**7a**) as substrates under the established conditions and both substrates were fully consumed. (Scheme 3) Compound **5a** was quantitatively converted to (*Z*)-1-phenyl-1-propene (**6a**), as confirmed by GC–MS, while **7a** delivered (*Z*)-5-decene (**8a**) within 24 h (based on the crude ¹H NMR spectrum; see Supporting Information File 1, Figure S3). No *E*-isomer was detected after 64 h, thus ruling out a catalyst-mediated *Z*/*E* isomerization.

**Scheme 4:**
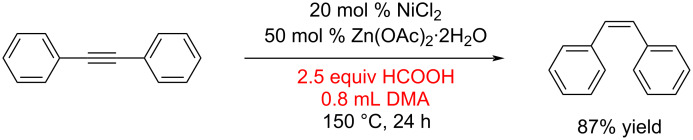
Control experiment using DMA as solvent together with exogenous formic acid instead of DMF.

To further verify that formic acid serves as the hydrogen donor while the polar amide solvent assists the catalytic cycle, diphenylacetylene was subjected to the standard reaction conditions using DMA together with 2.5 equivalents of HCOOH instead of DMF (Scheme 4). Gratifyingly, the desired (*Z*)-stilbene was obtained in 87% isolated yield, confirming that exogenous formic acid can be used as the hydrogen source for the alkyne semihydrogenation in our Ni/Zn synergistic catalytic system, and DMA can effectively replace DMF as the reaction medium to facilitate this transformation.

**Scheme 5:**
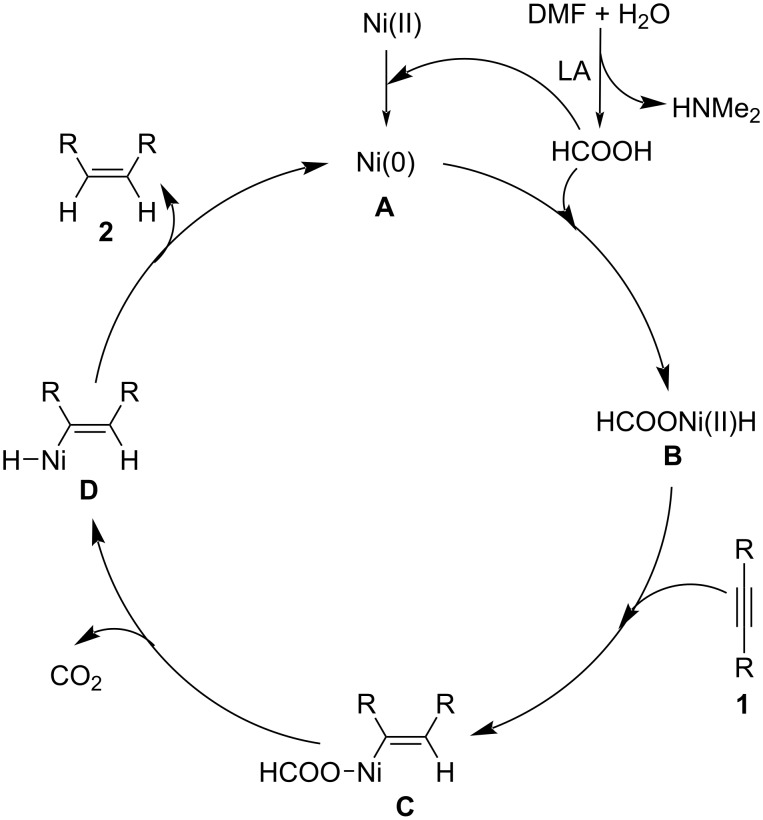
Proposed catalytic cycle for the Ni/Lewis acid-catalyzed semihydrogenation of alkynes with DMF.

Based on our experimental observations, well-established nickel chemistry [40,48], and relevant findings from palladium-catalyzed systems [49], we propose a plausible catalytic cycle for the NiCl_2_-catalyzed semihydrogenation of internal alkynes, as outlined in Scheme 5.

The cycle likely begins with the reduction of the Ni(II) precursor to an active Ni(0) species **A** – a common initiation step in nickel-catalyzed transformations, and the in-situ-generated formic acid functions as the reducing agent for this Ni(II)-to-Ni(0) conversion. Simultaneously, the Lewis acid promotes the hydrolysis of DMF with trace water existing in the solvent system to gradually liberate formic acid (HCOOH) and HNMe_2_. This in situ formation of HCOOH appears critical; analogous systems relying on DMF/water to generate HCOOH for the reduction of metal catalysts have been documented for both nickel and palladium catalysis in prior literature [11,40,49]. Maintaining an appropriate concentration of HCOOH via its gradual release from DMF hydrolysis thus seems essential for efficient semihydrogenation, a requirement that parallels findings in related transfer hydrogenation systems.

HCOOH then reacts with the Ni species to form a formate nickel hydride intermediate **B** (HCOONi(II)H), a key species documented in nickel-catalyzed hydrogen-transfer reactions. Subsequent selective insertion of the internal alkyne into the Ni–H bond of **B** affords the vinyl-nickel intermediate **C**. Following decarboxylation, the resulting dihydrido vinyl-nickel species **D** undergoes reductive elimination to release the (*Z*)-alkene product and regenerates the Ni(0) catalyst **A**, thereby closing the catalytic cycle.

## Conclusion

In summary, we have developed a highly *Z*-selective semihydrogenation of alkynes using a synergistic Ni/Lewis acid dual-catalytic system. The protocol employs DMF as a combined solvent and hydrogen donor, operating under mild conditions without external reductants or pressurized equipment. This method delivers *Z*-alkenes in high yields (up to 95%) with excellent stereoselectivity (up to >98:2 *Z*/*E* ratio) and demonstrates broad functional-group compatibility across a range of internal and terminal arylalkynes. Key practical advantages include the use of inexpensive, readily available nickel and zinc salts, as well as the operational simplicity afforded by DMF’s dual role. Overall, this work provides a practical and cost-effective alternative to existing semihydrogenation methods and highlights the potential of base metal synergistic catalysis in stereocontrolled synthetic transformations.

## Experimental

All experiments were performed under air. An oven-dried reaction vessel was charged with diphenylacetylene (0.2 mmol, 35.6 mg), Zn(OAc)_2_·2H_2_O (50 mol %, 22 mg), and NiCl_2_ (20 mol %, 3.3 mg). DMF (0.8 mL) was then added, the vessel was sealed, and the mixture was stirred and heated at 150 °C (oil bath temperature) for 24 h. The reaction was monitored by GC analysis; upon complete consumption of the starting material, heating was discontinued. After cooling to room temperature, the volatiles were removed under reduced pressure. The resulting crude residue was purified by flash column chromatography on silica gel (eluent: petroleum ether) to afford pure (*Z*)-stilbene (**2a**) as a colorless solid in 95% isolated yield (34.4 mg). The *Z*/*E* ratio (98:2) was determined by GC analysis of the crude mixture.

## Supporting Information

File 1 Experimental procedure, compound characterization data, and copies of spectra.

## Data Availability

All data that supports the findings of this study is available in the published article and/or the supporting information of this article.

## References

[1] Oger C, Balas L, Durand T, Galano J-M (2013). Chem Rev.

[2] Fürstner A, Guth O, Rumbo A, Seidel G (1999). J Am Chem Soc.

[3] Liang S, Hammond G B, Xu B (2016). Chem Commun.

[4] Kusy R, Grela K (2025). Chem Rev.

[5] Gregori B J, Schmotz M-O W S, Jacobi von Wangelin A (2022). ChemCatChem.

[6] Lindlar H (1952). Helv Chim Acta.

[7] Ulan J G, Maier W F, Smith D A (1987). J Org Chem.

[8] Kluwer A M, Koblenz T S, Jonischkeit T, Woelk K, Elsevier C J (2005). J Am Chem Soc.

[9] van Laren M W, Elsevier C J (1999). Angew Chem, Int Ed.

[10] Chen X-B, Zhang J R, Sun D-Q, Chen K-Q, Chen X-Y (2025). Synthesis.

[11] Richmond E, Moran J (2015). J Org Chem.

[12] Han X, Hu J, Chen C, Yuan Y, Shi Z (2019). Chem Commun.

[13] Fu S, Chen N-Y, Liu X, Shao Z, Luo S-P, Liu Q (2016). J Am Chem Soc.

[14] Rai R K, Awasthi M K, Singh V K, Barman S R, Behrens S, Singh S K (2020). Catal Sci Technol.

[15] Sheikh Mohammad T, Sakharov P, Raje S, de Ruiter G (2025). ACS Catal.

[16] Xia S, Peng J, Xie S, Xu T, Li L, Liu X, Cao D, He L-N, Li C-J (2025). Org Lett.

[17] Sansores-Paredes M L G, Lutz M, Moret M-E (2024). Nat Chem.

[18] Redl S, Topf C (2024). Tetrahedron Chem.

[19] Avello M G, Golling S, Truong-Phuoc L, Vidal L, Romero T, Papaefthimiou V, Gruber N, Chetcuti M J, Leroux F R, Donnard M (2023). Chem Commun.

[20] Li K, Yang C, Chen J, Pan C, Fan R, Zhou Y, Luo Y, Yang D, Fan B (2021). Asian J Org Chem.

[21] Thiel N O, Kaewmee B, Tran Ngoc T, Teichert J F (2020). Chem – Eur J.

[22] Wu Y, Ao Y, Li Z, Liu C, Zhao J, Gao W, Li X, Wang H, Liu Y, Liu Y (2023). Nat Commun.

[23] Wang D, Astruc D (2015). Chem Rev.

[24] Pape F, Thiel N O, Teichert J F (2015). Chem – Eur J.

[25] Wakamatsu T, Nagao K, Ohmiya H, Sawamura M (2016). Organometallics.

[26] Semba K, Kameyama R, Nakao Y (2015). Synlett.

[27] Chugh V, Wu J, Leutzsch M, Randel H, Weyhermüller T, Auer A A, Farès C, Werlé C (2024). Chem Catal.

[28] Qi X, Liu X, Qu L-B, Liu Q, Lan Y (2018). J Catal.

[29] Tokmic K, Fout A R (2016). J Am Chem Soc.

[30] Pandey D K, Khusnutdinova J R (2025). ChemCatChem.

[31] Ren X, Lu P, Zheng C, Wang Y, Lu Z (2025). Angew Chem, Int Ed.

[32] Li K, Khan R, Zhang X, Gao Y, Zhou Y, Tan H, Chen J, Fan B (2019). Chem Commun.

[33] Feng W-J, Chang Z, Lu X, Fu Y (2025). Nat Commun.

[34] Dong X-Q, Zhao Q, Li P, Chen C, Zhang X (2015). Org Chem Front.

[35] Deng Y, Kumar S, Wang H (2014). Chem Commun.

[36] Wasilke J-C, Obrey S J, Baker R T, Bazan G C (2005). Chem Rev.

[37] Zhao C, Wang Y, Pham Q, Dai C, Chatterjee A, Wasa M (2023). J Am Chem Soc.

[38] Lang Q, Gu G, Cheng Y, Yin Q, Zhang X (2018). ACS Catal.

[39] Wen J, Jiang J, Zhang X (2016). Org Lett.

[40] Guo S, Zhou J (Steve) (2016). Org Lett.

[41] Prat D, Pardigon O, Flemming H-W, Letestu S, Ducandas V, Isnard P, Guntrum E, Senac T, Ruisseau S, Cruciani P (2013). Org Process Res Dev.

[42] Gao Y, Yang R, Wamg C, Wu Y, Li H, Zhang B (2022). Sci Adv.

[43] Sarkar S, Jana M, Tadigoppula N (2013). RSC Adv.

[44] Cella R, Stefani H A (2006). Tetrahedron.

[45] Luo L, Dai H, Yang M-Q, Yang L (2024). Adv Synth Catal.

[46] Zhang J, Luo C-P, Yang L (2021). Adv Synth Catal.

[47] Yang L, Liu Y-T, Park Y, Park S-W, Chang S (2019). ACS Catal.

[48] Chen T, Xiao J, Zhou Y, Yin S, Han L-B (2014). J Organomet Chem.

[49] Li J, Hua R, Liu T (2010). J Org Chem.

