# Supplementary material for: Z-Selective semihydrogenation of alkynes via Ni/Lewis acid synergistic catalyzed system using DMF as hydrogen source and solvent
Source: Beilstein J Org Chem. 2026 Jun 30;22:1004–12. doi: 10.3762/bjoc.22.79 (PMC13338598; doi:10.3762/bjoc.22.79)
Supplement: File 1 — Experimental procedure, compound characterization data, and copies of spectra. [file Beilstein_J_Org_Chem-22-1004-s001.pdf]

BEILSTEIN JOURNAL OF ORGANIC CHEMISTRY

Supporting Information

for

Z-Selective semihydrogenation of alkynes via Ni/Lewis acid synergistic catalyzed system using DMF as hydrogen source and solvent

Lei Kang, Haifeng Gao and Luo Yang

Beilstein J. Org. Chem. **2026,** *22,* 1004–1012. doi:10.3762/bjoc.22.79

Experimental procedure, compound characterization data, and copies of spectra

License and Terms: This is a supporting information file under the terms of the Creative Commons Attribution License (https://creativecommons.org/licenses/by/4.0). Please note that the reuse, redistribution and reproduction in particular requires that the author(s) and source are credited and that individual graphics may be subject to special legal provisions.
The license is subject to the *Beilstein Journal of Organic Chemistry* terms and conditions: (https://www.beilstein-journals.org/bjoc/terms)

1. General information

Thin-layer chromatography (TLC) was performed using E. Merck Silica Gel 60 F254 precoated plates (0.25 mm). The developed chromatography plates were analyzed by a UV lamp (254 nm). Nuclear magnetic resonance (NMR) spectra were recorded on a Bruker Avance 400 spectrometer at ambient temperature. Chemical shifts for ^{1}H NMR spectra are reported in parts per million (ppm) from tetramethylsilane with the solvent resonance as the internal standard (chloroform: δ 7.26 ppm). Chemical shifts for ^{13}C NMR spectra are reported in parts per million (ppm) from tetramethylsilane with the solvent as the internal standard ($CDCl_3$: δ 77.16 ppm). Data are reported as follows: chemical shift, multiplicity (s = singlet, d = doublet, dd = doublet of doublets, t = triplet, q = quartet, m = multiplet, br = broad signal), coupling constant (Hz), and integration. The *Z*/*E* stereoisomeric ratio of the product was determined by gas chromatography (GC) analysis of the crude reaction mixture.

2. Experimental section

An oven-dried reaction vessel was charged with diphenylacetylene (0.2 mmol, 35.6 mg), $Zn(OAc)_2{\cdot}2H_2O$ (50 mol %, 22 mg), and $NiCl_2$ (20 mol %, 3.3 mg). DMF (0.8 mL) was then added, the vessel was sealed, and the mixture was stirred and heated at 150 °C (oil bath temperature) for 24 h. The reaction was monitored by GC analysis; upon complete consumption of the starting material, heating was discontinued. After cooling to room temperature, the volatiles were removed under reduced pressure. The resulting crude residue was purified by flash column chromatography on silica gel (eluent: petroleum ether) to afford pure (*Z*)-stilbene (**2a**) as a colorless solid in 95% isolated yield (34.4 mg). The *Z*/*E* ratio (98:2) was determined by GC analysis of the crude mixture.

3. Characterization data of products 2a–p

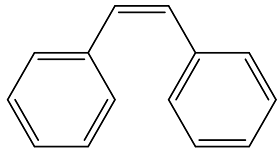

(2a)[1] **2a**

Yellow oil. ^{1}H NMR (400 MHz, $CDCl_3$, TMS) δ 7.24-7.17 (m, 10H), 6.60 (s, 2H);

^{13}C NMR (100 MHz, $CDCl_3$) δ 137.35, 130.37, 128.99, 128.33, 127.21.

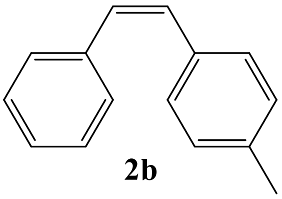

(2b)[2] **2b**

^{1}H NMR (400 MHz, $CDCl_3$, TMS) δ 7.30 – 7.17 (m, 5H), 7.17 – 7.10 (m, 2H), 7.02 (d, J = 7.9 Hz, 2H), 6.55 (s, 2H), 2.31 (s, 3H).

^{13}C NMR (100 MHz, $CDCl_3$) δ 137.53, 136.90, 134.29, 130.23, 129.58, 128.94, 128.88, 128.82, 128.22, 127.00, 21.27.

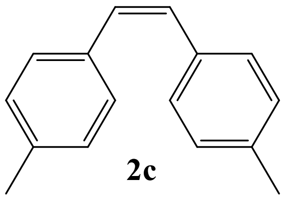

(2c)[1] **2c**

^{1}H NMR (400 MHz, $CDCl_3$, TMS) δ 7.16 (d, 4H, J = 8.2 Hz), 7.03 (d, 4H, J = 7.9 Hz), 6.51 (s, 2H), 2.31 (s, 6H);

^{13}C NMR (100 MHz, $CDCl_3$) δ 136.75, 134.51, 129.53, 128.90, 128.77, 21.26.

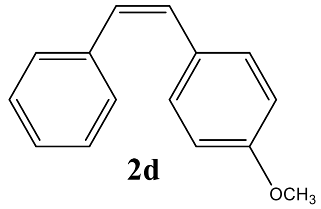

(2d)[1] **2d**

^{1}H NMR (400 MHz, $CDCl_3$, TMS) δ 7.50 – 6.91 (m, 7H), 6.75 (d, J = 8.8 Hz, 2H), 6.52 (d, J = 1.8 Hz, 2H), 3.78 (s, 3H).

^{13}C NMR (100 MHz, $CDCl_3$) δ 158.69, 137.64, 130.17, 129.79, 129.68, 128.84, 128.78, 128.25, 126.92, 113.61, 55.21.

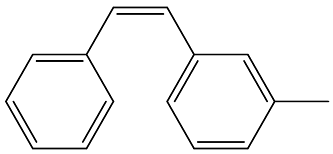

(2e)[2] **2e**

^{1}H NMR (400 MHz, $CDCl_3$) δ 7.34 – 7.17 (m, 5H), 7.09 (m, 4H), 6.60 (s, 2H), 2.29 (s, 3H).

^{13}C NMR (100 MHz, $CDCl_3$) δ 137.84, 137.41, 137.27, 130.45, 130.16, 129.69, 128.97, 128.24, 128.17, 127.94, 127.14, 125.96, 21.43.

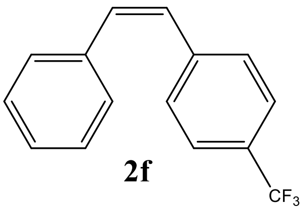

(2f)[2, 3] **2f**

^{1}H NMR (400 MHz, $CDCl_3$) δ 7.47 (d, J = 8.2 Hz, 2H), 7.34 (d, J = 8.4 Hz, 2H), 7.30 – 7.17 (m, 5H), 6.73 (d, J = 12.3 Hz, 1H), 6.60 (d, J = 12.3 Hz, 1H).

^{13}C NMR (100 MHz, $CDCl_3$) δ 140.92, 136.56, 132.34, 129.14, 128.82, 128.75, 128.43, 127.58, 125.16 (q, J_1 = 3.7, J_2 = 3.8 Hz), 122.83.

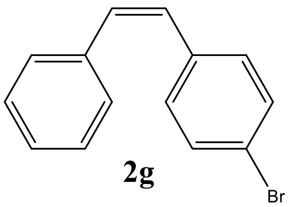

(2g)[4] **2g**

^{1}H NMR (400 MHz, $CDCl_3$) δ δ 7.34 (d, J = 8.4 Hz, 2H), 7.25 – 7.20 (m, 5H), 7.10 (d, J = 8.3 Hz, 2H), 6.63 (d, J = 12.2 Hz, 1H), 6.50 (d, J = 12.2 Hz, 1H).

^{13}C NMR (100 MHz, $CDCl_3$) δ 136.86, 136.14, 131.39, 131.07, 130.57, 128.97, 128.83, 128.38, 127.38, 120.96.

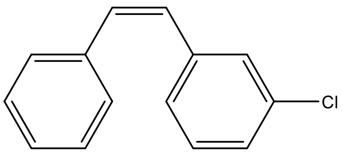

(2h)[2] **2h**

^{1}H NMR (400 MHz, $CDCl_3$) δ 7.35 – 7.03 (m, 9H), 6.68 (d, J = 12.2 Hz, 1H), 6.55 (d, J = 12.2 Hz, 1H).

^{13}C NMR (100 MHz, $CDCl_3$) δ 139.17, 136.68, 134.13, 131.64, 129.50, 128.91, 128.89, 128.78, 128.40, 127.52, 127.20, 127.07.

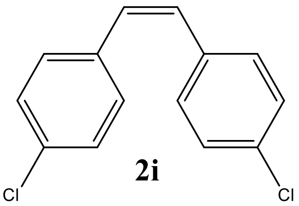

2i

(2i)[5]

^{1}H NMR (400 MHz, $CDCl_3$) δ 7.23 (d, J = 8.5 Hz, 4H), 7.17 (d, J = 8.4 Hz, 4H), 6.58 (s, 2H).

^{13}C NMR (100 MHz, $CDCl_3$) δ 135.29, 133.06, 130.17, 129.64, 128.58.

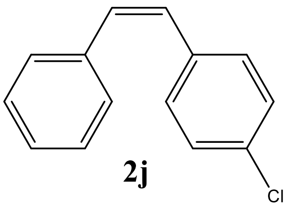

2j

(2j)[2]

^{1}H NMR (400 MHz, $CDCl_3$) δ 7.35 – 7.08 (m, 9H), 6.62 (d, J = 12.2 Hz, 1H), 6.53 (d, J = 12.2 Hz, 1H).

^{13}C NMR (100 MHz, $CDCl_3$) δ 136.90, 135.68, 132.77, 130.98, 130.25, 128.95, 128.84, 128.44, 128.37, 127.35.

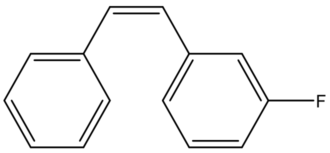

2k

(2k)[4]

^{1}H NMR (400 MHz, $CDCl_3$) δ 7.27 – 7.12 (m, 6H), 7.01 (d, *J* = 7.7 Hz, 1H), 6.97 – 6.83 (m, 2H), 6.65 (d, *J* = 12.2 Hz, 1H), 6.54 (d, *J* = 12.2 Hz, 1H).

^{13}C NMR (100 MHz, $CDCl_3$) δ 162.73 (d, *J* = 245.2 Hz), 139.51 (d, *J* = 7.8 Hz), 129.69 (d, *J* = 8.4 Hz), 129.00 (d, *J* = 2.2 Hz), 115.56 (d, *J* = 21.7 Hz), 114.01 (d, *J* = 21.2 Hz).

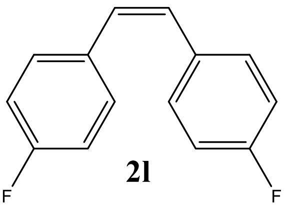

2l

(2l)[6]

^{1}H NMR (400 MHz, $CDCl_3$) δ 7.29 – 7.07 (m, 4H), 7.00 – 6.83 (m, 4H), 6.54 (s, 2H).

^{13}C NMR (100 MHz, $CDCl_3$) δ 161.87 (d, J = 246.9 Hz), 132.98 (d, J = 3.4 Hz), 130.50 (d, J = 7.9 Hz), 129.11 (d, J = 1.2 Hz), 115.28 (d, J = 21.4 Hz).

(2m)[4]

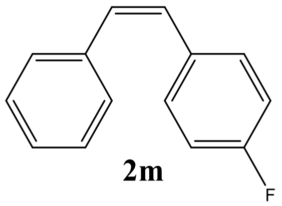

2m

^{1}H NMR (400 MHz, $CDCl_3$) δ 7.27 – 7.16 (m, 7H), 6.95 – 6.85 (m, 2H), 6.59 (d, J = 12.2 Hz, 1H), 6.53 (d, J = 12.2 Hz, 1H).

^{13}C NMR (100 MHz, $CDCl_3$) δ 161.85 (d, J = 246.7 Hz), 133.21 (d, J = 3.4 Hz), 130.56 (d, J = 7.8 Hz), 130.28 (d, J = 1.0 Hz), 115.18 (d, J = 21.4 Hz).

(2n)[7]

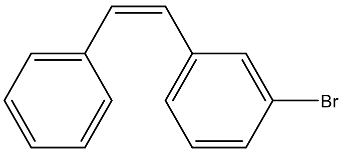

2n

^{1}H NMR (400 MHz, $CDCl_3$) δ 7.39 – 7.05 (m, 9H), 6.64 (d, *J* = 12.2 Hz, 1H), 6.51 (d, *J* = 12.2 Hz, 1H).

^{13}C NMR (100 MHz, $CDCl_3$) δ 139.45, 136.61, 131.79, 131.66, 130.08, 129.74, 128.86, 128.64, 128.36, 127.51, 127.47, 122.31.

(2o)[7]

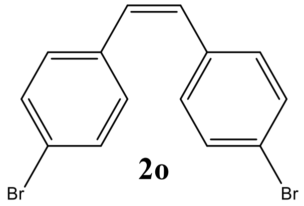

2o

^{1}H NMR (400 MHz, $CDCl_3$) δ 7.36 (d, *J* = 8.5 Hz, 4H), 7.08 (d, *J* = 8.5 Hz, 4H), 6.54 (s, 2H).

^{13}C NMR (100 MHz, $CDCl_3$) δ 135.70, 131.68, 131.54, 130.47, 129.73, 121.25.

(2p)

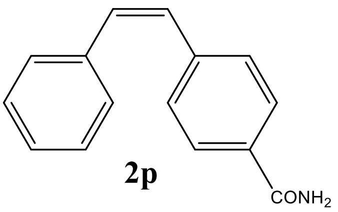

2p

^{1}H NMR (400 MHz, $CDCl_3$) δ 7.66 (d, *J* = 8.3 Hz, 2H), 7.30 (d, *J* = 8.1 Hz, 2H), 7.28 – 7.12 (m, 5H), 6.70 (d, *J* = 12.2 Hz, 1H), 6.59 (d, *J* = 12.2 Hz, 1H), 5.93 (d, *J* = 94.6 Hz, 2H).

^{13}C NMR (100 MHz, $CDCl_3$) δ 169.47, 141.20, 136.70, 132.10, 131.76, 129.10, 128.88, 128.39, 127.53, 127.38.

(4a)[5] 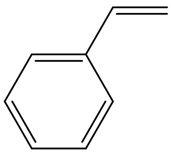 **4a**

^{1}H NMR (400 MHz, $CDCl_3$) δ 7.41 – 7.22 (m, 4H), 6.75 – 6.66 (m, 1H), 5.74 (d, J = 17.6 Hz, 1H), 5.23 (d, J = 11.0 Hz, 1H).

^{13}C NMR (100 MHz, $CDCl_3$) δ 137.70, 137.02, 128.66, 127.94, 126.35, 113.94.

(4b)[8] 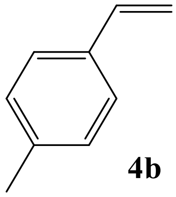 **4b**

^{1}H NMR (400 MHz, $CDCl_3$) δ 7.30 (d, J = 6.3 Hz, 2H), 7.12 (d, J = 6.6 Hz, 2H), 6.80 – 6.58 (m, 1H), 5.69 (d, J = 17.7 Hz, 1H), 5.18 (d, J = 10.9 Hz, 1H), 2.33 (s, 3H).

^{13}C NMR (100 MHz, $CDCl_3$) δ 137.74, 136.84, 134.95, 129.35, 126.25, 112.89, 21.34.

(4c)[5, 9] 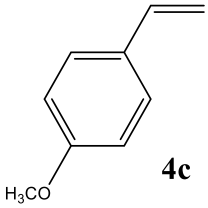

4c

^{1}H NMR (400 MHz, $CDCl_3$) δ 7.34 (d, J = 8.9 Hz, 2H), 6.85 (d, J = 8.9 Hz, 2H), 6.65 (dd, J = 17.7, 10.9 Hz, 1H), 5.60 (d, J = 17.7 Hz, 1H), 5.12 (d, J = 11.9 Hz, 1H), 3.79 (s, 3H).

^{13}C NMR (100 MHz, $CDCl_3$) δ 159.49, 136.34, 130.54, 127.50, 114.02, 111.68, 55.38.

(4d)[9] 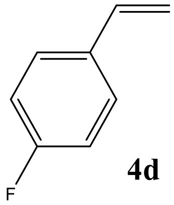

4d

^{1}H NMR (400 MHz, $CDCl_3$) δ 7.36 (dd, J = 8.9, 5.3 Hz, 2H), 7.05 – 6.94 (m, 2H), 6.67 (dd, J = 17.6, 10.9 Hz, 1H), 5.65 (d, J = 17.6 Hz, 1H), 5.21 (d, J = 10.9 Hz, 1H).

^{13}C NMR (100 MHz, $CDCl_3$) δ 162.58 (d, J = 246.6 Hz), 135.79, 133.84 (d, J = 3.4 Hz), 127.84 (d, J = 8.2 Hz), 115.51 (d, J = 21.7 Hz), 113.62 (d, J = 2.4 Hz).

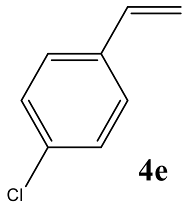

(4e)[5, 9] **4e**

^{1}H NMR (400 MHz, $CDCl_3$) δ 7.30 (q, *J* = 8.7 Hz, 4H), 6.65 (dd, *J* = 17.6, 10.9 Hz, 1H), 5.71 (dd, *J* = 17.6, 0.8 Hz, 1H), 5.25 (dd, *J* = 10.9, 0.7 Hz, 1H).

^{13}C NMR (100 MHz, $CDCl_3$) δ 136.14, 135.78, 133.54, 128.79, 127.55, 114.57.

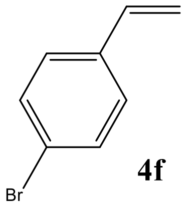

(4f)[5, 9] **4f**

^{1}H NMR (400 MHz, $CDCl_3$) δ 7.43 (d, *J* = 8.4 Hz, 2H), 7.25 (d, *J* = 11.0 Hz, 2H), 6.63 (dd, *J* = 17.6, 10.9 Hz, 1H), 5.72 (d, *J* = 17.6 Hz, 1H), 5.26 (d, *J* = 10.9 Hz, 1H).

^{13}C NMR (100 MHz, $CDCl_3$) δ 136.57, 135.84, 131.74, 127.88, 121.72, 114.73.

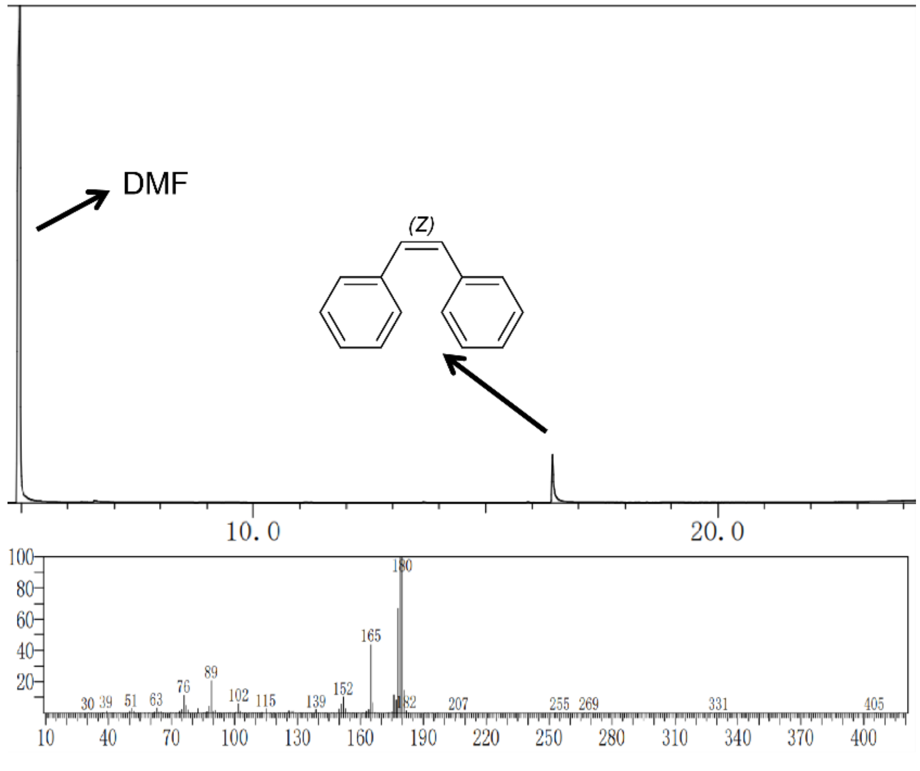

Figure S1: GC–MS chromatogram of diphenylacetylene reaction mixture after 48 h under standard conditions, showing no significant change in the (*Z*)-stilbene peak relative to the 24 h reaction. The peak labeled “DMF” corresponds to the solvent.

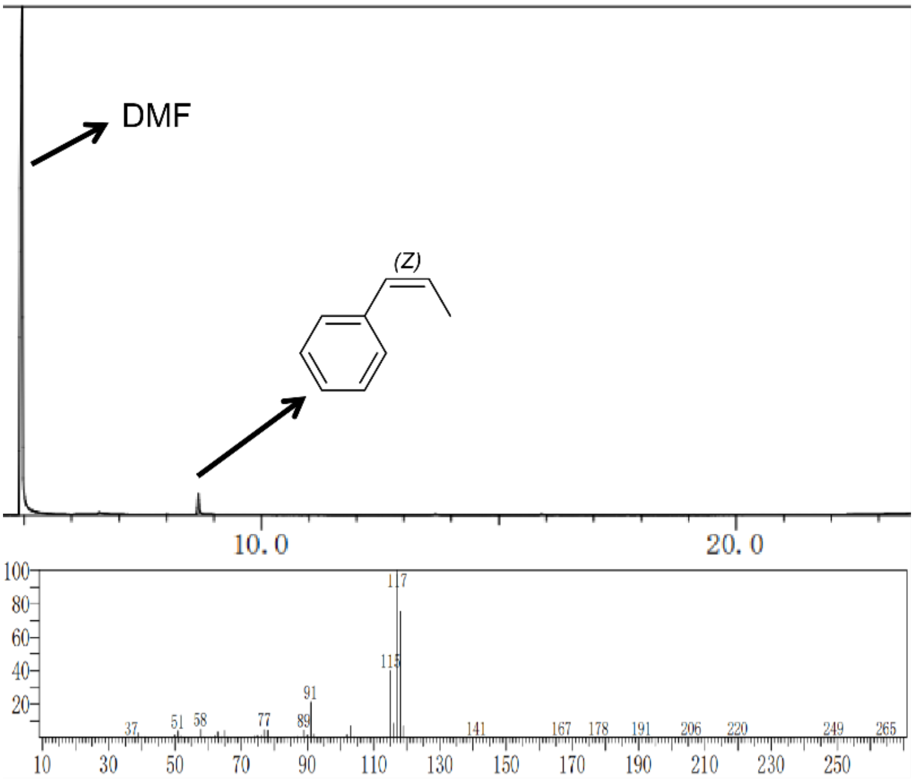

Figure S2: GC–MS chromatogram of the reaction mixture from 1-phenyl-1-propyne under standard conditions, showing complete conversion to (*Z*)-1-phenyl-1-propene (m/z = 117). The peak labeled “DMF” corresponds to the solvent.

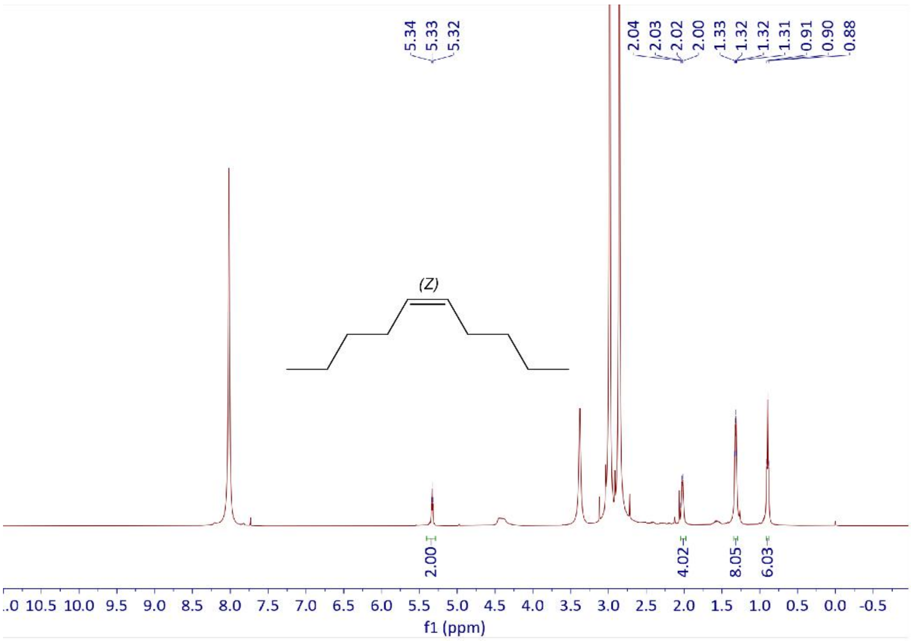

Figure S3: ^{1}H NMR spectrum of crude reaction mixture from 5-decyne after 24 h reaction, confirming the formation of (*Z*)-5-decene[1]. (After 24 h of catalytic reaction, the crude mixture was extracted with $CDCl_3$ for ^{1}H NMR measurement.)

References

(1) Li, J.; Hua, R.; Liu, T. *Journal of Organic Chemistry* **2010**, *75* (9), 2966-2970. DOI: 10.1021/jo100247a.

(2) Fu, S.; Chen, N.-Y.; Liu, X.; Shao, Z.; Luo, S.-P.; Liu, Q. *Journal of the American Chemical Society* **2016**, *138* (27), 8588-8594. DOI: 10.1021/jacs.6b04271.

(3) Wen, X.; Shi, X.; Qiao, X.; Wu, Z.; Bai, G. *Chemical Communications* **2017**, *53* (39), 5372-5375, 10.1039/C7CC02140B. DOI: 10.1039/C7CC02140B.

(4) Das, M.; O'Shea, D. F. *Organic Letters* **2016**, *18* (2), 336-339. DOI: 10.1021/acs.orglett.5b03519.

(5) Xia, S.; Peng, J.; Xie, S.; Xu, T.; Li, L.; Liu, X.; Cao, D.; He, L.-N.; Li, C.-J. *Organic Letters* **2025**, *27* (21), 5423-5428. DOI: 10.1021/acs.orglett.5c01372.

(6) Sheikh Mohammad, T.; Sakharov, P.; Raje, S.; de Ruiter, G. *ACS Catalysis* **2025**, *15* (7), 5370-5377. DOI: 10.1021/acscatal.5c00792.

(7) Chen, X.-B.; Zhang, J.; Sun, D.-Q.; Chen, K.-Q.; Chen, X.-Y. *Synthesis* **2025**, *57* (12), 1928-1935. DOI: 10.1055/a-2502-8374.

(8) Wang, Y.-H.; Liu, S.-T. *Polyhedron* **2025**, *265*, 117276. DOI: https://doi.org/10.1016/j.poly.2024.117276.

(9) Yan, Y.; Wang, P.; Han, J.; Wang, A.; Zhang, G.; Tian, Y.; Ge, Y.; Gao, W.; Wang, L.; Liu, Z.; et al. *Chemical Communications* **2025**, *61* (30), 5661-5664, 10.1039/D4CC06623E. DOI: 10.1039/D4CC06623E.

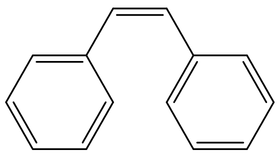

2a

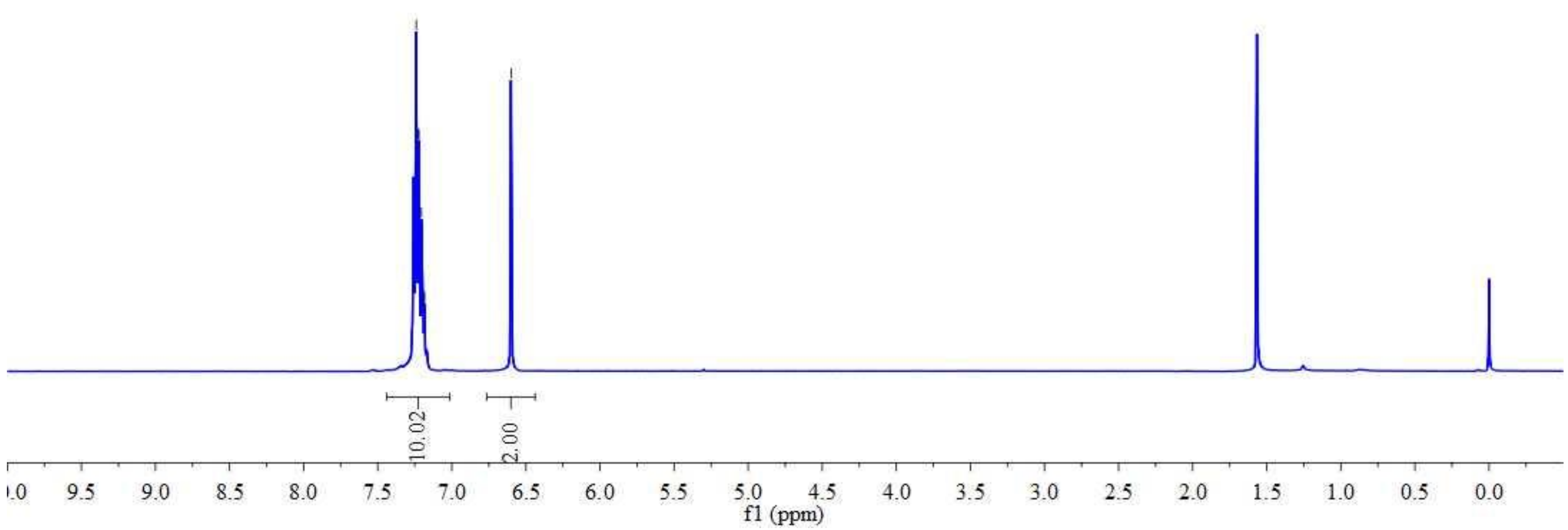

7.240
7.224
7.223
7.205
7.193
7.184
6.600
10.02
2.00
.0
9.5
9.0
8.5
8.0
7.5
7.0
6.5
6.0
5.5
5.0
4.5
4.0
3.5
3.0
2.5
2.0
1.5
1.0
0.5
0.0
f1 (ppm)

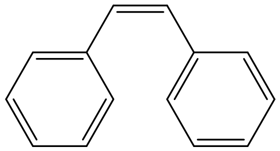

2a

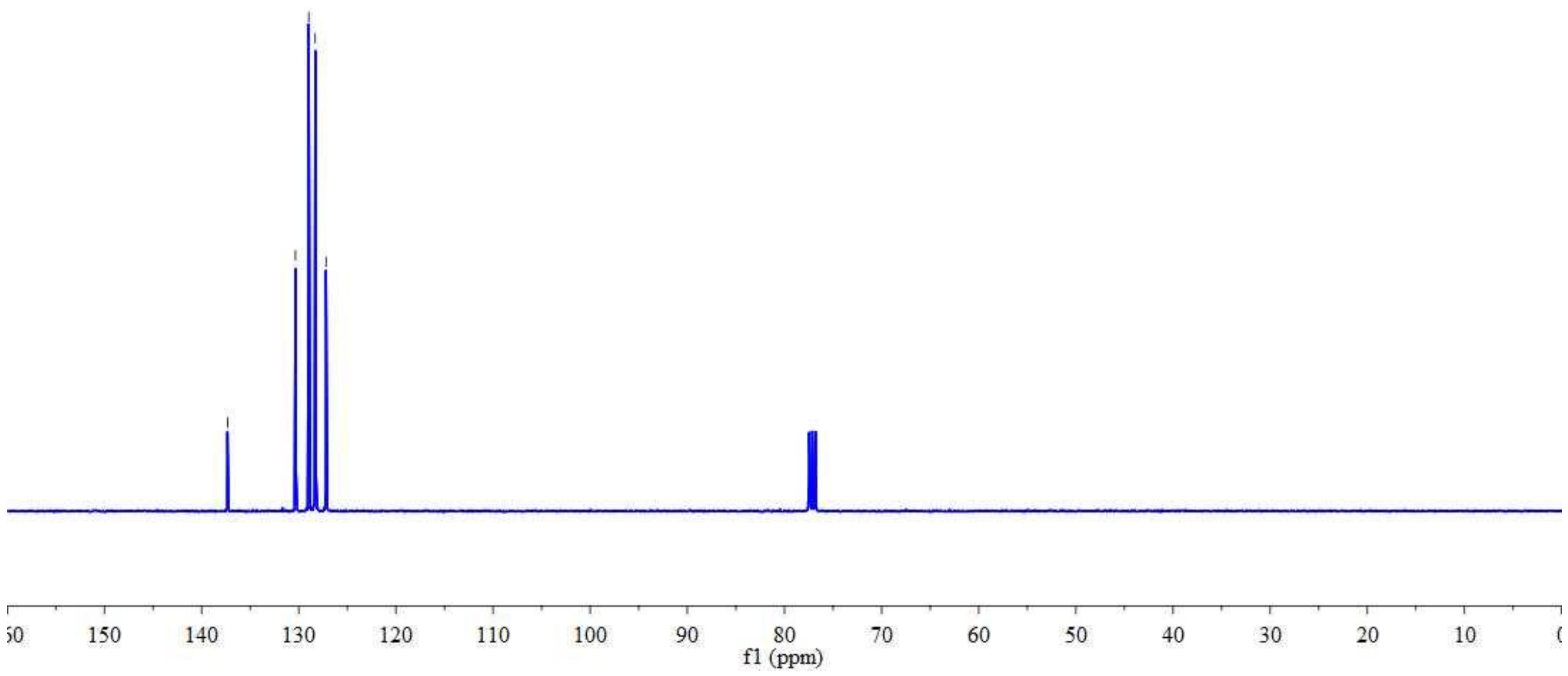

137.323
130.339
128.966
128.303
127.184
60
150
140
130
120
110
100
90
80
70
60
50
40
30
20
10
0
f1 (ppm)

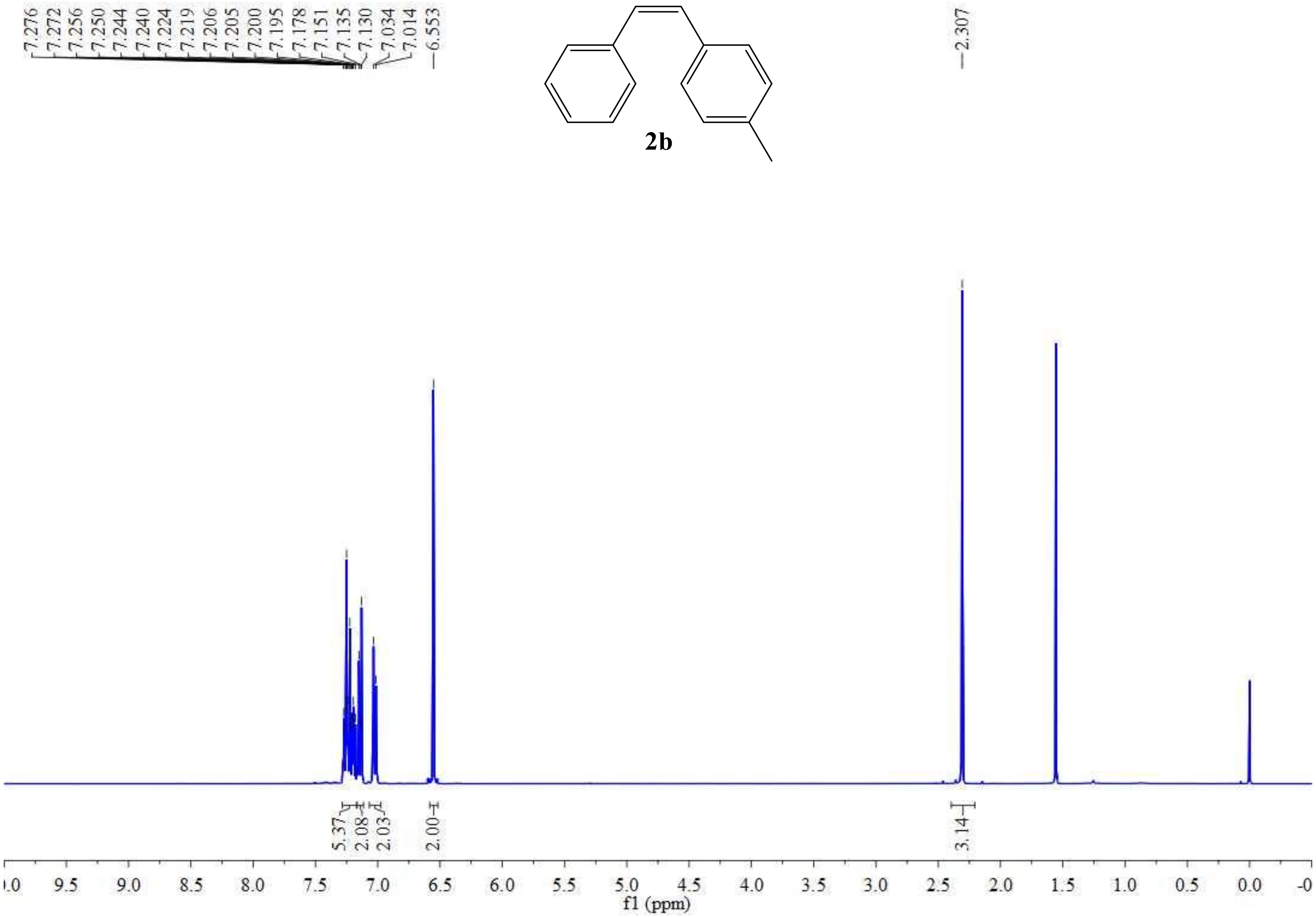
7.276
7.272
7.256
7.250
7.244
7.240
7.224
7.219
7.206
7.205
7.200
7.195
7.178
7.151
7.135
7.130
7.034
7.014
6.553
2b
2.307
5.37
2.08
2.03
2.00
3.14
f1 (ppm)

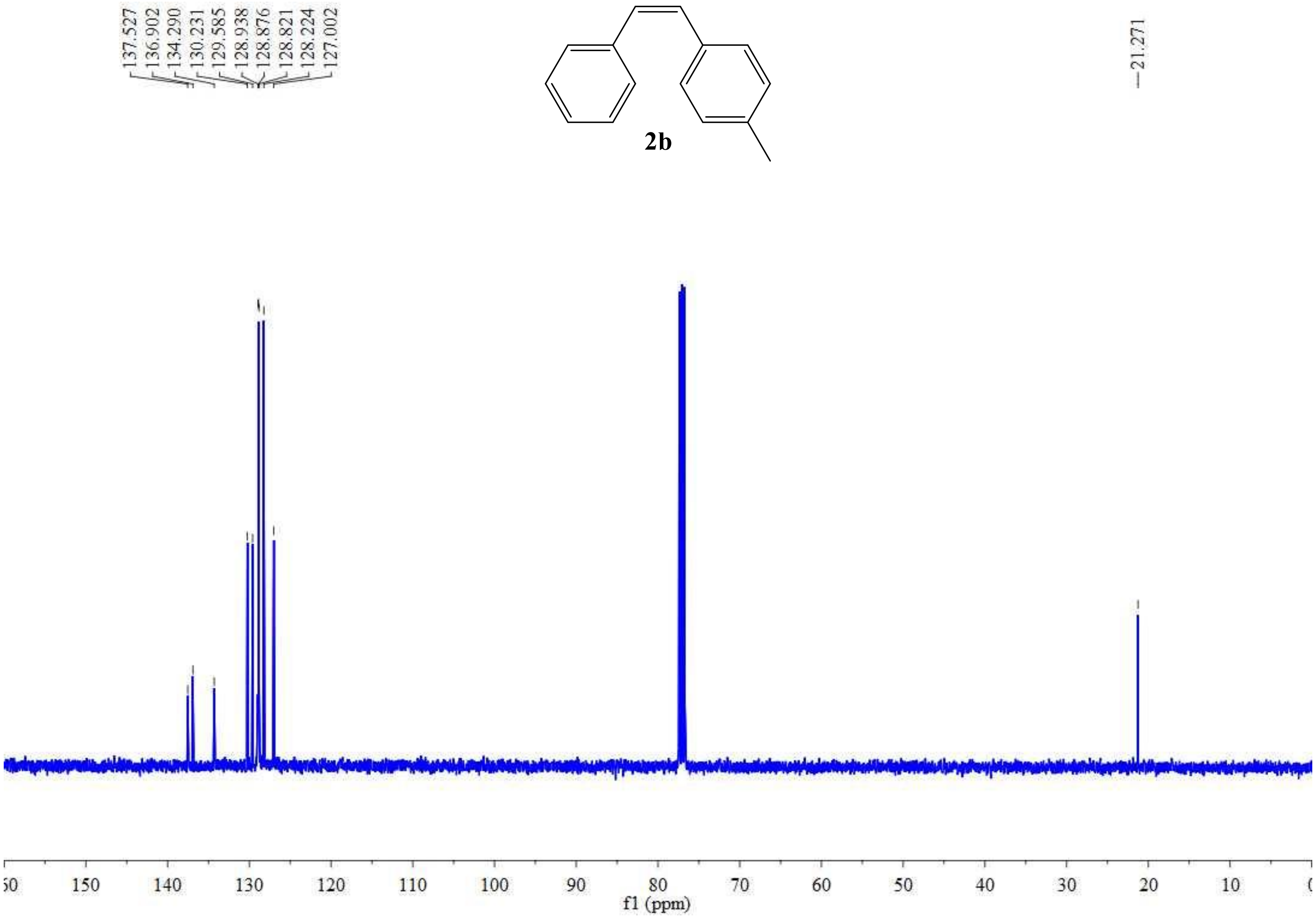
137.527
136.902
134.290
130.231
129.585
128.938
128.876
128.821
128.224
127.002
21.271
2b
150
140
130
120
110
100
90
80
70
60
50
40
30
20
10
f1 (ppm)

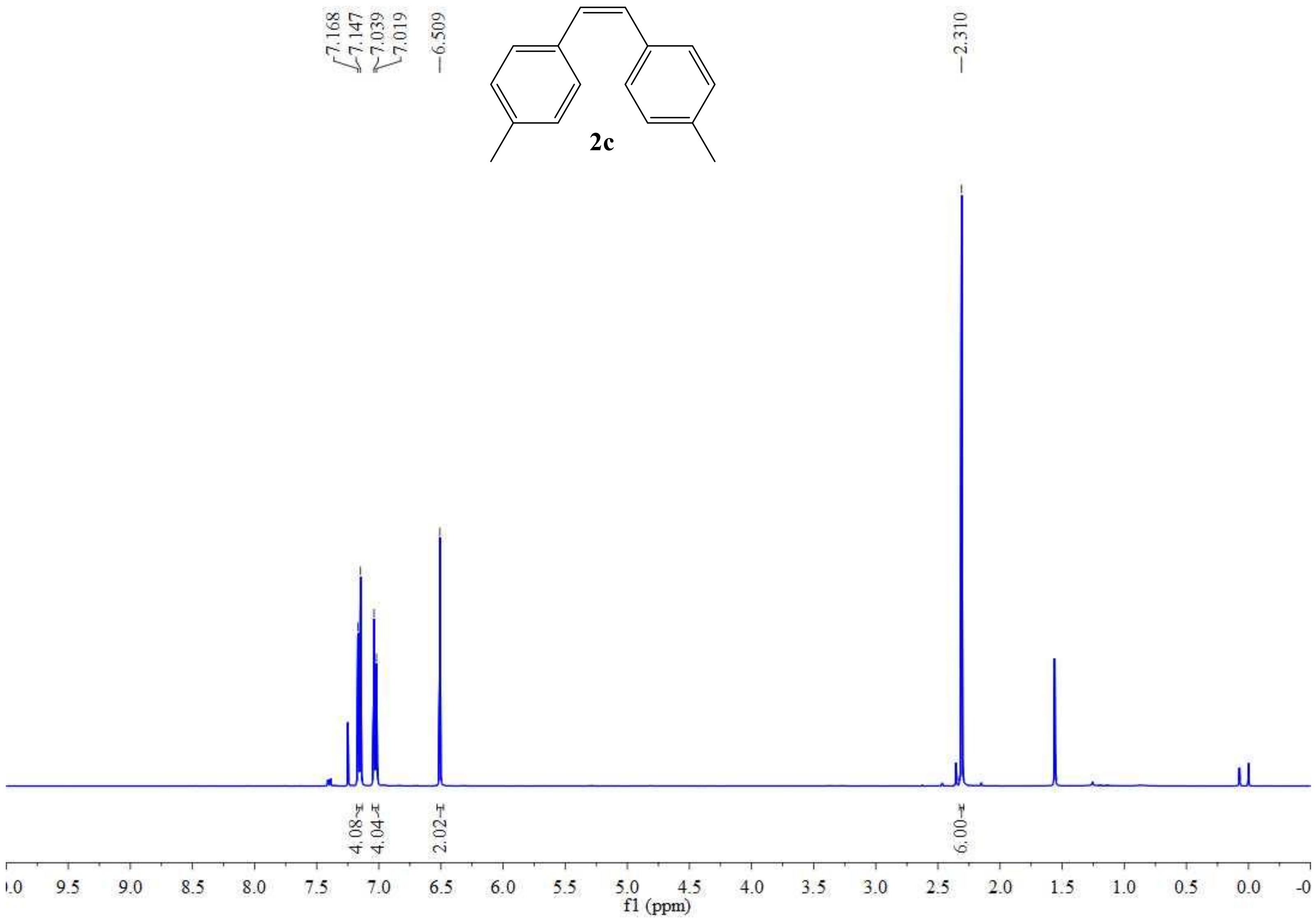

7.168
7.147
7.039
7.019
6.509
2.310
2c
4.08
4.04
2.02
6.00
9.5
9.0
8.5
8.0
7.5
7.0
6.5
6.0
5.5
5.0
4.5
4.0
3.5
3.0
2.5
2.0
1.5
1.0
0.5
0.0
f1 (ppm)

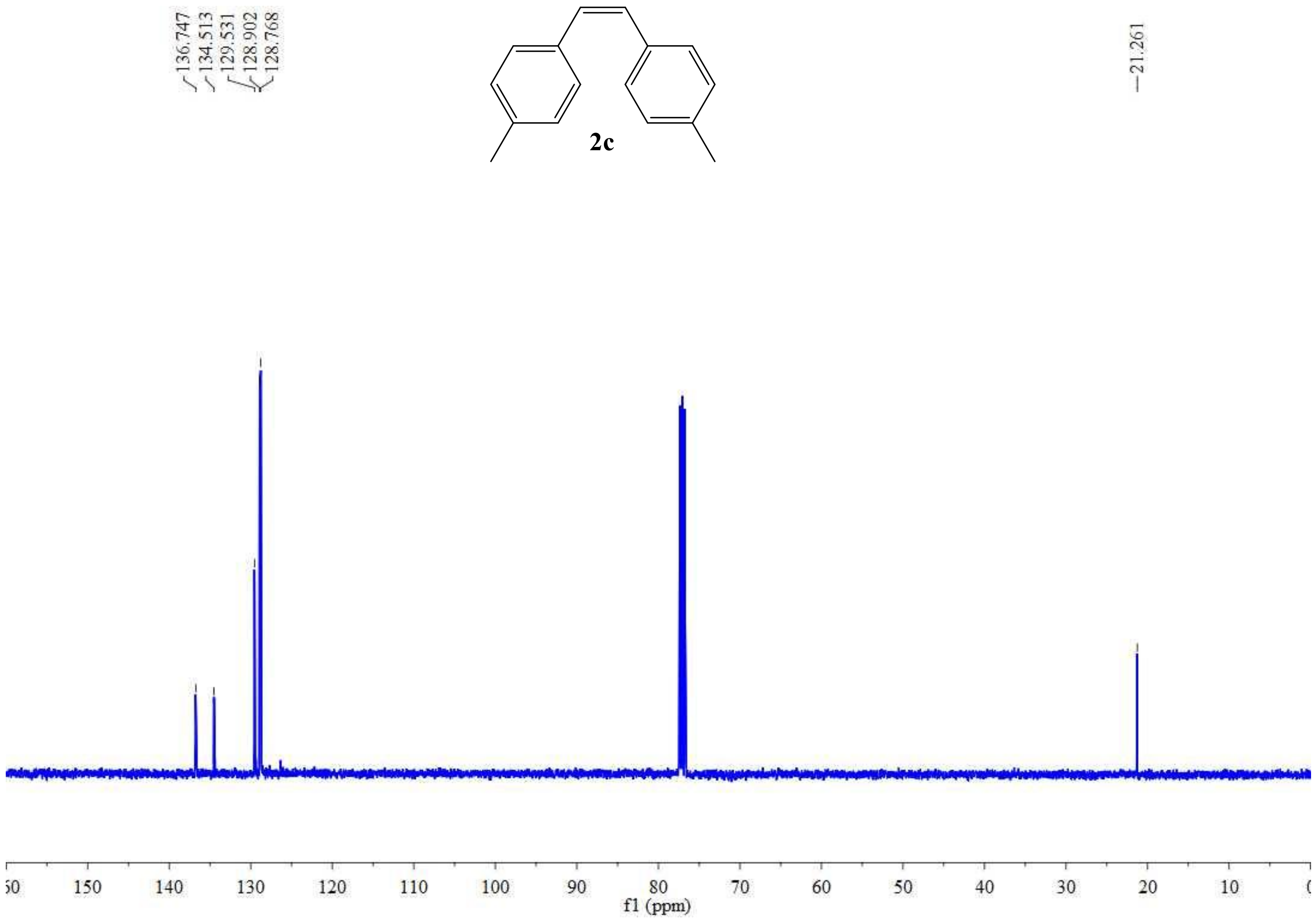
136.747
134.513
129.531
128.902
128.768
21.261
150
140
130
120
110
100
90
80
70
60
50
40
30
20
10
f1 (ppm)

2c

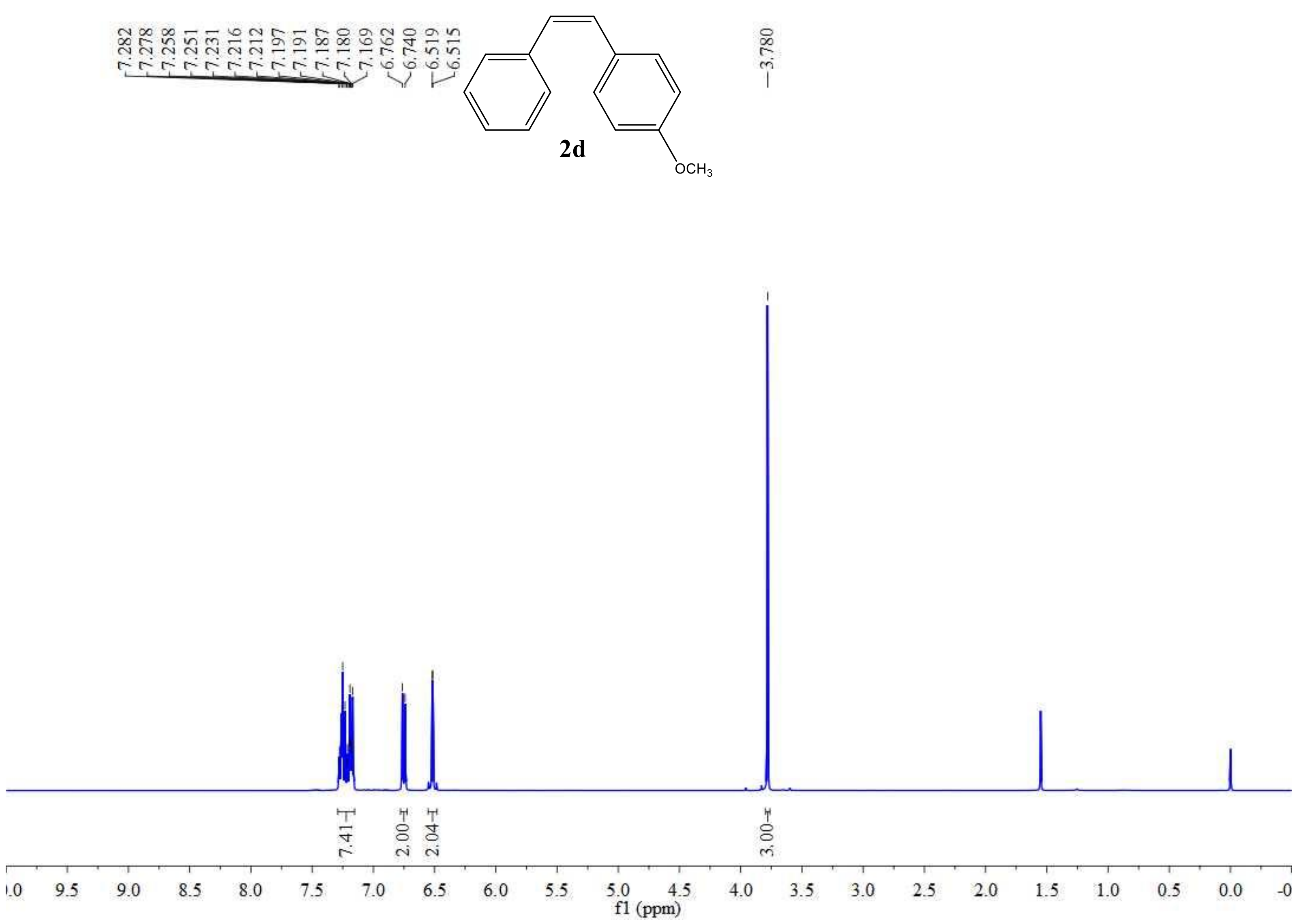
7.282
7.278
7.258
7.251
7.231
7.216
7.212
7.197
7.191
7.187
7.180
7.169
6.762
6.740
6.519
6.515
2d
OCH3
3.780
7.41
2.00
2.04
3.00
f1 (ppm)

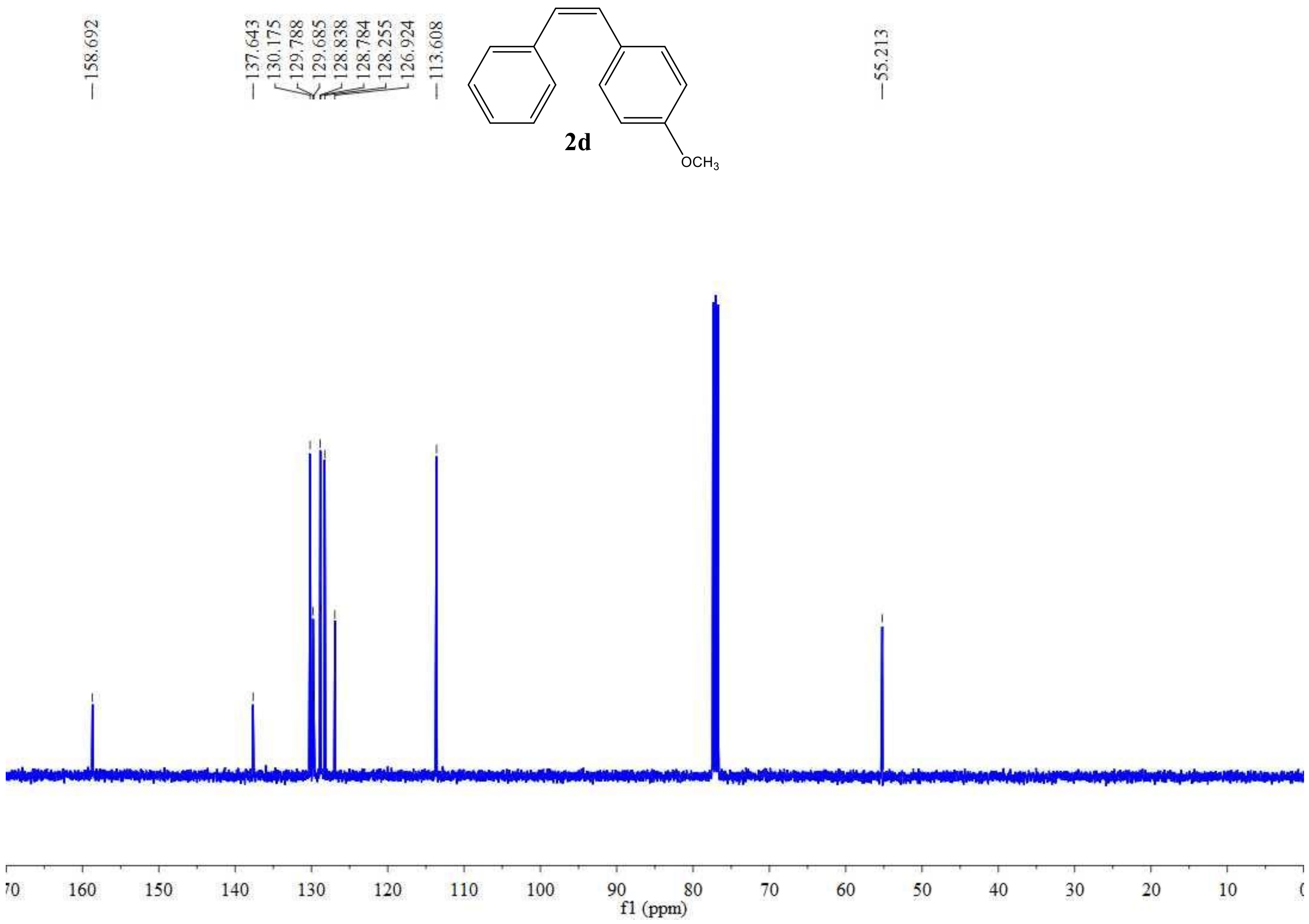

158.692
137.643
130.175
129.788
129.685
128.838
128.784
128.255
126.924
113.608
55.213
OCH3
70
160
150
140
130
120
110
100
90
80
70
60
50
40
30
20
10
f1 (ppm)

2d

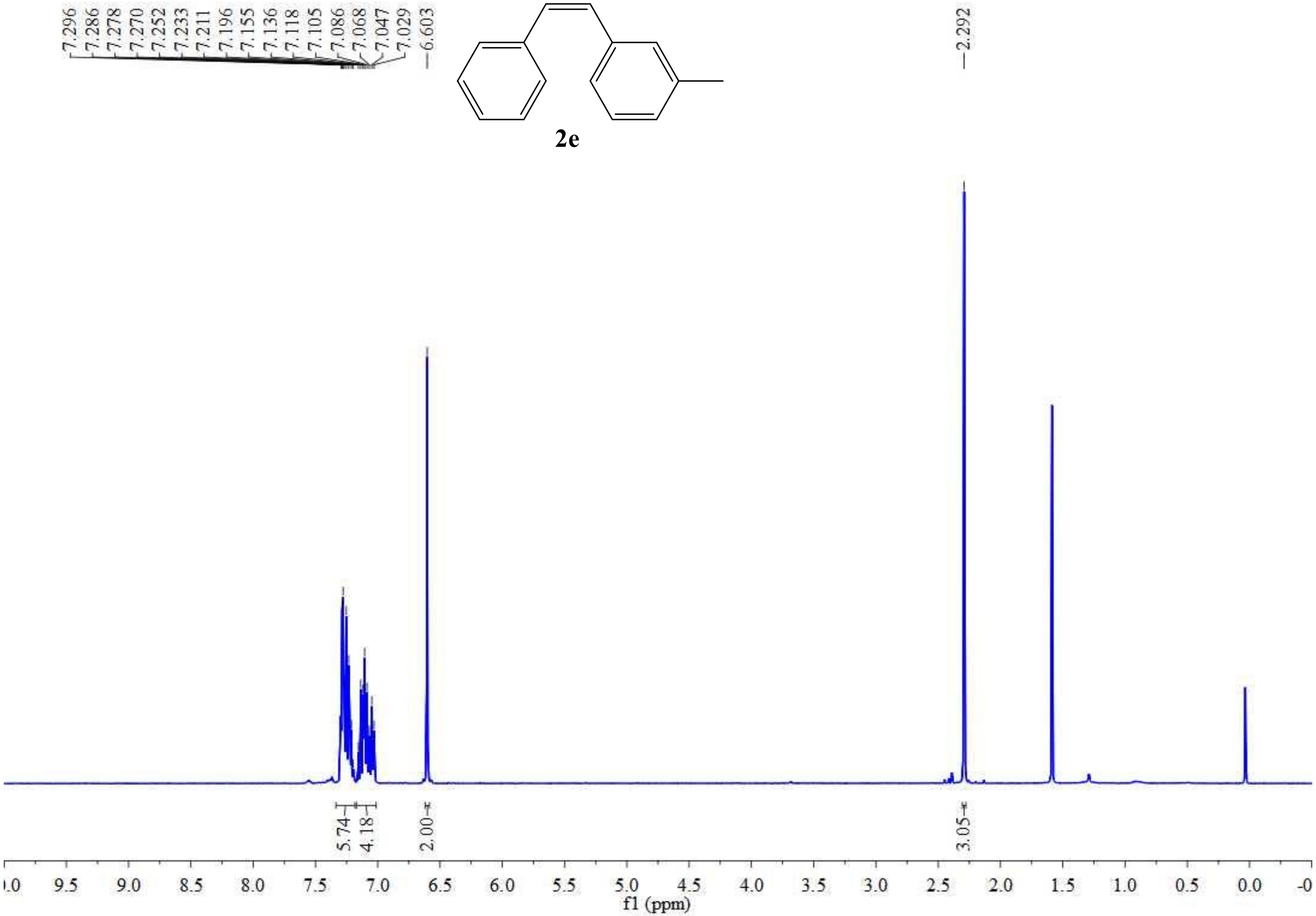
7.296
7.286
7.278
7.270
7.252
7.233
7.211
7.196
7.155
7.136
7.118
7.105
7.086
7.068
7.047
7.029
6.603
2.292
2e
5.74
4.18
2.00
3.05
9.5
9.0
8.5
8.0
7.5
7.0
6.5
6.0
5.5
5.0
4.5
4.0
3.5
3.0
2.5
2.0
1.5
1.0
0.5
0.0
f1 (ppm)

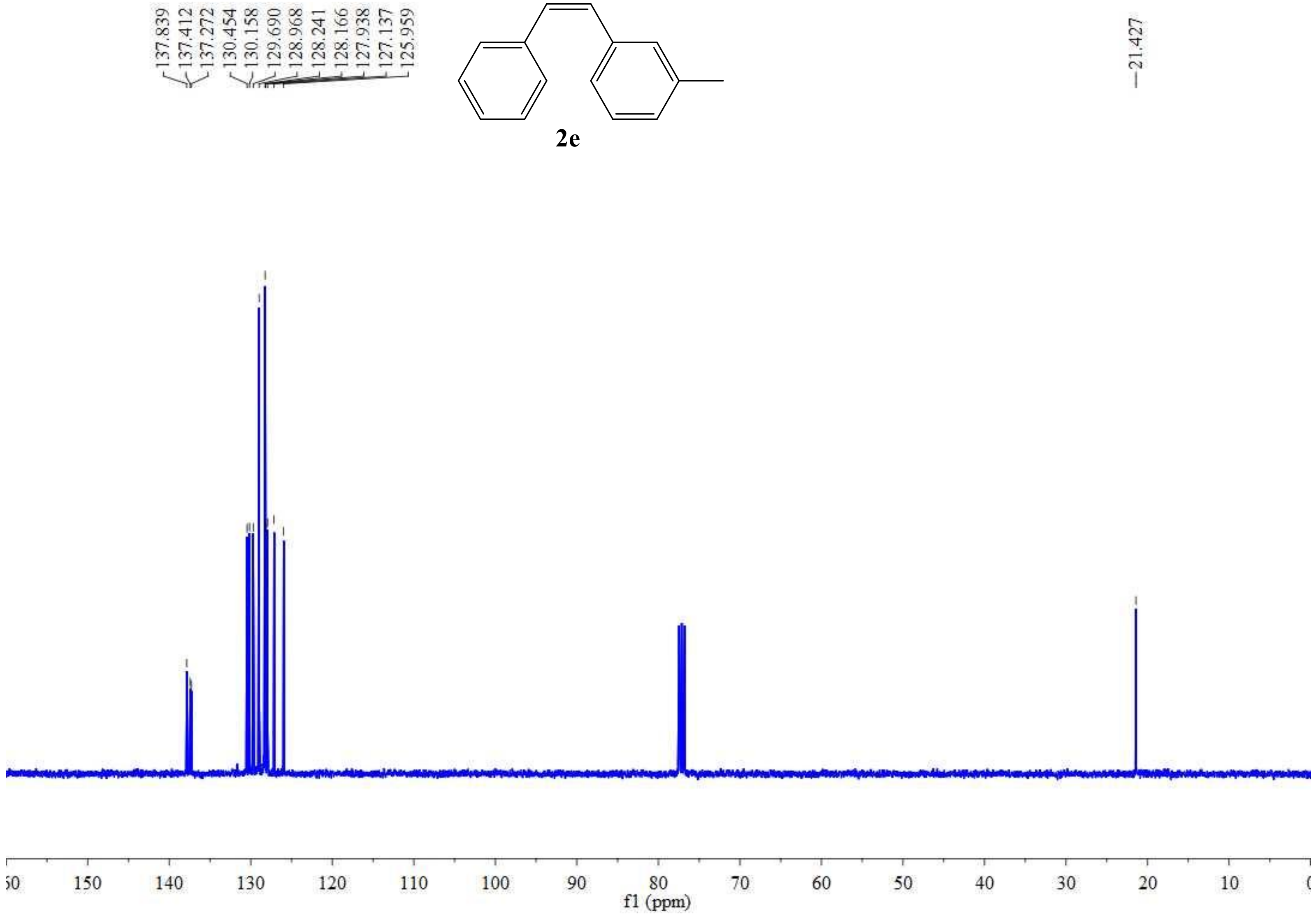
137.839
137.412
137.272
130.454
130.158
129.690
128.968
128.241
128.166
127.938
127.137
125.959
2e
21.427
150
140
130
120
110
100
90
80
70
60
50
40
30
20
10
f1 (ppm)

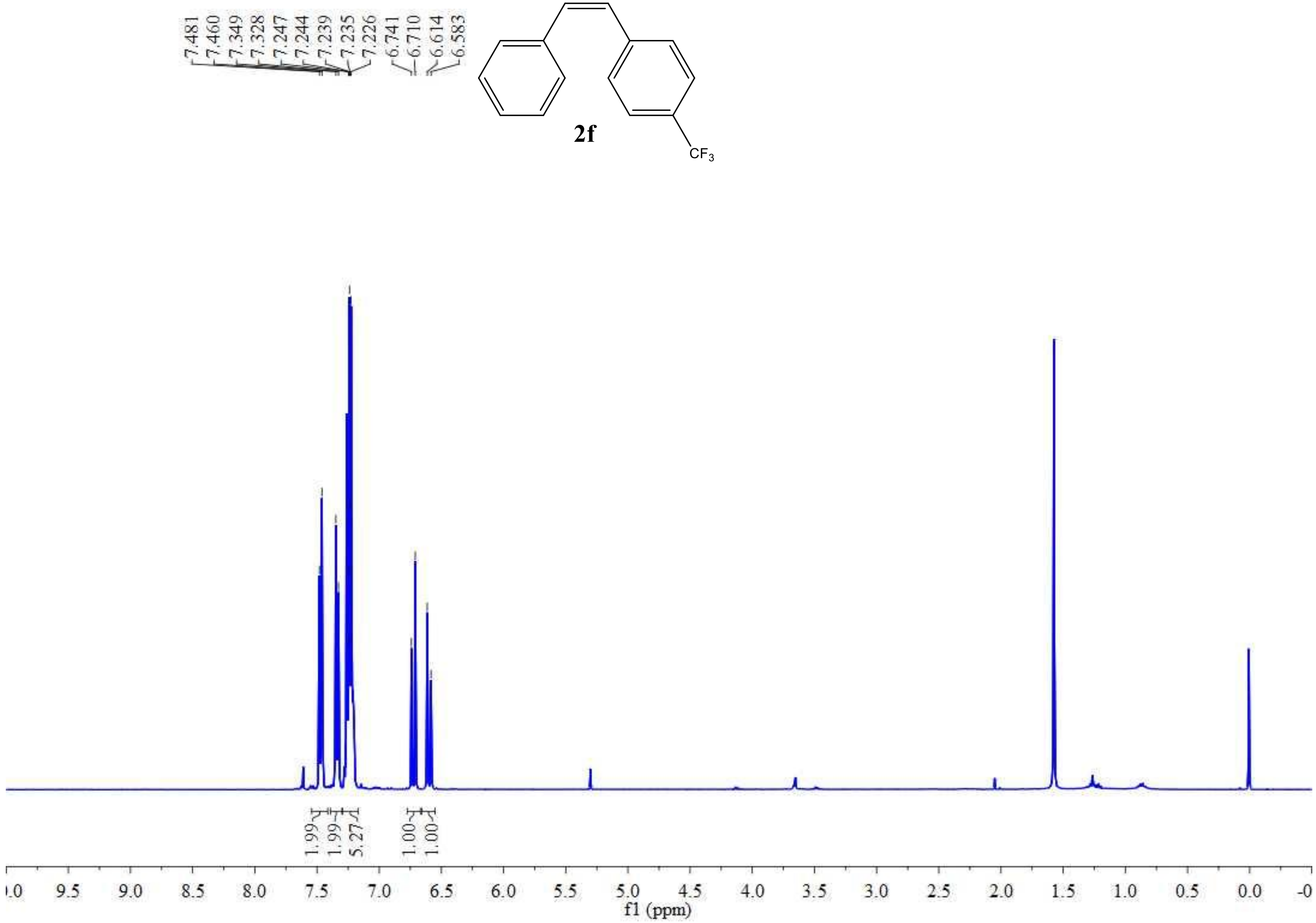
7.481
7.460
7.349
7.328
7.247
7.244
7.239
7.235
7.226
6.741
6.710
6.614
6.583
2f
CF3
1.99
1.99
5.27
1.00
1.00
.0
9.5
9.0
8.5
8.0
7.5
7.0
6.5
6.0
5.5
5.0
4.5
4.0
3.5
3.0
2.5
2.0
1.5
1.0
0.5
0.0
-0
f1 (ppm)

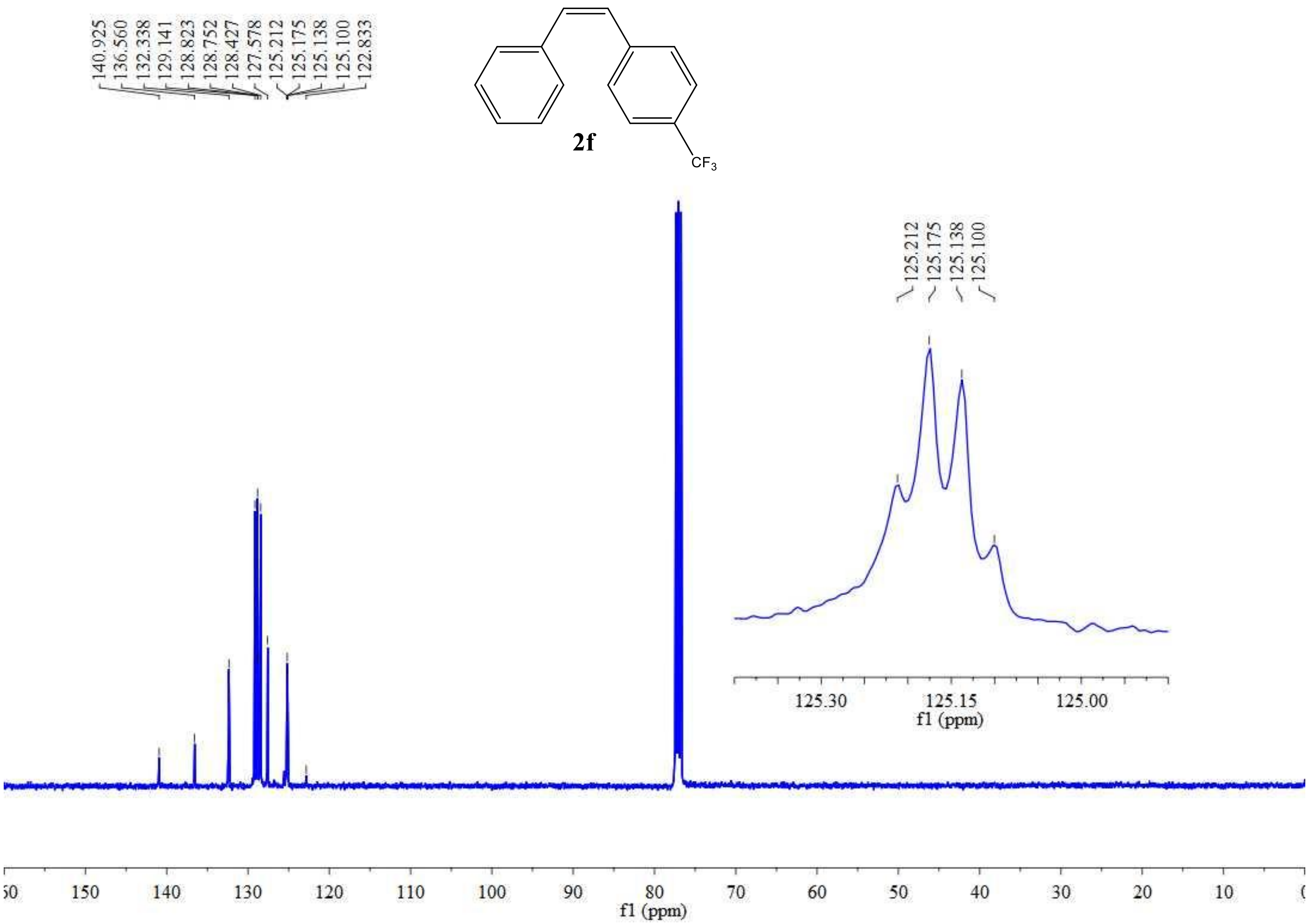

140.925
136.560
132.338
129.141
128.823
128.752
128.427
127.578
125.212
125.175
125.138
125.100
122.833
2f
CF3
125.212
125.175
125.138
125.100
125.30
125.15
125.00
f1 (ppm)
150
140
130
120
110
100
90
80
70
60
50
40
30
20
10
f1 (ppm)

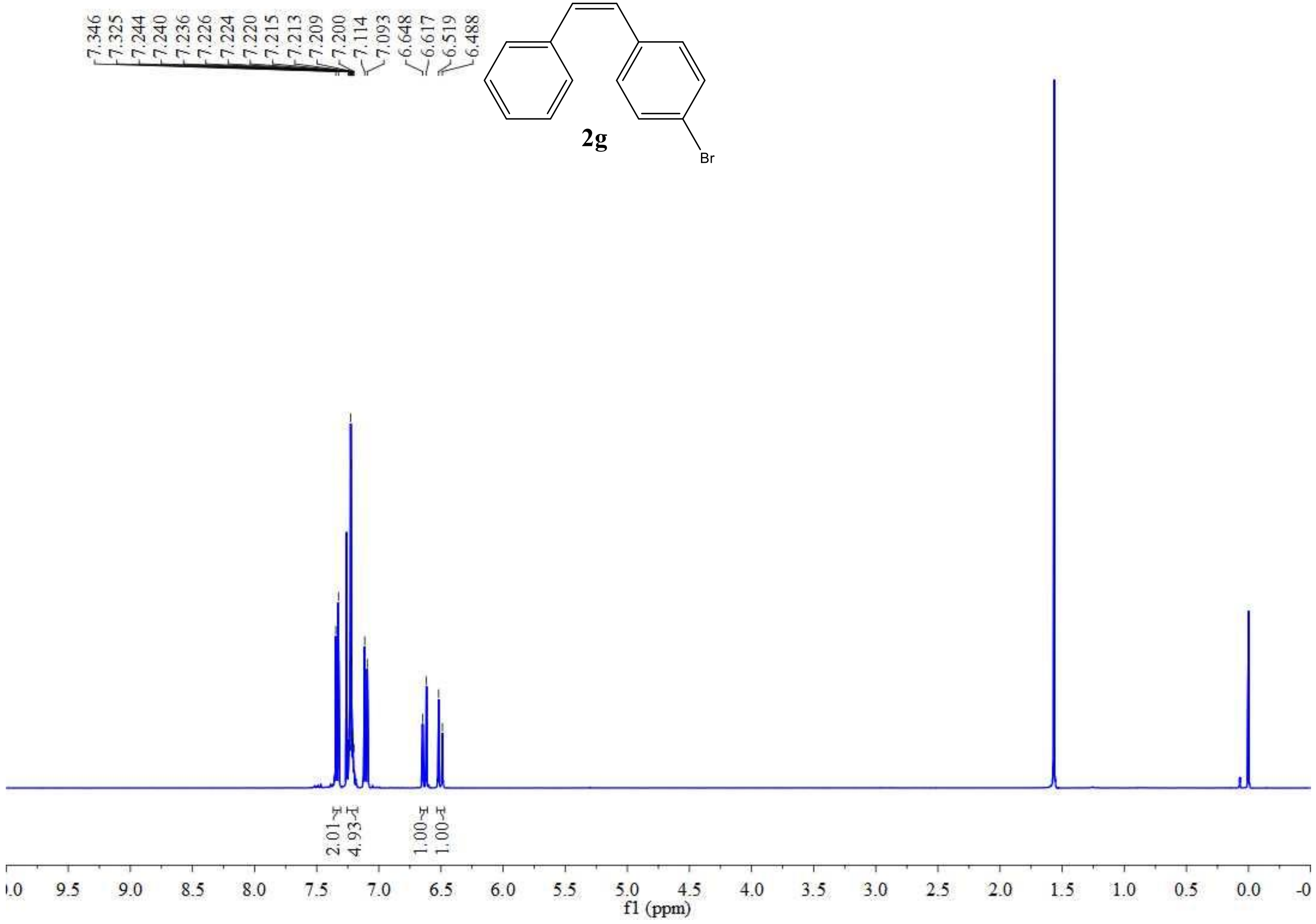
7.346
7.325
7.244
7.240
7.236
7.226
7.224
7.220
7.215
7.213
7.209
7.200
7.114
7.093
6.648
6.617
6.519
6.488
2g
Br
2.01
4.93
1.00
1.00
f1 (ppm)

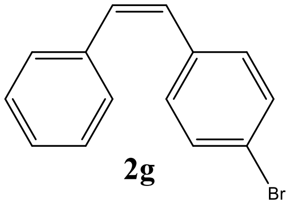

2g

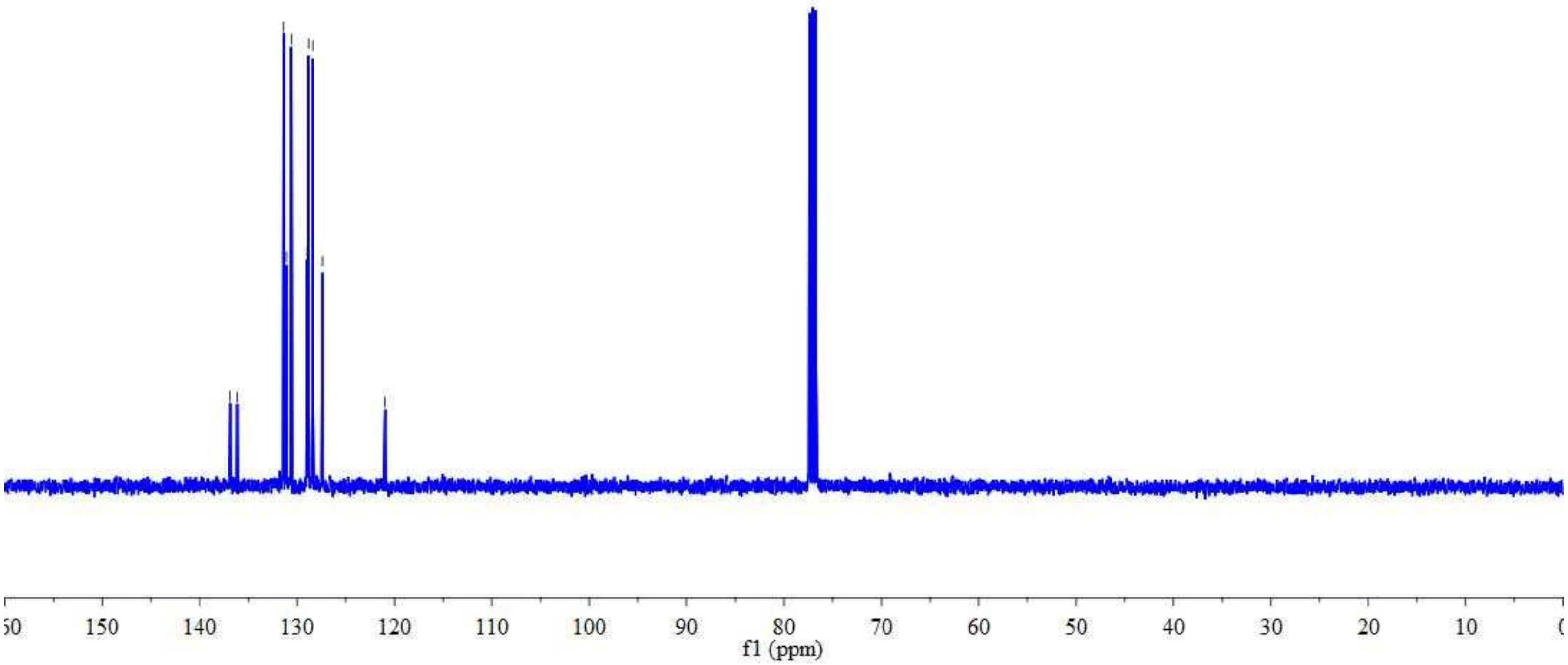
136.860
136.135
131.393
131.066
130.569
128.973
128.826
128.385
127.377
120.963
f1 (ppm)

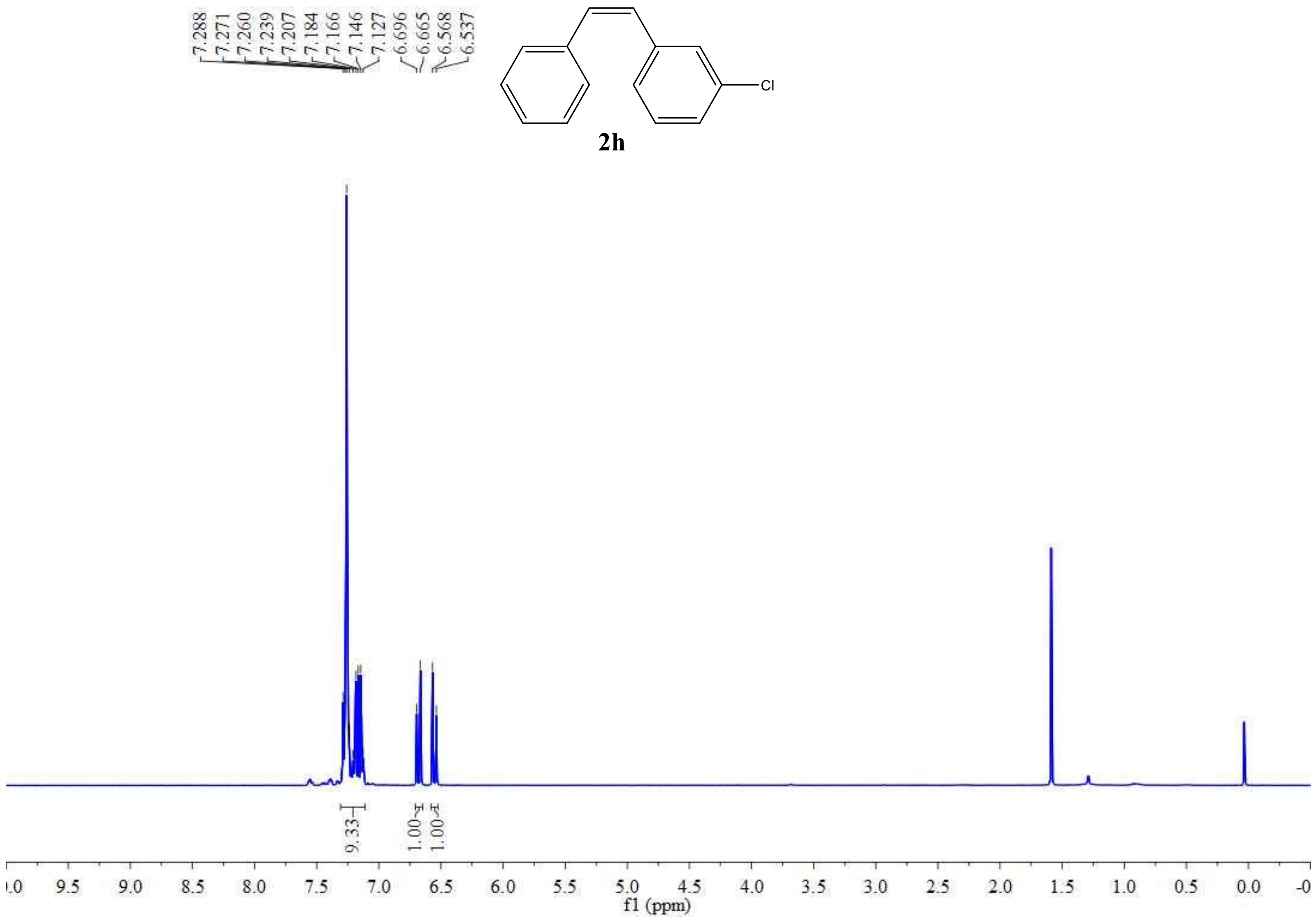

7.288
7.271
7.260
7.239
7.207
7.184
7.166
7.146
7.127
6.696
6.665
6.568
6.537
Cl
2h
9.33
1.00
1.00
f1 (ppm)

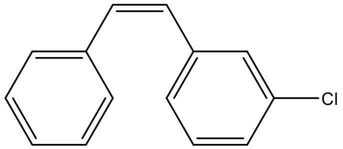

2h

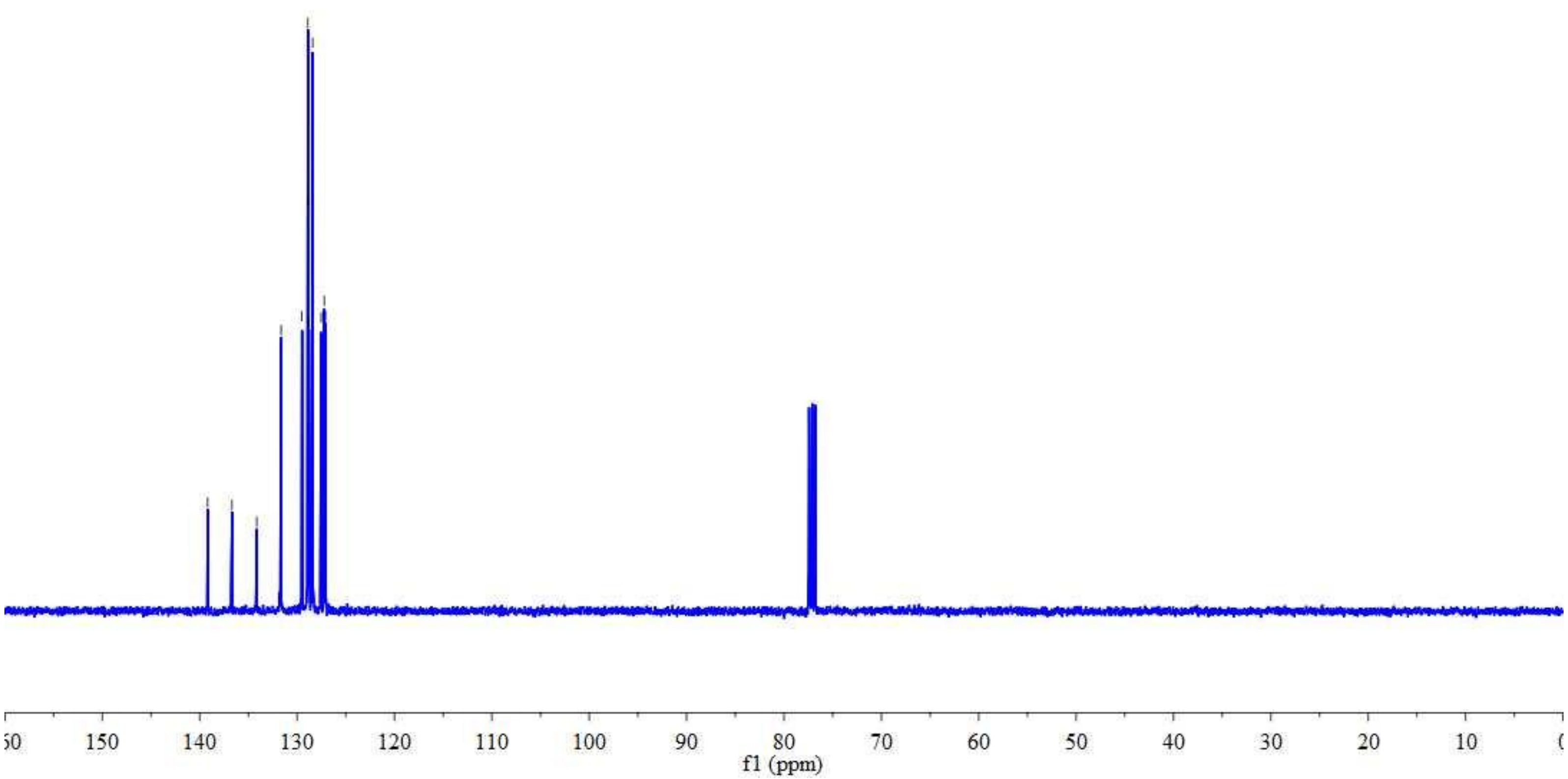
139.171
136.679
134.135
131.638
129.499
128.909
128.889
128.784
128.397
127.520
127.202
127.070
150
140
130
120
110
100
90
80
70
60
50
40
30
20
10
f1 (ppm)

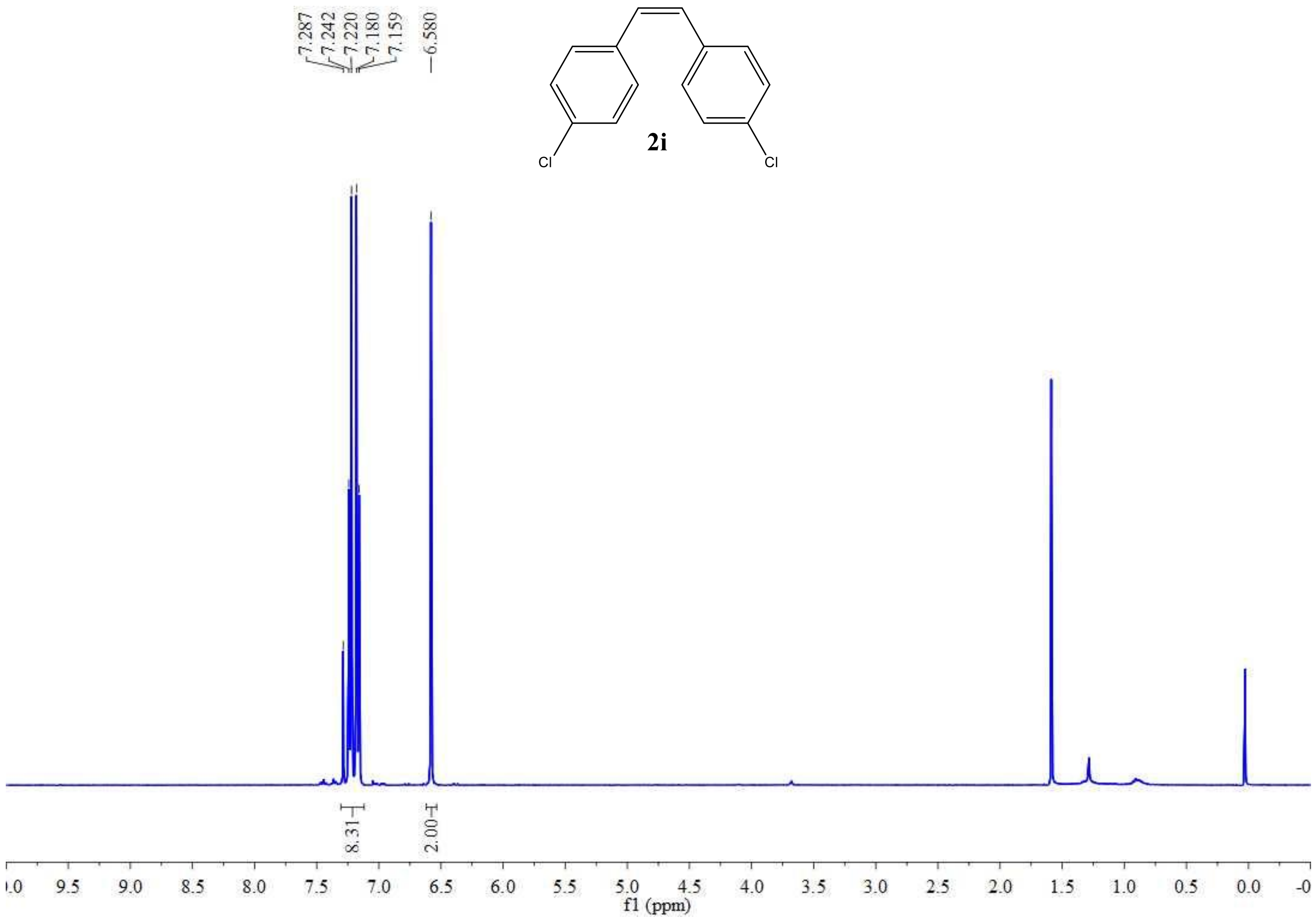
7.287
7.242
7.220
7.180
7.159
6.580
Cl
2i
Cl
8.31
2.00
1.0
9.5
9.0
8.5
8.0
7.5
7.0
6.5
6.0
5.5
5.0
4.5
4.0
3.5
3.0
2.5
2.0
1.5
1.0
0.5
0.0
-0
f1 (ppm)

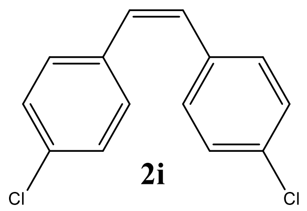
Cl
Cl

2i

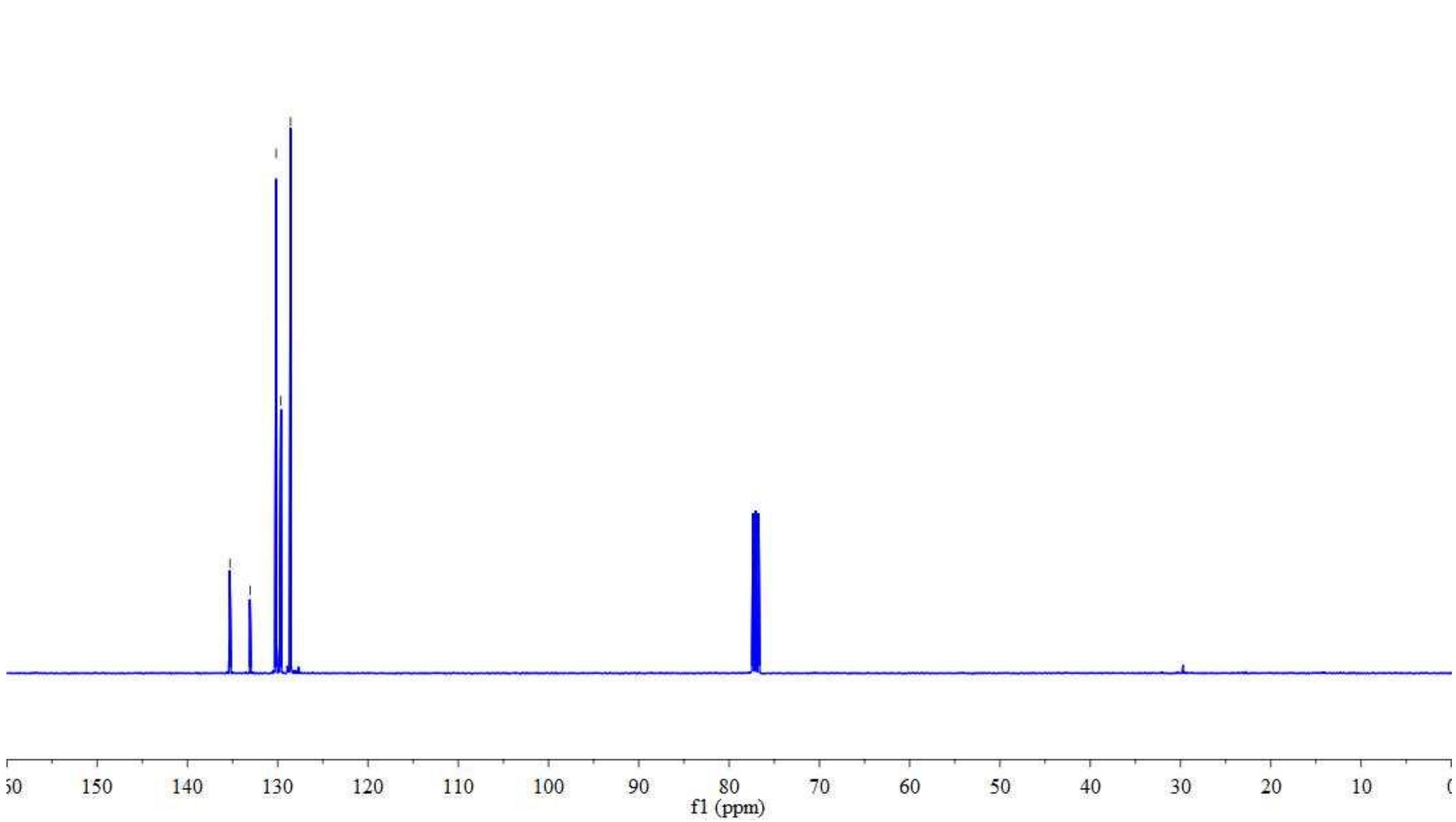
135.290
133.062
130.170
129.641
128.576
150
140
130
120
110
100
90
80
70
60
50
40
30
20
10
f1 (ppm)

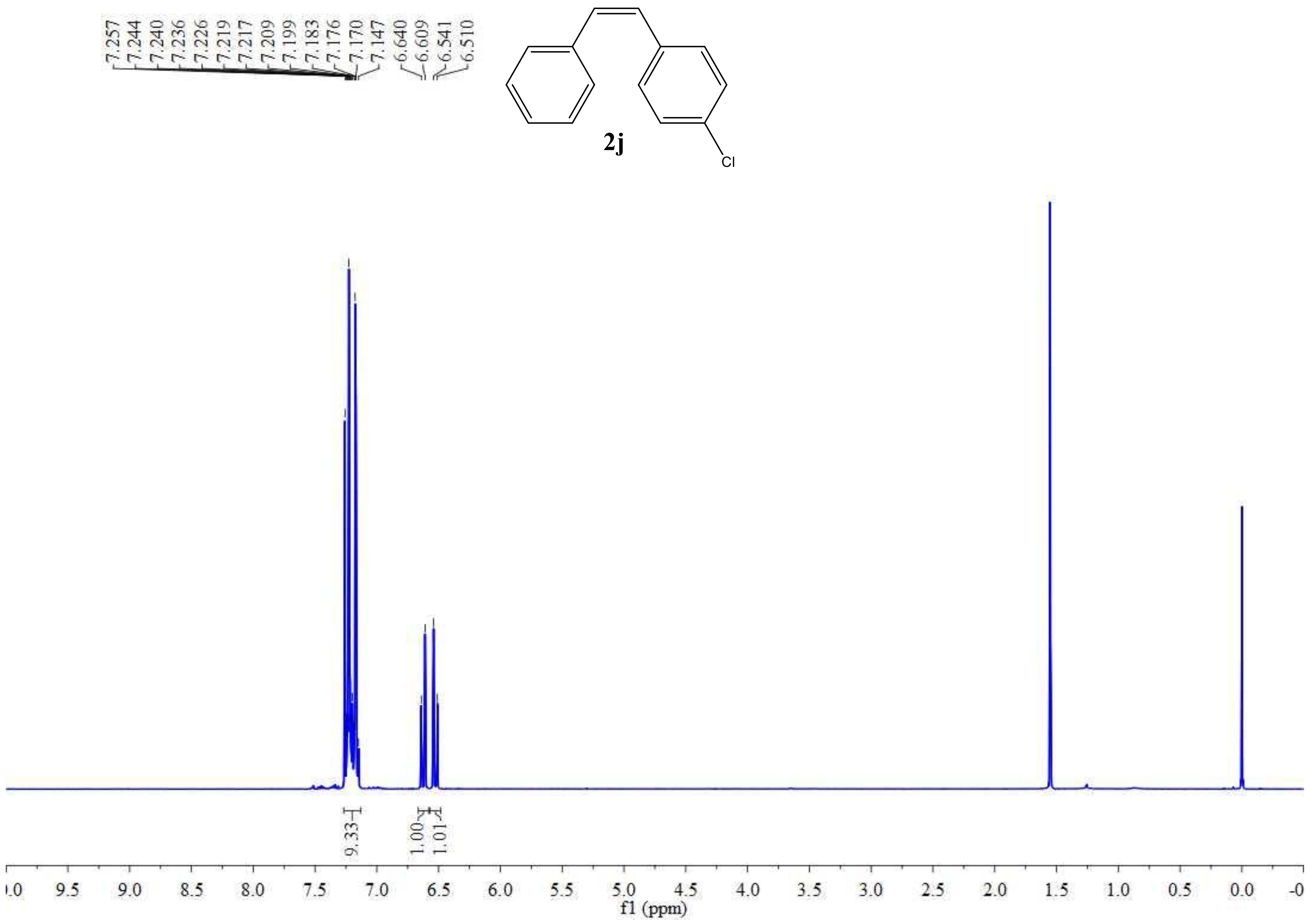
7.257
7.244
7.240
7.236
7.226
7.219
7.217
7.209
7.199
7.183
7.176
7.170
7.147
6.640
6.609
6.541
6.510
Cl
2j
9.33
1.00
1.01
1.0
9.5
9.0
8.5
8.0
7.5
7.0
6.5
6.0
5.5
5.0
4.5
4.0
3.5
3.0
2.5
2.0
1.5
1.0
0.5
0.0
-0
f1 (ppm)

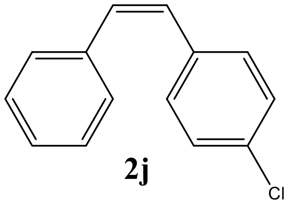
Cl

2j

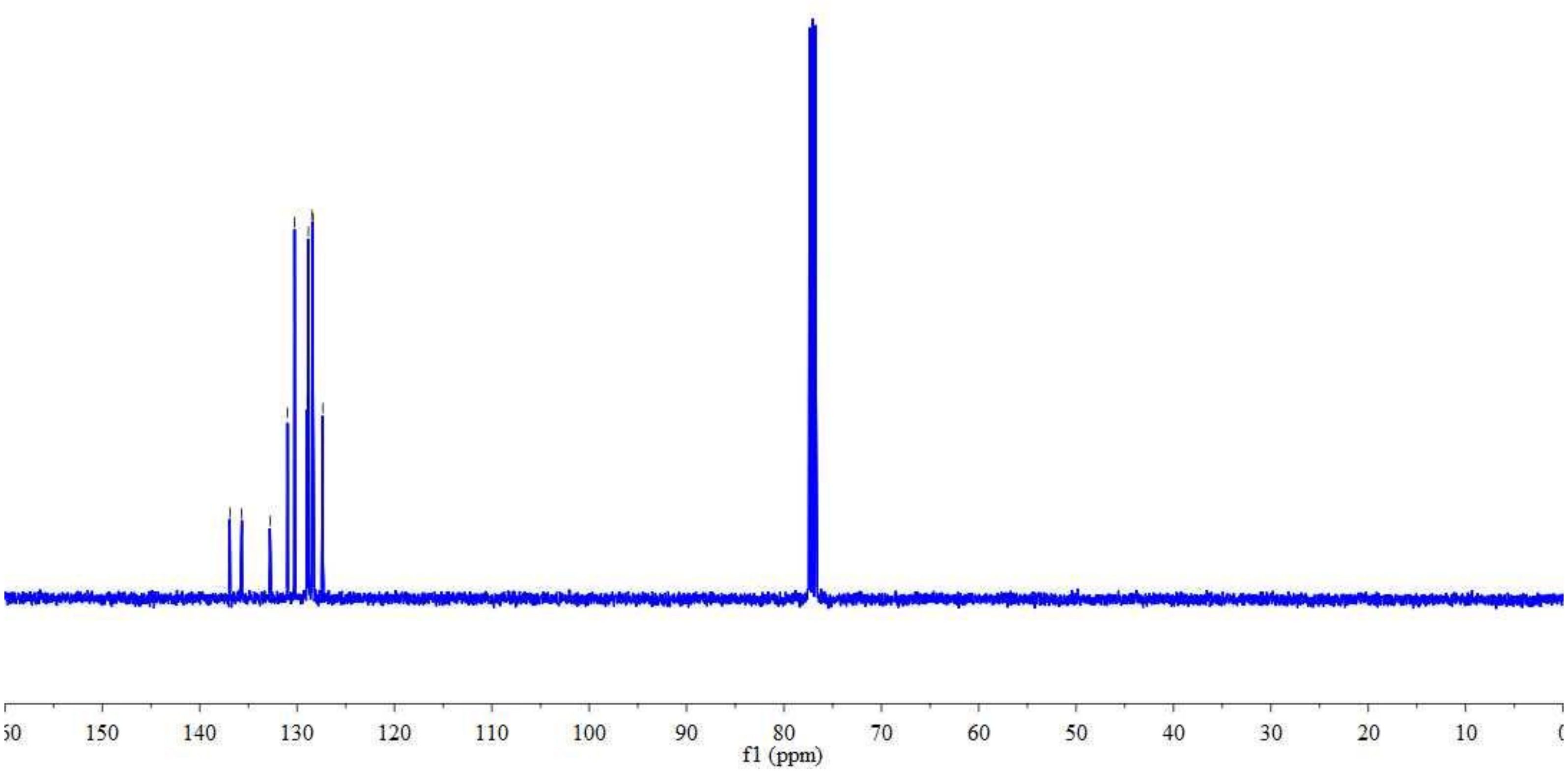
136.897
135.677
132.774
130.983
130.248
128.953
128.838
128.441
128.373
127.353
150
140
130
120
110
100
90
80
70
60
50
40
30
20
10
f1 (ppm)

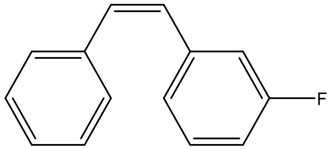

2k

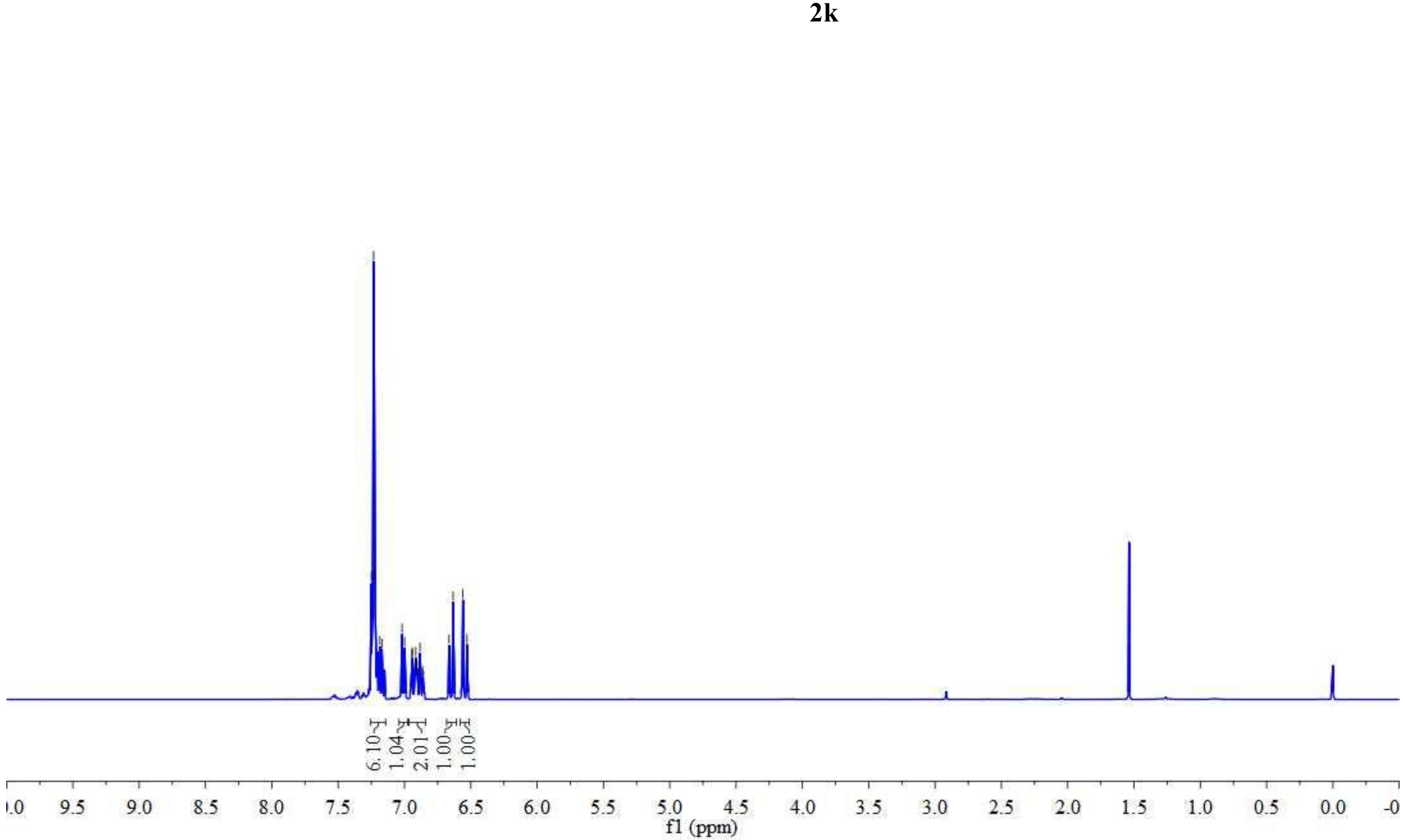
7.250
7.247
7.242
7.232
7.223
7.186
7.170
7.018
6.999
6.944
6.940
6.915
6.908
6.905
6.898
6.883
6.862
6.856
6.664
6.633
6.559
6.529
6.10
1.04
2.01
1.00
1.00
9.5
9.0
8.5
8.0
7.5
7.0
6.5
6.0
5.5
5.0
4.5
4.0
3.5
3.0
2.5
2.0
1.5
1.0
0.5
0.0
f1 (ppm)

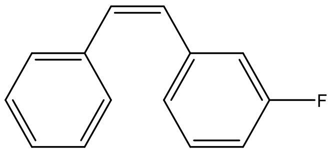

2k

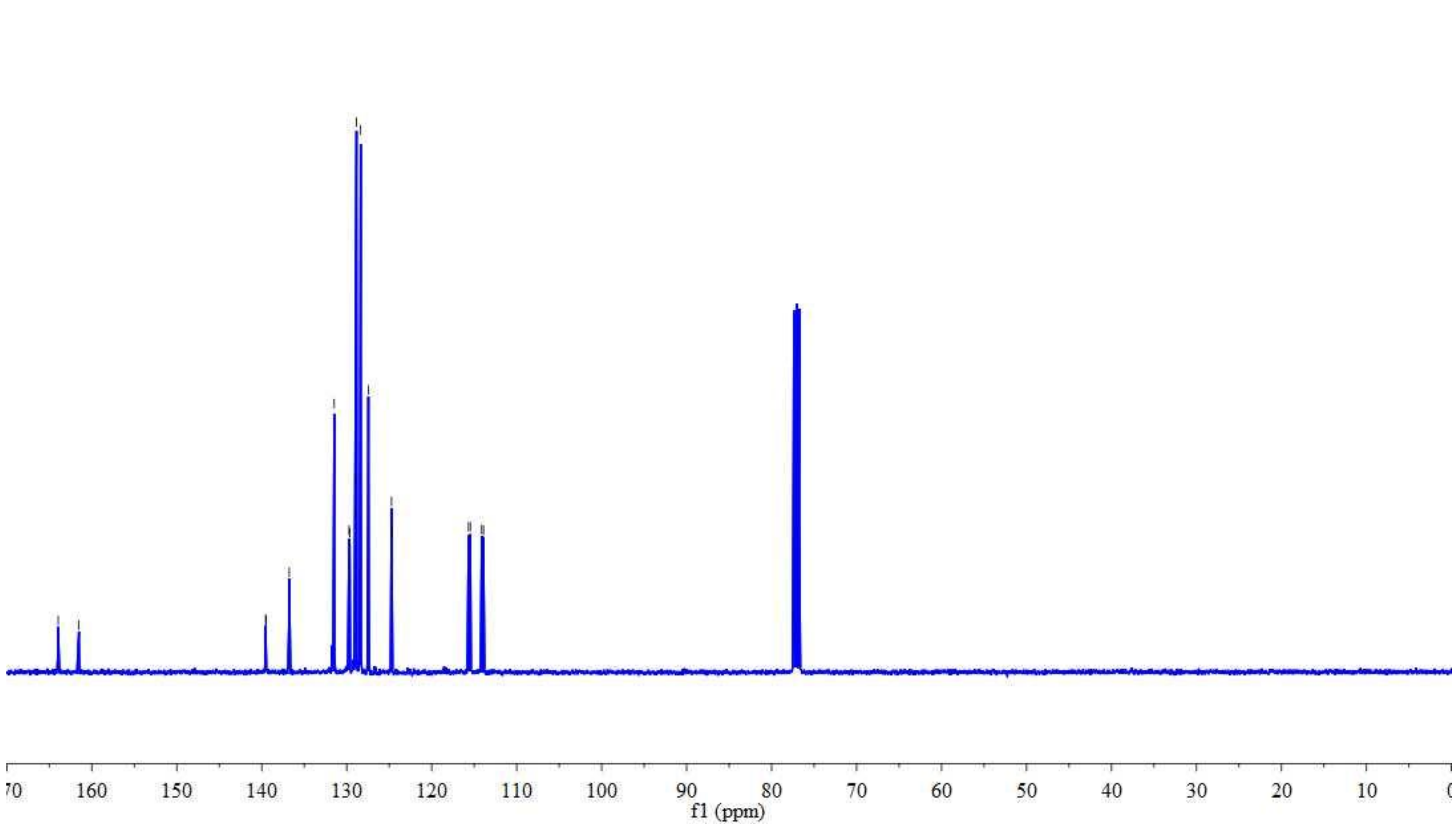
163.950
161.513
139.546
139.468
136.756
131.469
129.732
129.649
129.013
128.991
128.850
128.365
127.446
124.730
124.703
115.668
115.452
114.113
113.902
f1 (ppm)

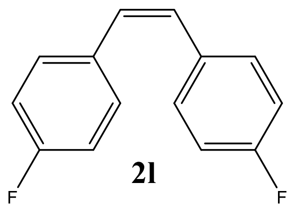

2l

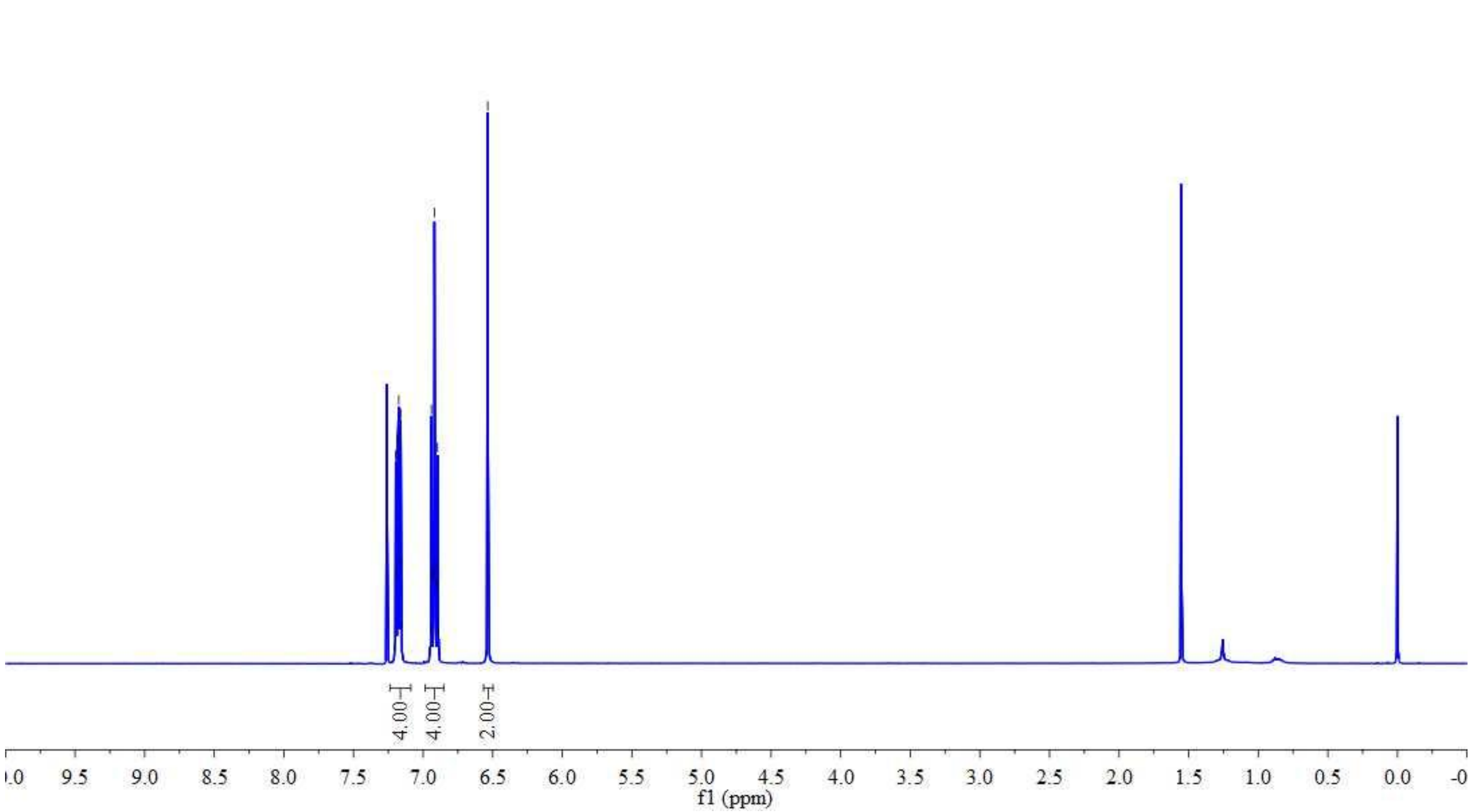
7.197
7.191
7.183
7.175
7.167
7.161
7.154
6.948
6.940
6.935
6.924
6.918
6.913
6.902
6.896
6.889
6.535
4.00
4.00
2.00
f1 (ppm)

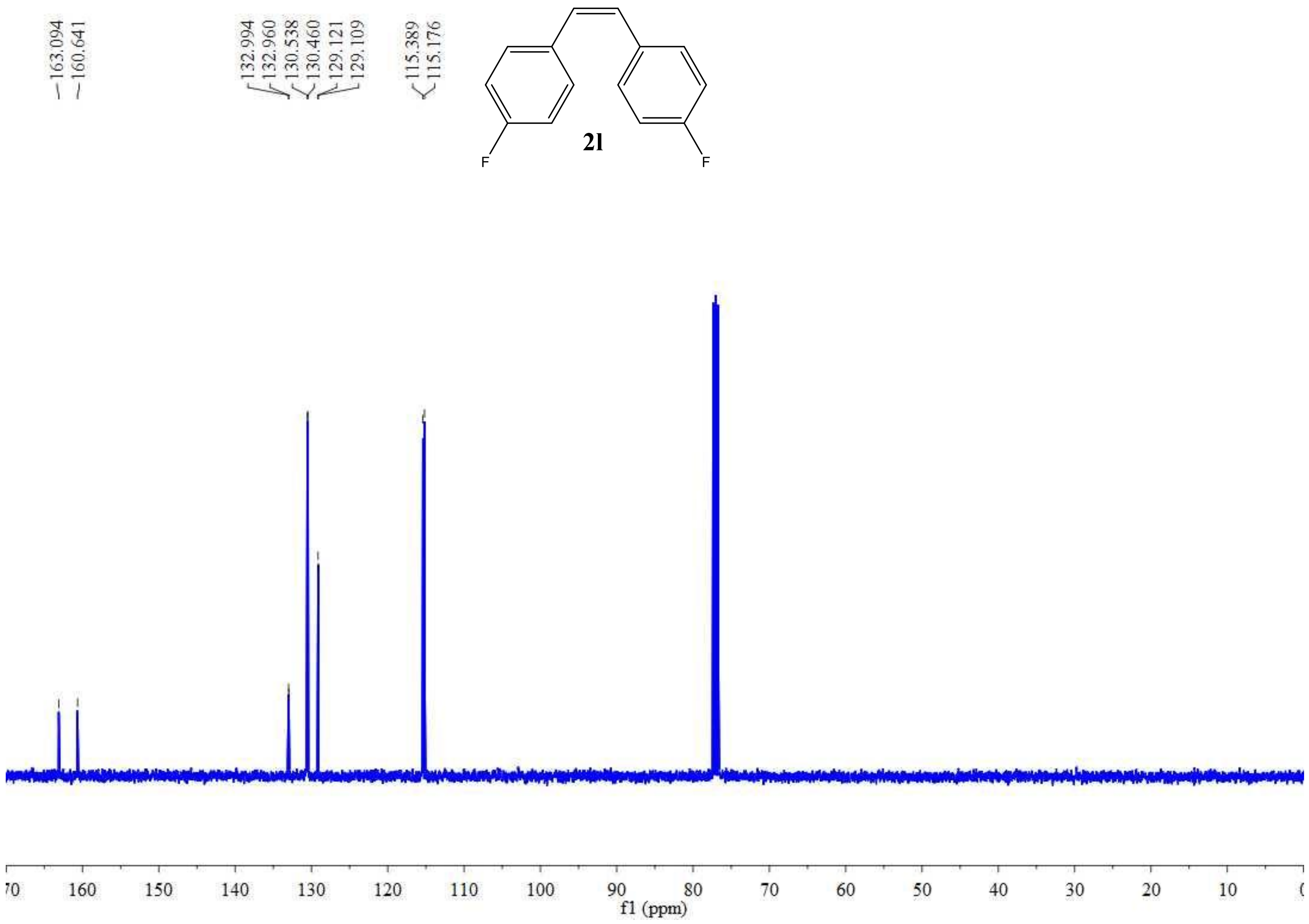
163.094
160.641
132.994
132.960
130.538
130.460
129.121
129.109
115.389
115.176
F
2l
F
70
160
150
140
130
120
110
100
90
80
70
60
50
40
30
20
10
f1 (ppm)

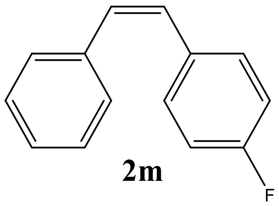
F

2m

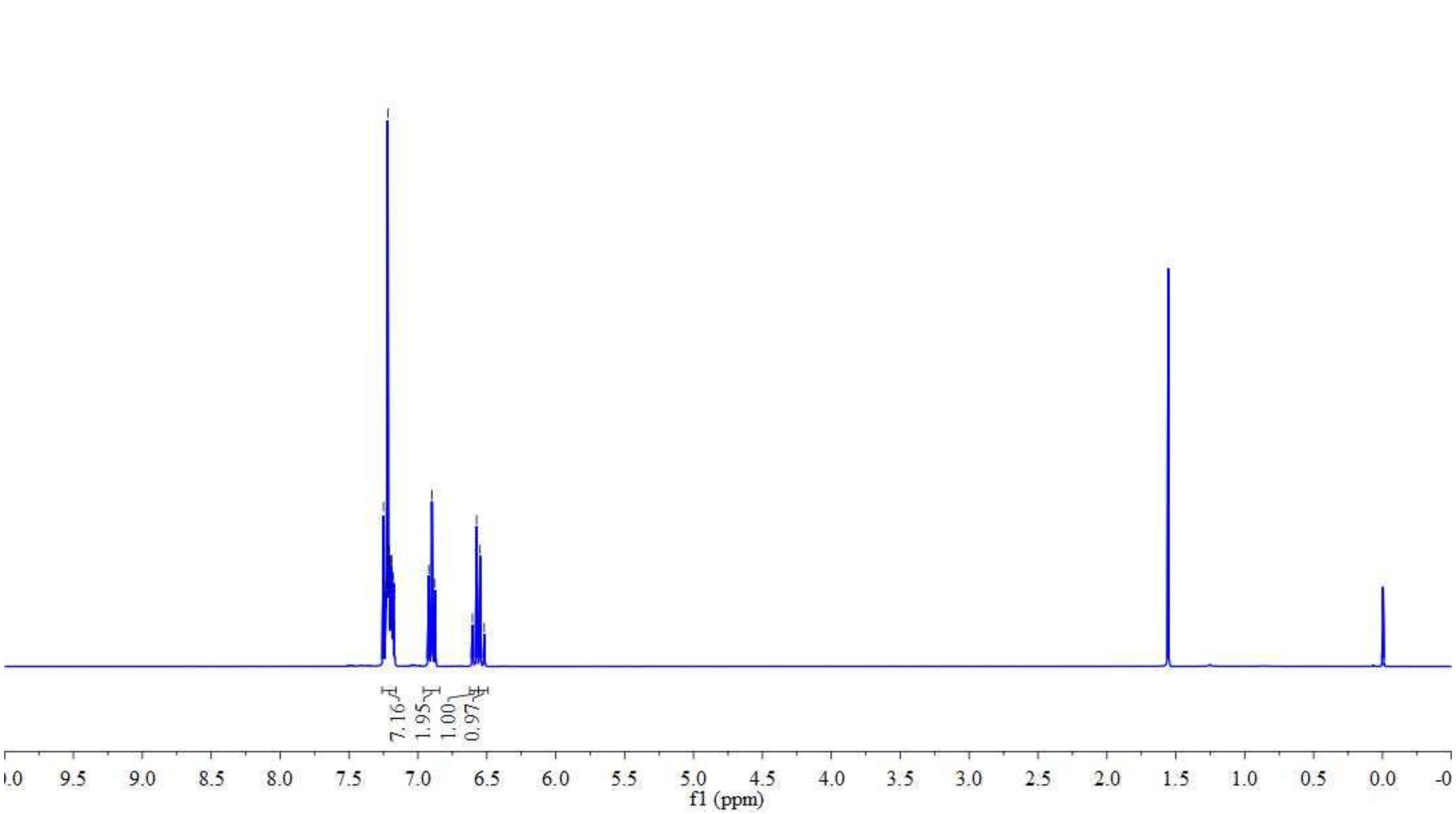
7.250
7.232
7.230
7.226
7.219
7.212
7.209
7.204
7.198
7.191
7.182
7.177
6.921
6.899
6.877
6.605
6.574
6.549
6.519
7.16
1.95
1.00
0.97
9.5
9.0
8.5
8.0
7.5
7.0
6.5
6.0
5.5
5.0
4.5
4.0
3.5
3.0
2.5
2.0
1.5
1.0
0.5
0.0
f1 (ppm)

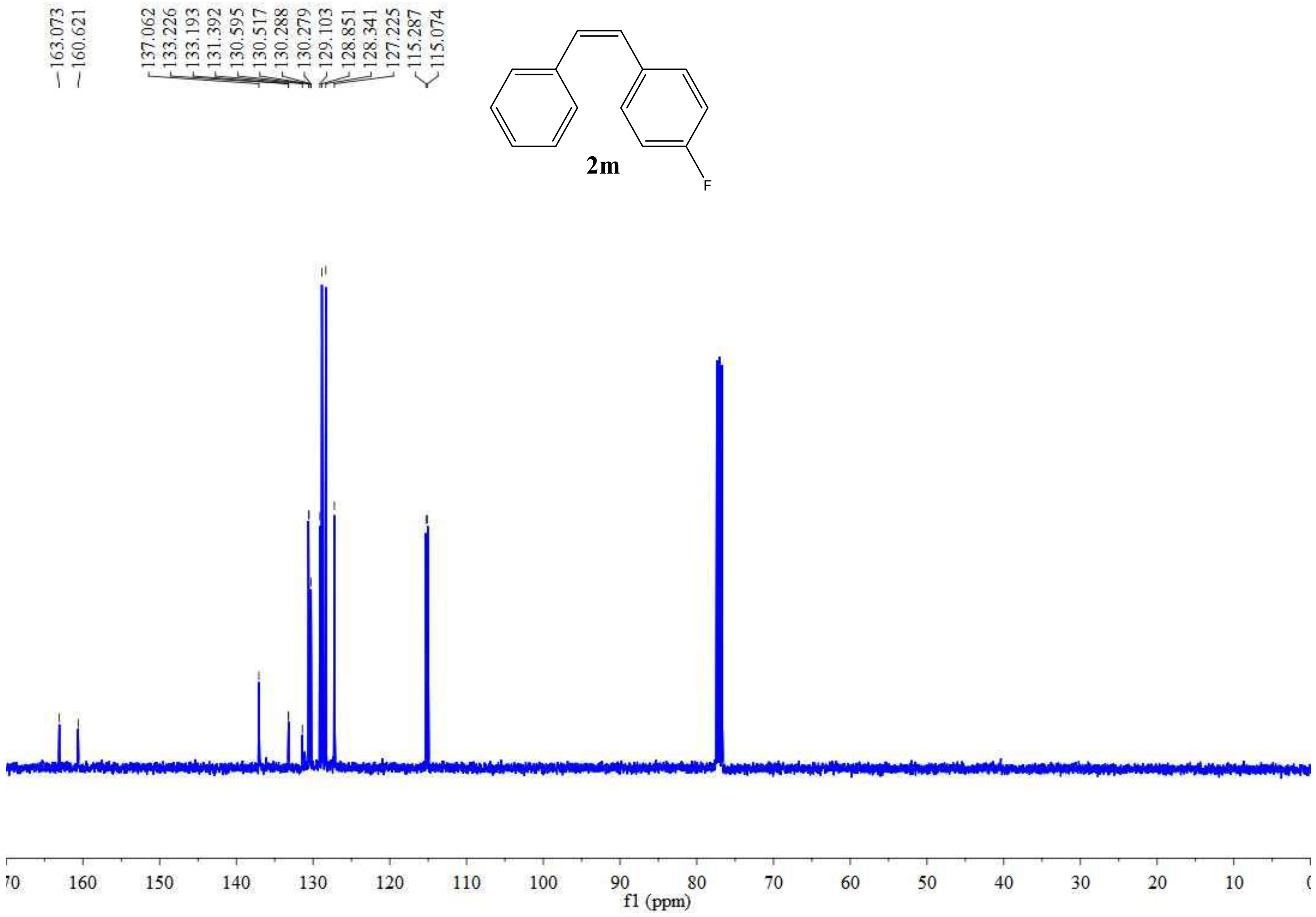
163.073
160.621
137.062
133.226
133.193
131.392
130.595
130.517
130.288
130.279
129.103
128.851
128.341
127.225
115.287
115.074
2m
F
70
160
150
140
130
120
110
100
90
80
70
60
50
40
30
20
10
f1 (ppm)

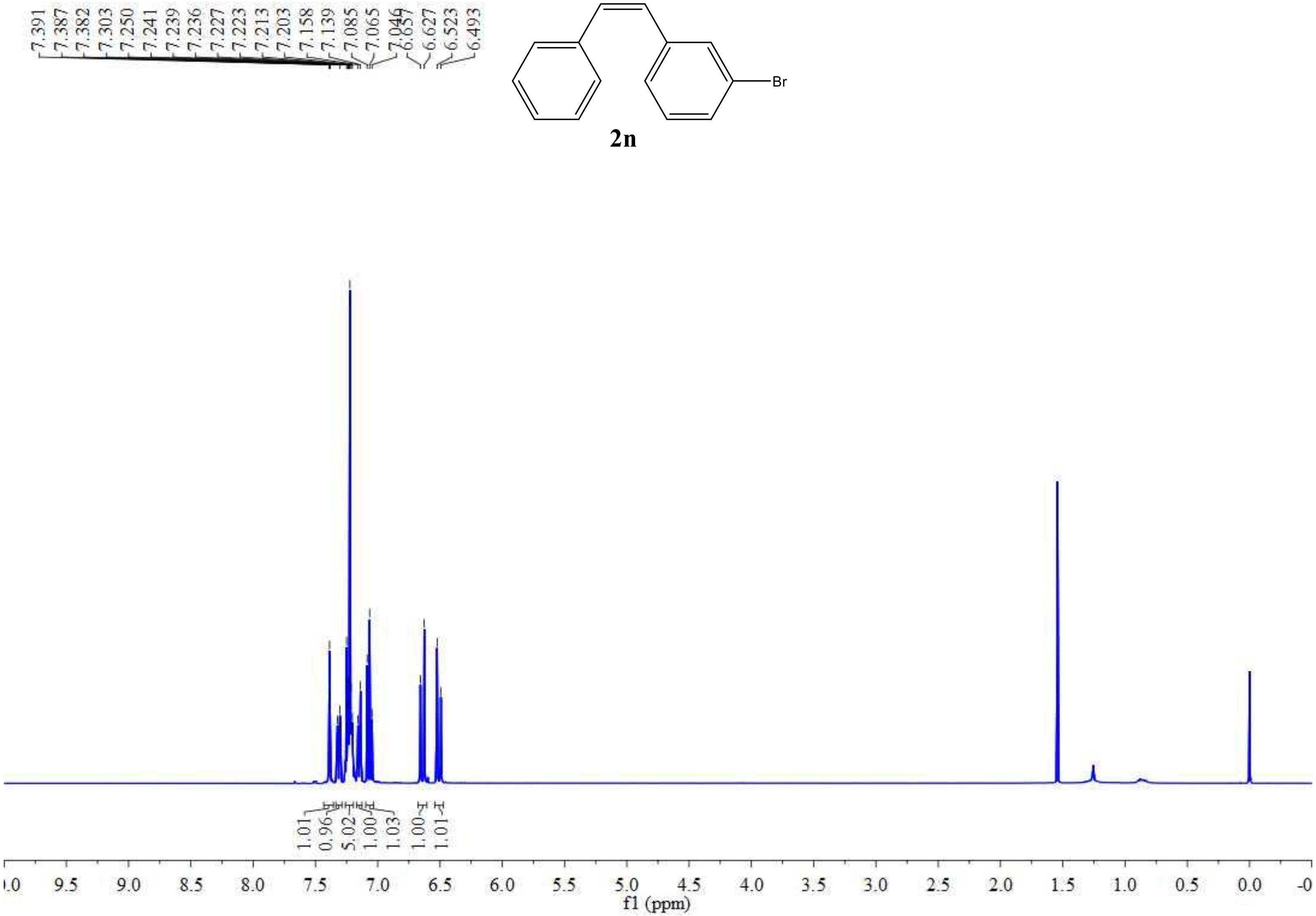
7.391
7.387
7.382
7.303
7.250
7.241
7.239
7.236
7.227
7.223
7.213
7.203
7.158
7.139
7.085
7.065
7.046
6.657
6.627
6.523
6.493
Br
1.01
0.96
5.02
1.00
1.03
1.00
1.01
.0 9.5 9.0 8.5 8.0 7.5 7.0 6.5 6.0 5.5 5.0 4.5 4.0 3.5 3.0 2.5 2.0 1.5 1.0 0.5 0.0 -0
f1 (ppm)

2n

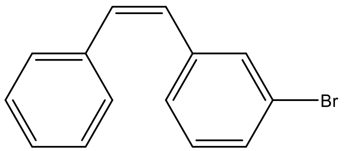
Br

2n

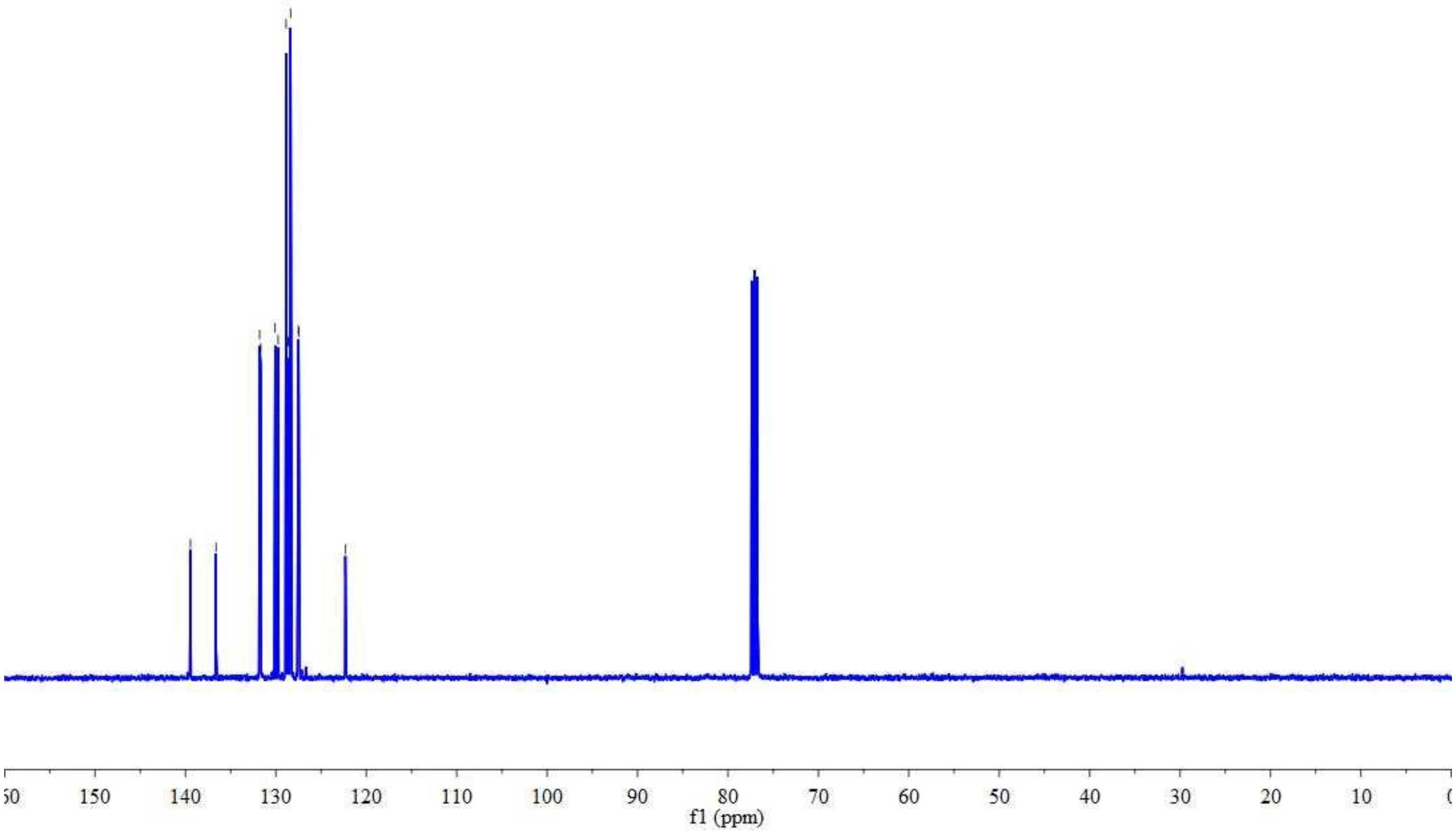
139.449
136.613
131.791
131.657
130.076
129.745
128.862
128.639
128.365
127.506
127.469
122.310
150
140
130
120
110
100
90
80
70
60
50
40
30
20
10
f1 (ppm)

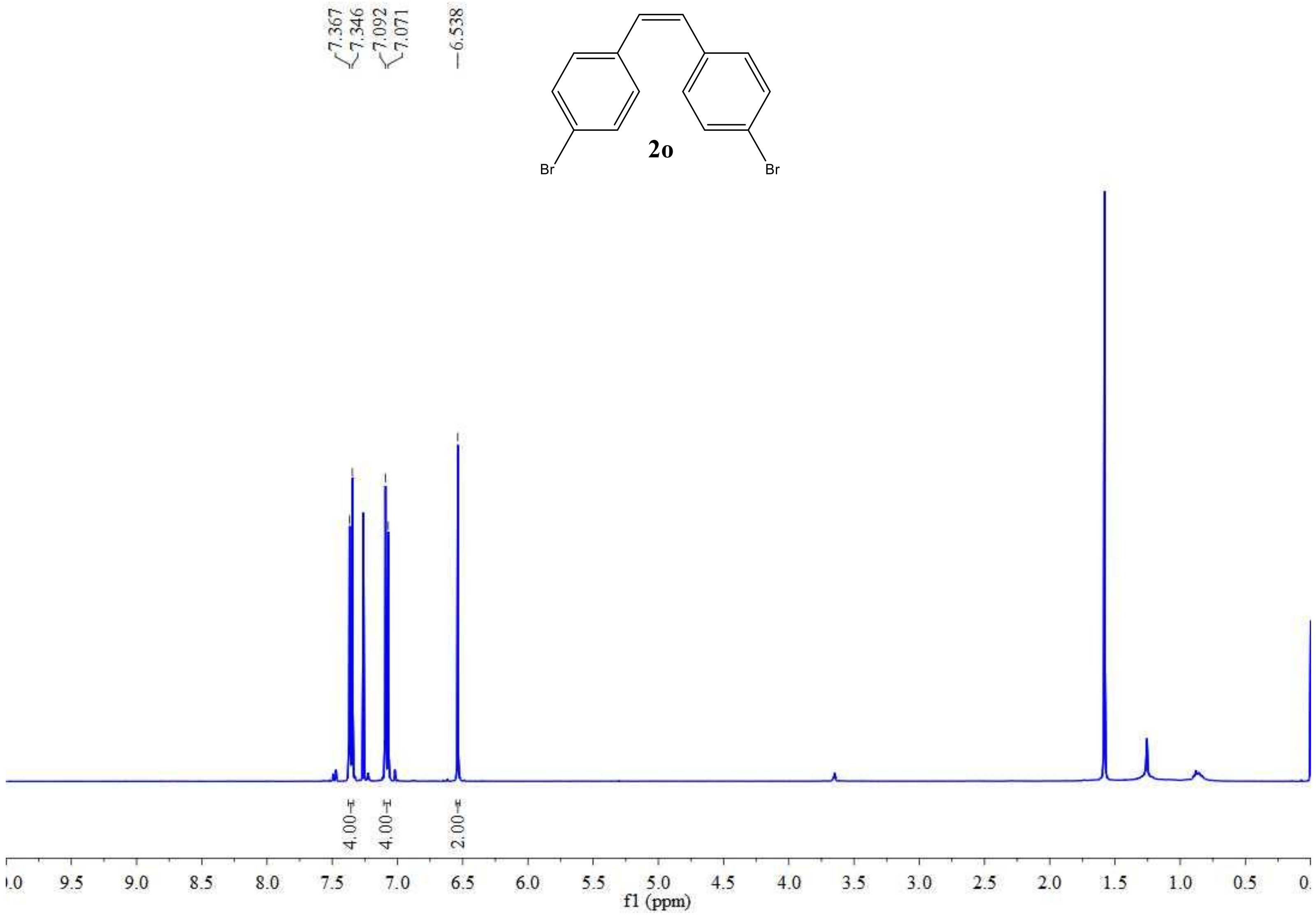
7.367
7.346
7.092
7.071
6.538
2o
Br
Br
4.00
4.00
2.00
10.0 9.5 9.0 8.5 8.0 7.5 7.0 6.5 6.0 5.5 5.0 4.5 4.0 3.5 3.0 2.5 2.0 1.5 1.0 0.5 0.
f1 (ppm)

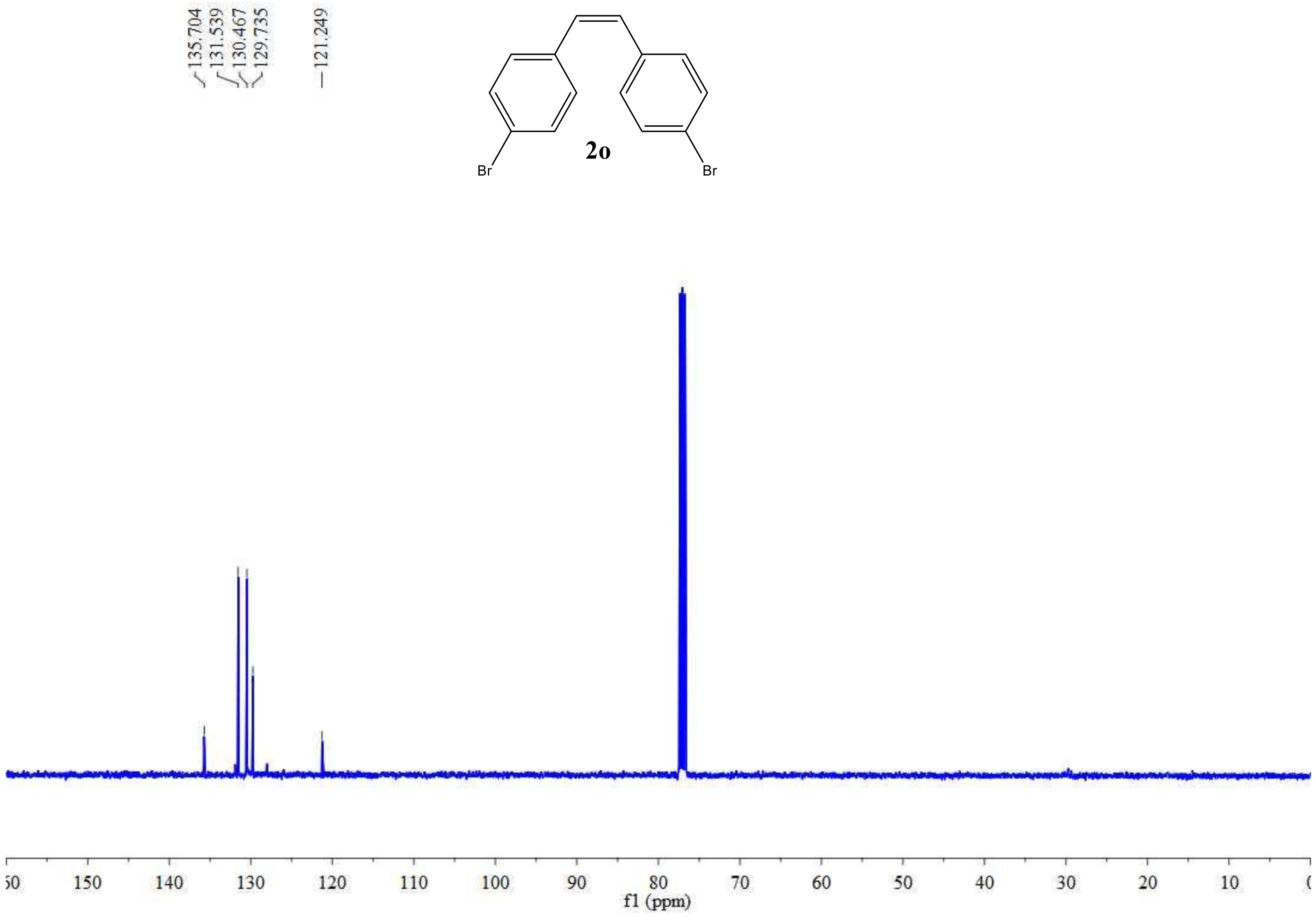
135.704
131.539
130.467
129.735
121.249
Br
Br
2o
150
140
130
120
110
100
90
80
70
60
50
40
30
20
10
f1 (ppm)

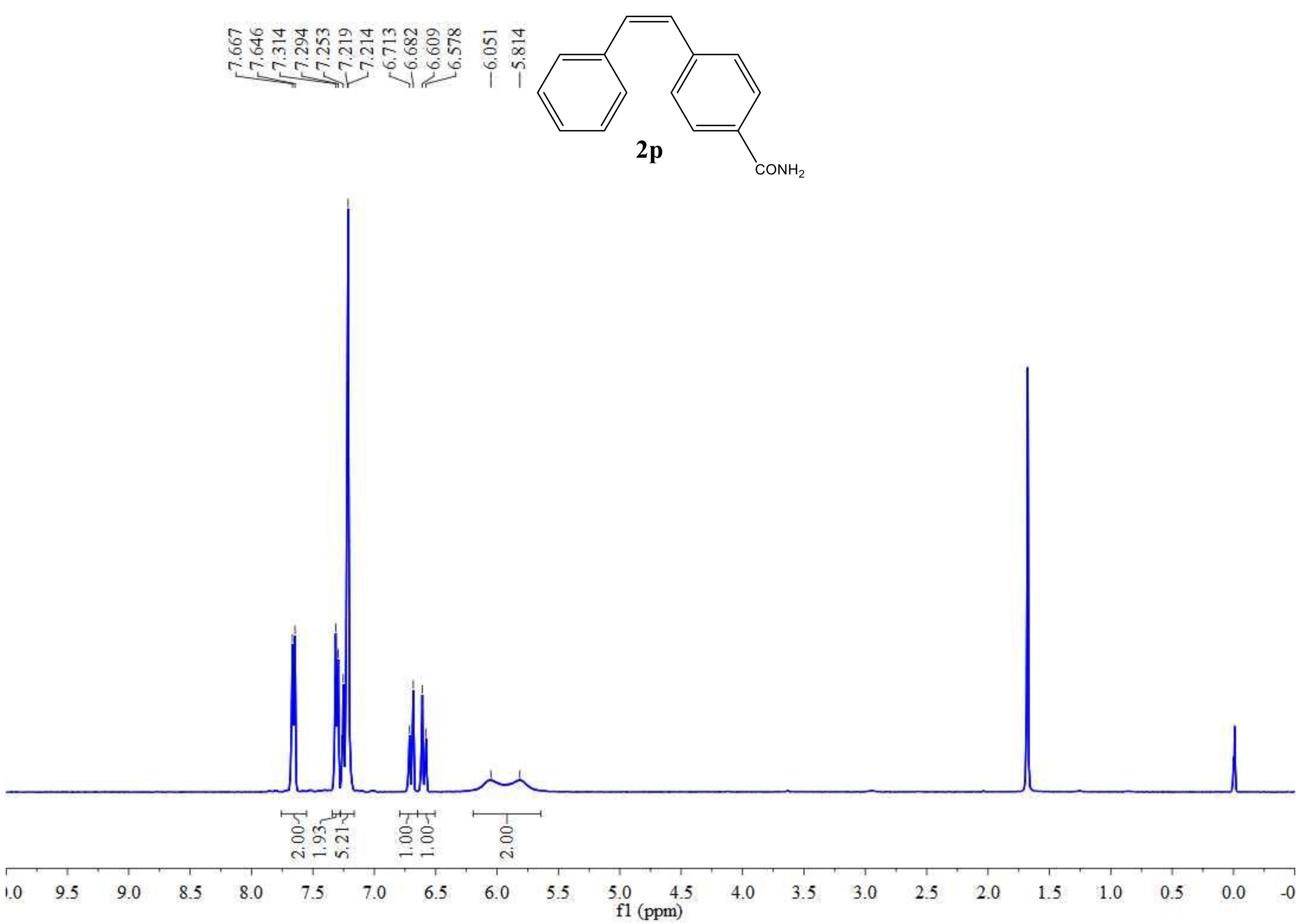

7.667
7.646
7.314
7.294
7.253
7.219
7.214
6.713
6.682
6.609
6.578
6.051
5.814
CONH$_2$
2.00
1.93
5.21
1.00
1.00
2.00
f1 (ppm)

2p

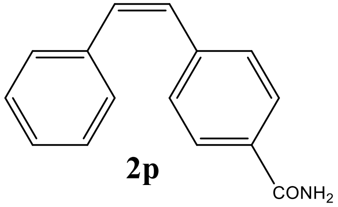

CONH$_2$

2p

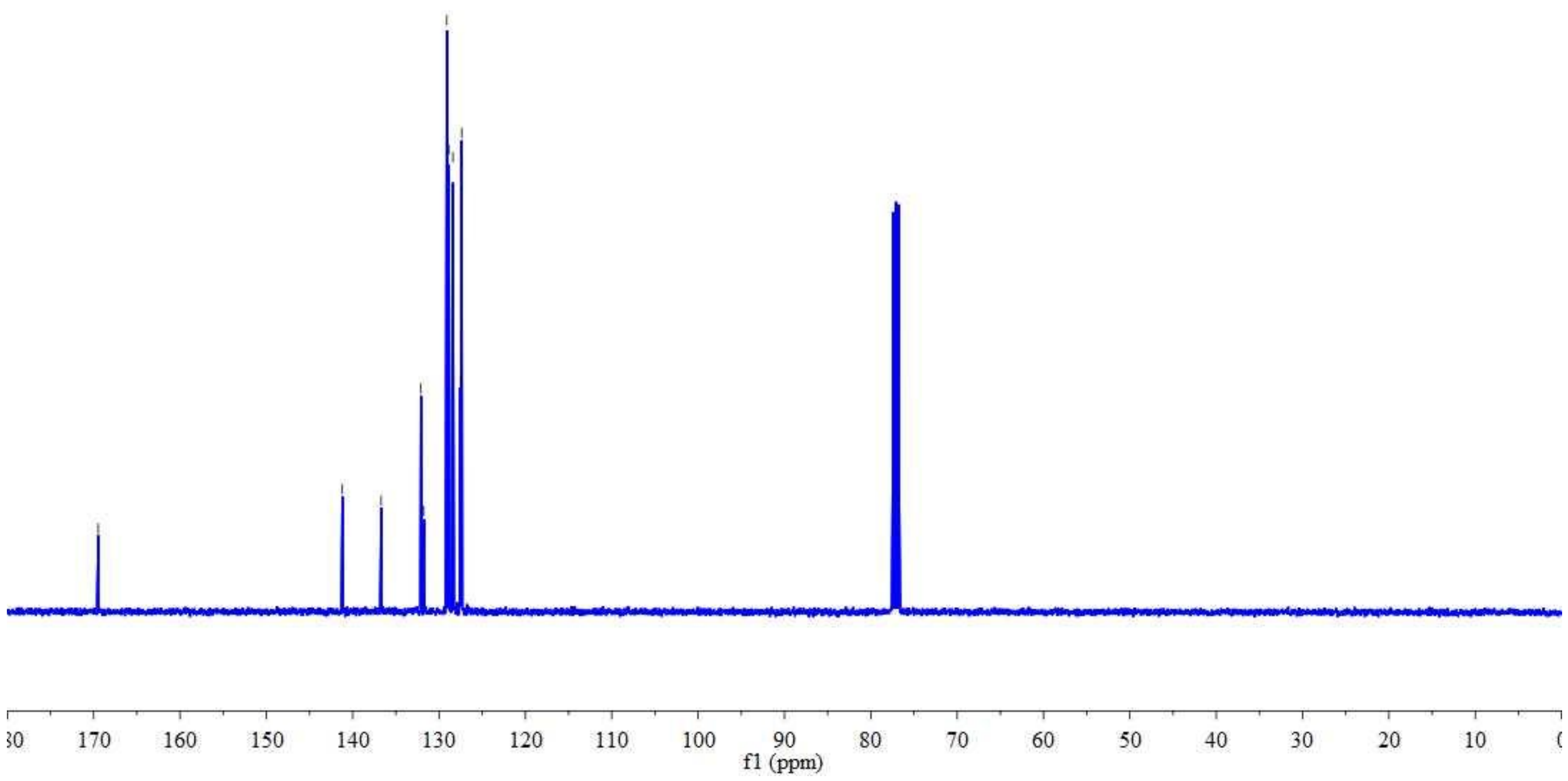

169.472
141.200
136.704
132.101
131.760
129.103
128.875
128.391
127.525
127.376
30
170
160
150
140
130
120
110
100
90
80
70
60
50
40
30
20
10
f1 (ppm)

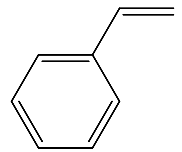

4a

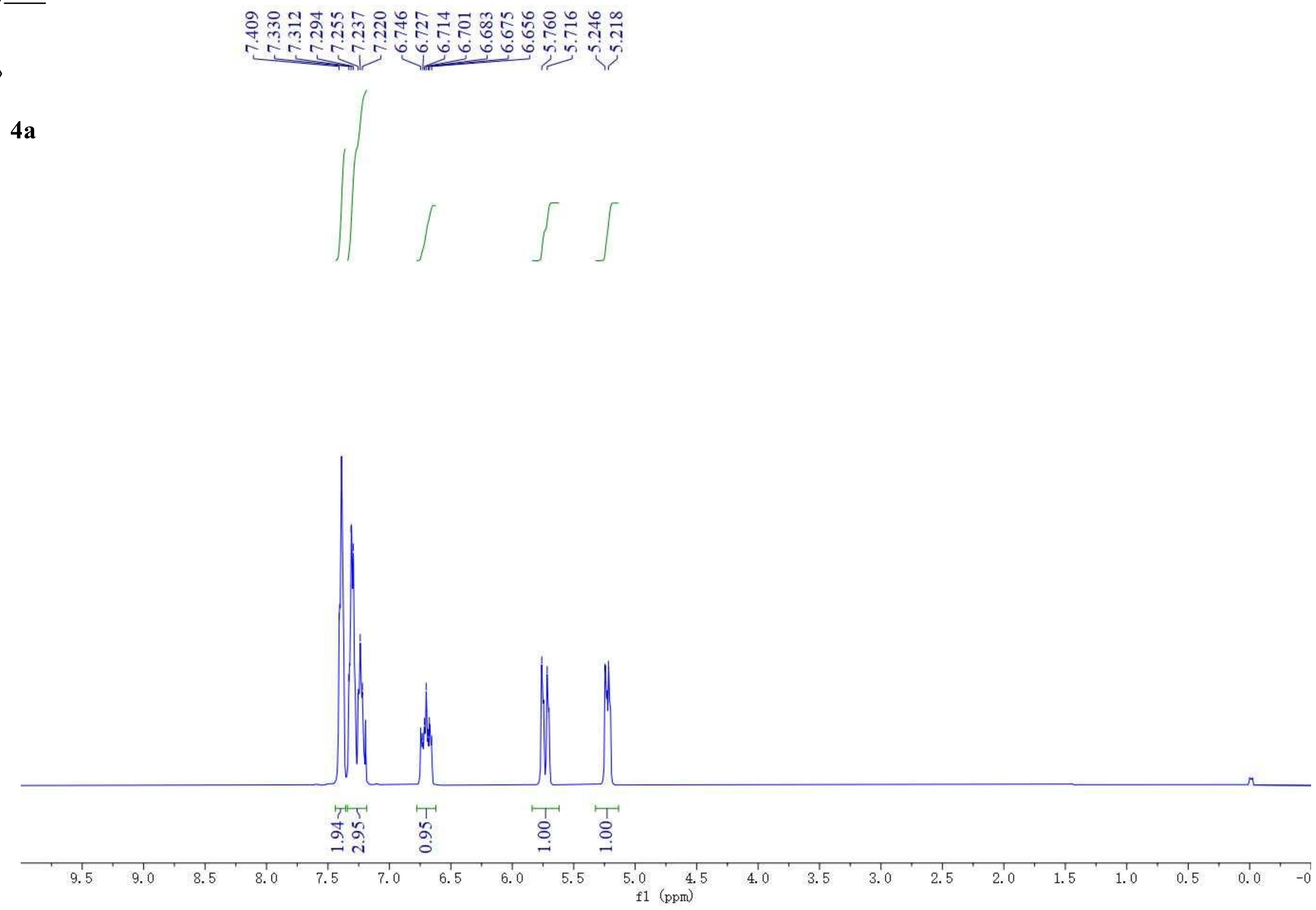

7.409
7.330
7.312
7.294
7.255
7.237
7.220
6.746
6.727
6.714
6.701
6.683
6.675
6.656
5.760
5.716
5.246
5.218
1.94
2.95
0.95
1.00
1.00
9.5
9.0
8.5
8.0
7.5
7.0
6.5
6.0
5.5
5.0
4.5
4.0
3.5
3.0
2.5
2.0
1.5
1.0
0.5
0.0
-0
f1 (ppm)

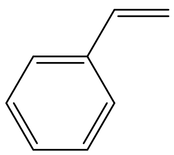

4a

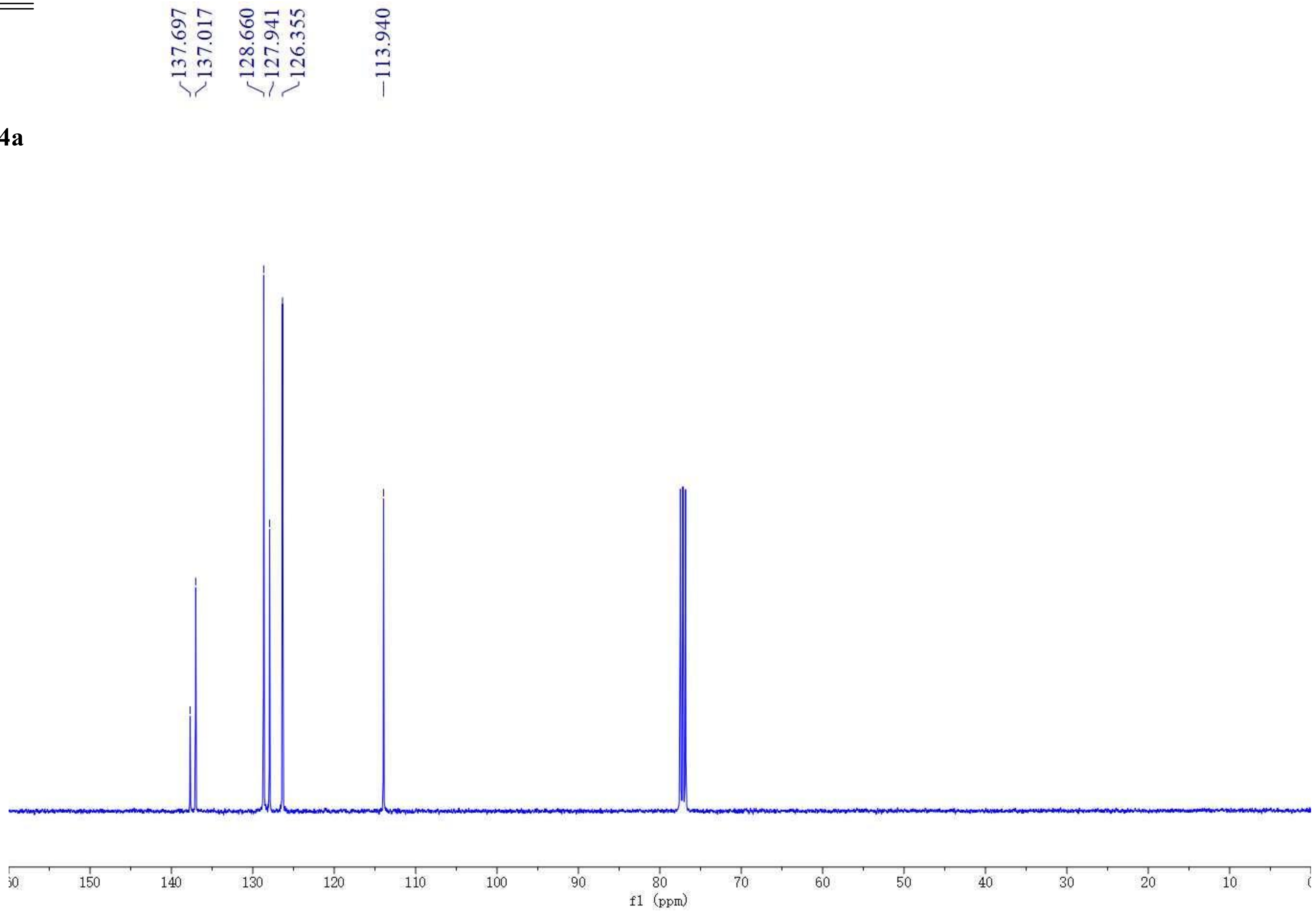
137.697
137.017
128.660
127.941
126.355
113.940
160
150
140
130
120
110
100
90
80
70
60
50
40
30
20
10
0
f1 (ppm)

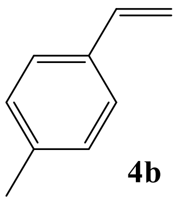

4b

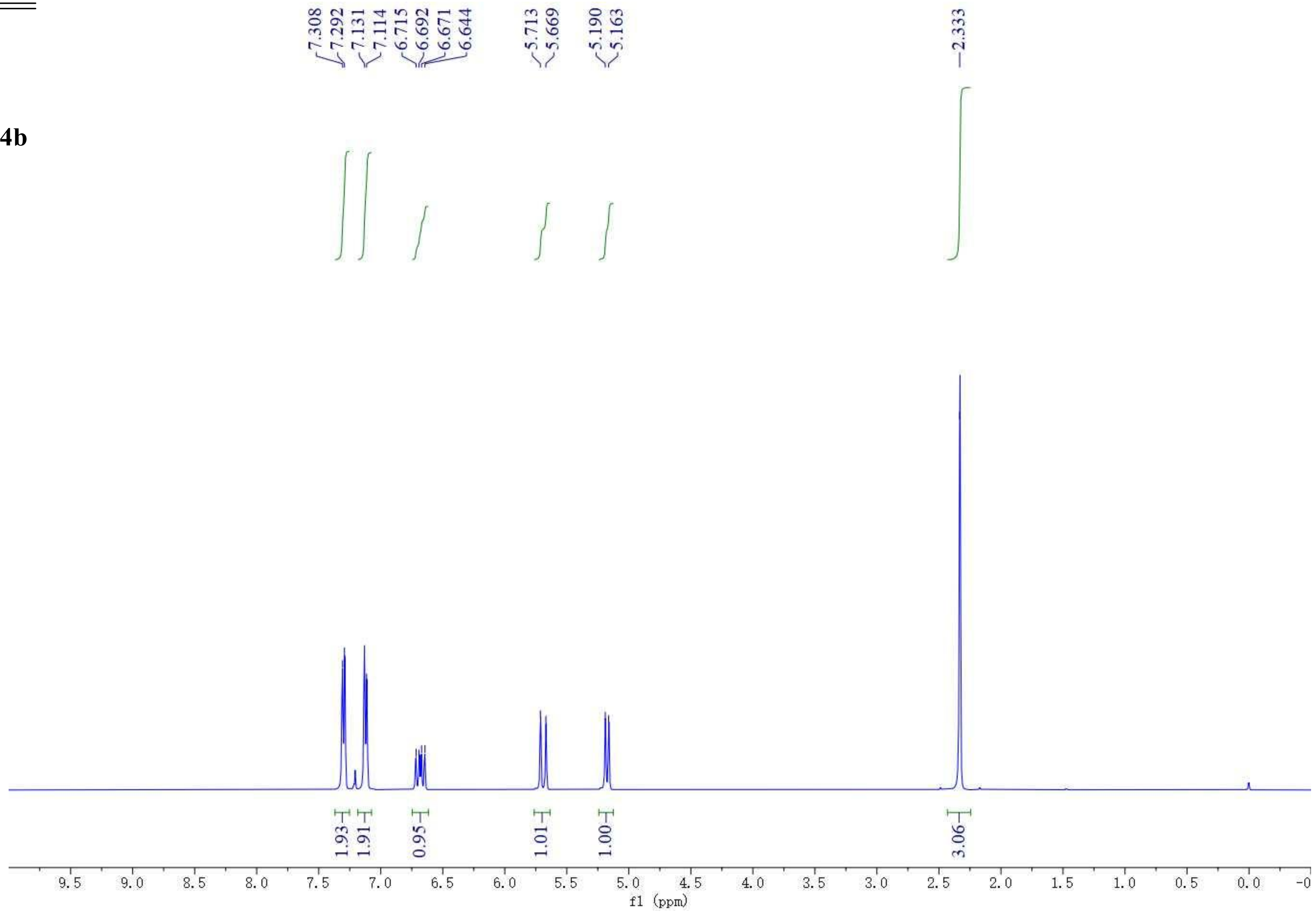

7.308
7.292
7.131
7.114
6.715
6.692
6.671
6.644
5.713
5.669
5.190
5.163
2.333
1.93
1.91
0.95
1.01
1.00
3.06
9.5 9.0 8.5 8.0 7.5 7.0 6.5 6.0 5.5 5.0 4.5 4.0 3.5 3.0 2.5 2.0 1.5 1.0 0.5 0.0 -0
f1 (ppm)

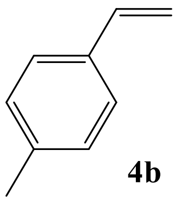

4b

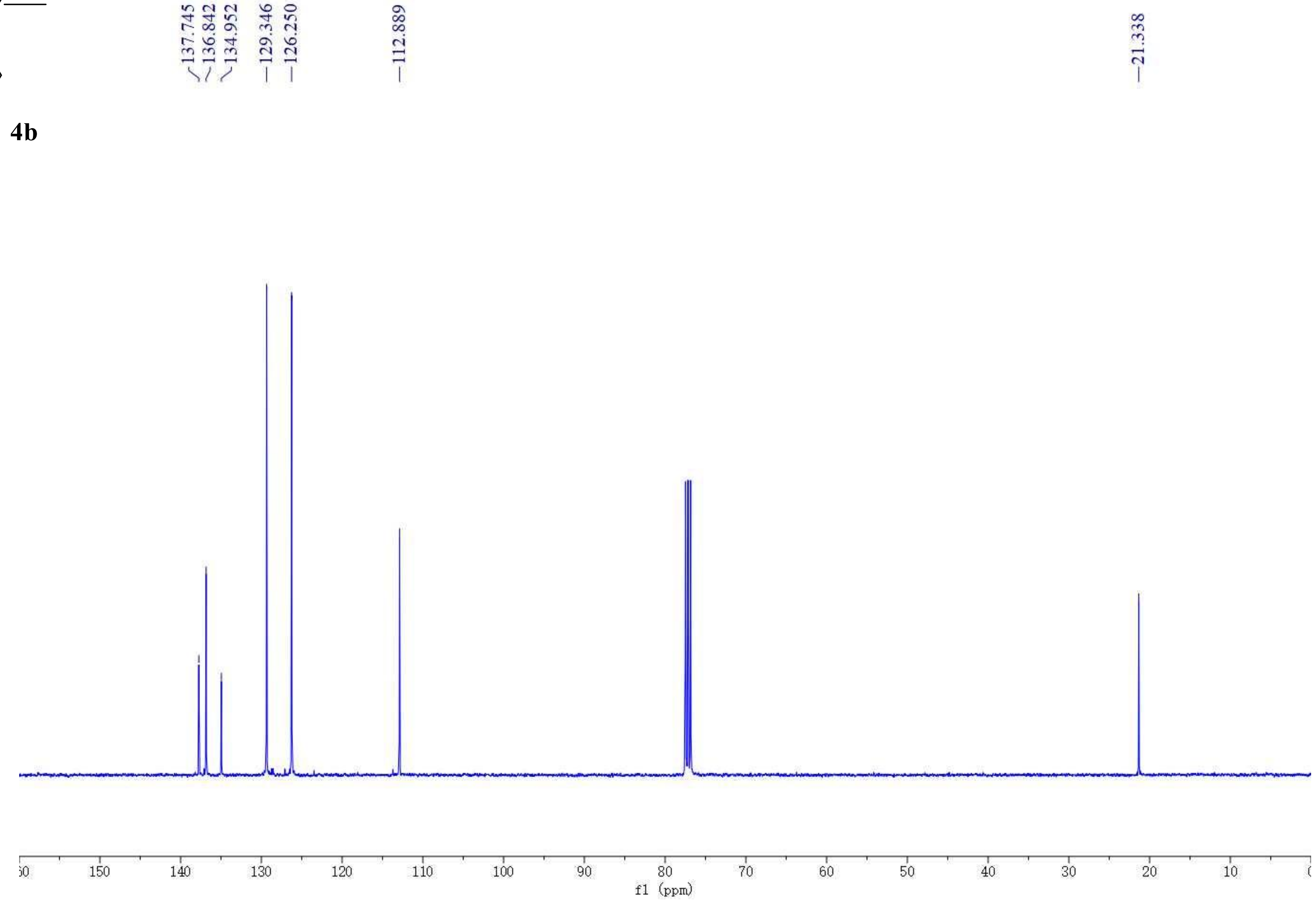
137.745
136.842
134.952
129.346
126.250
112.889
21.338
150
140
130
120
110
100
90
80
70
60
50
40
30
20
10
f1 (ppm)

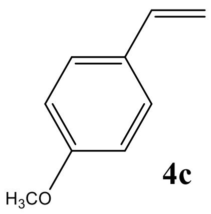
H3CO

4c

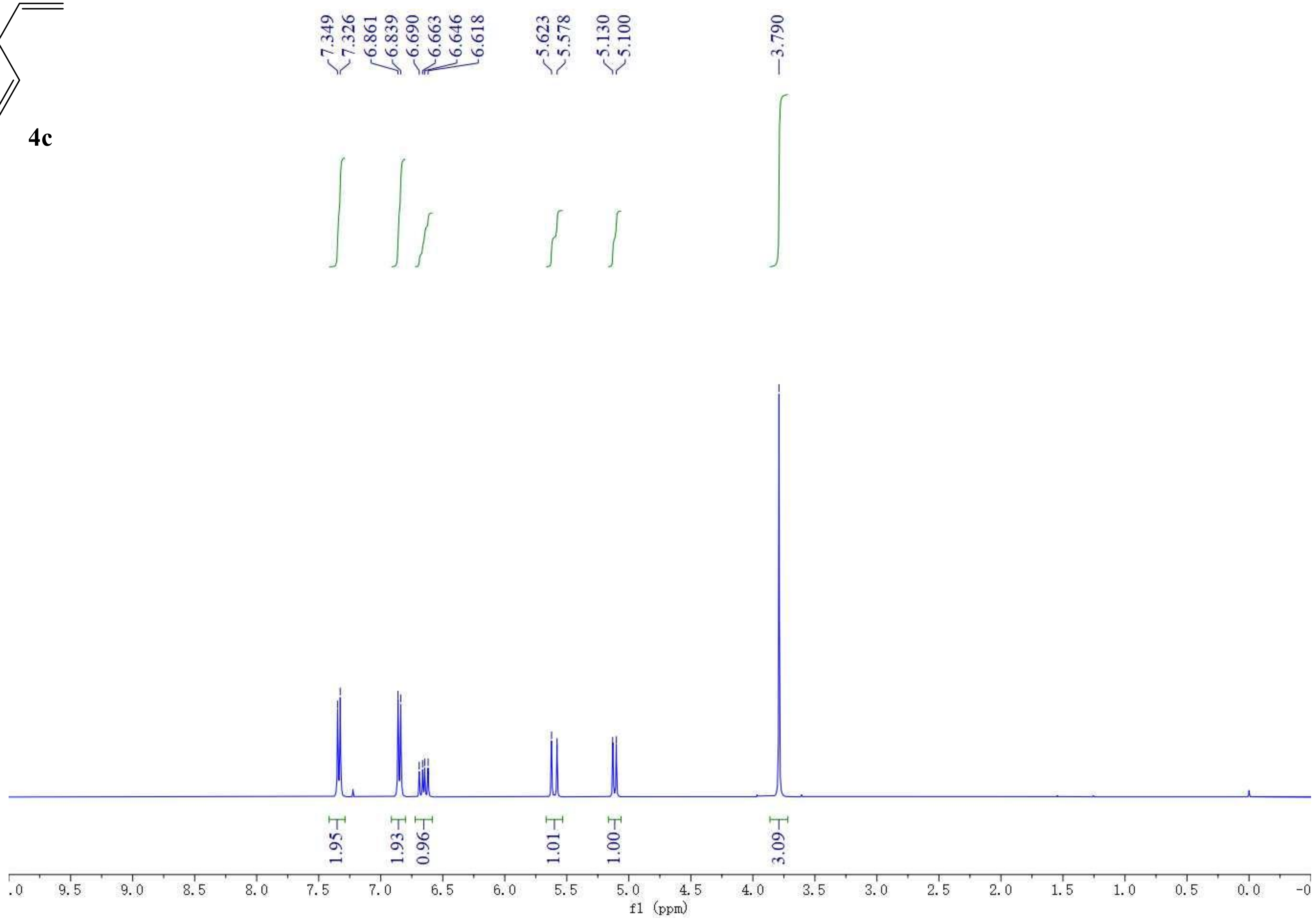
7.349
7.326
6.861
6.839
6.690
6.663
6.646
6.618
5.623
5.578
5.130
5.100
3.790
1.95
1.93
0.96
1.01
1.00
3.09
.0
9.5
9.0
8.5
8.0
7.5
7.0
6.5
6.0
5.5
5.0
4.5
4.0
3.5
3.0
2.5
2.0
1.5
1.0
0.5
0.0
-0
f1 (ppm)

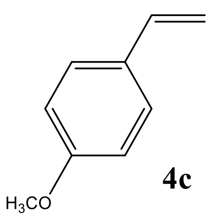
H3CO

4c

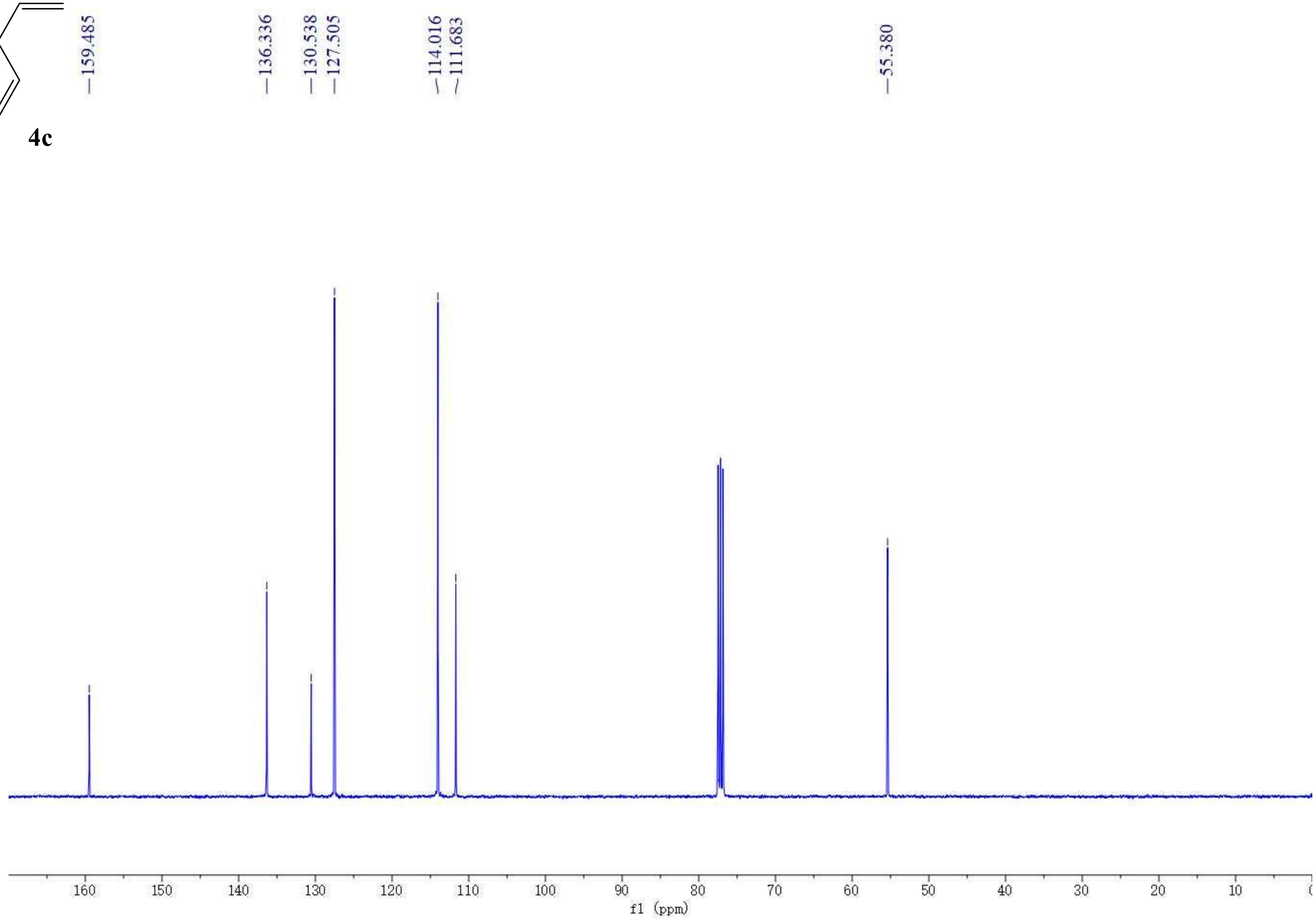
159.485
136.336
130.538
127.505
114.016
111.683
55.380
160
150
140
130
120
110
100
90
80
70
60
50
40
30
20
10
f1 (ppm)

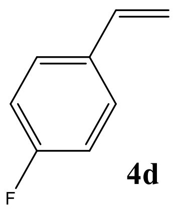
F

4d

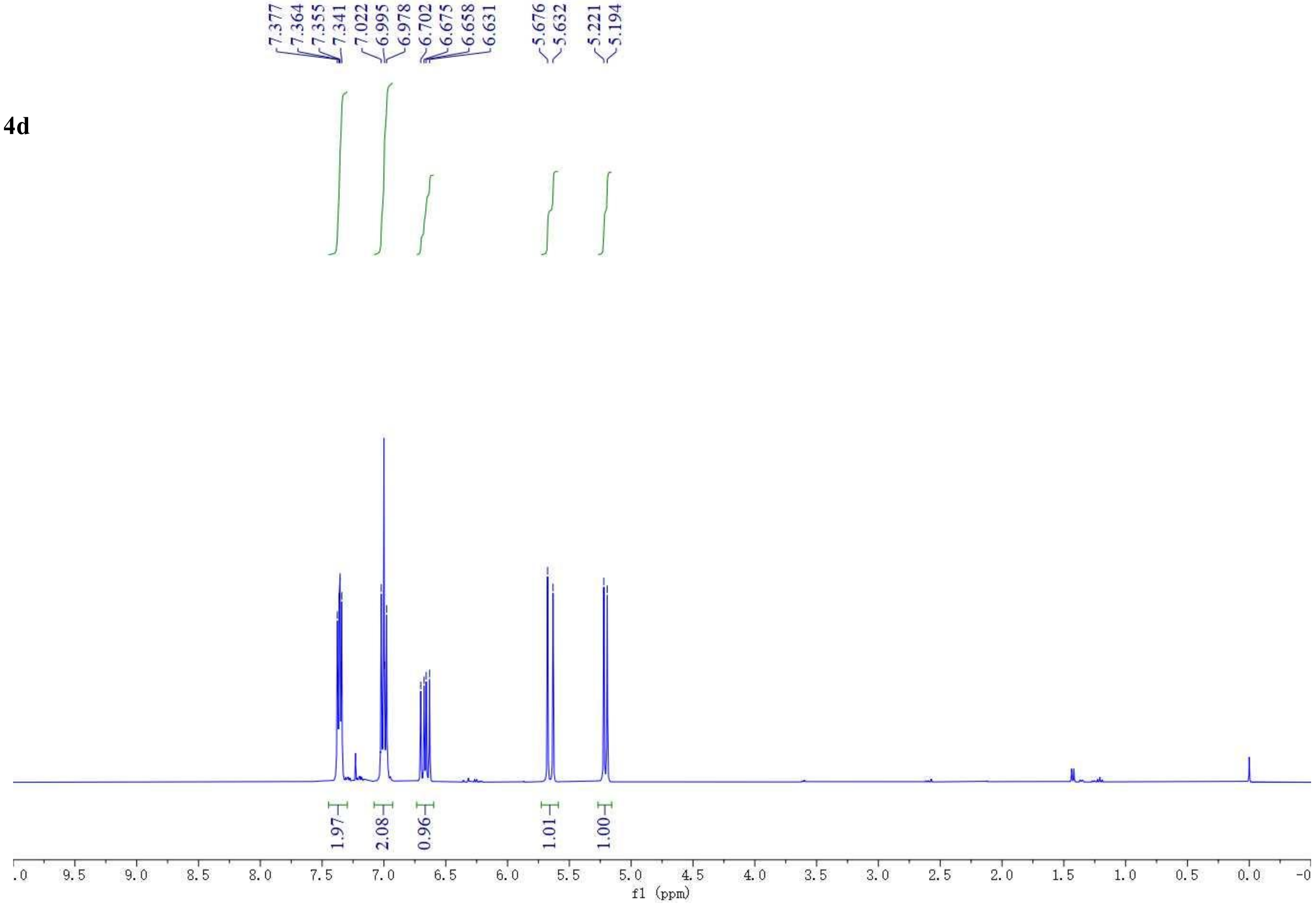
7.377
7.364
7.355
7.341
7.022
6.995
6.978
6.702
6.675
6.658
6.631
5.676
5.632
5.221
5.194
1.97
2.08
0.96
1.01
1.00
.0
9.5
9.0
8.5
8.0
7.5
7.0
6.5
6.0
5.5
5.0
4.5
4.0
3.5
3.0
2.5
2.0
1.5
1.0
0.5
0.0
-0
f1 (ppm)

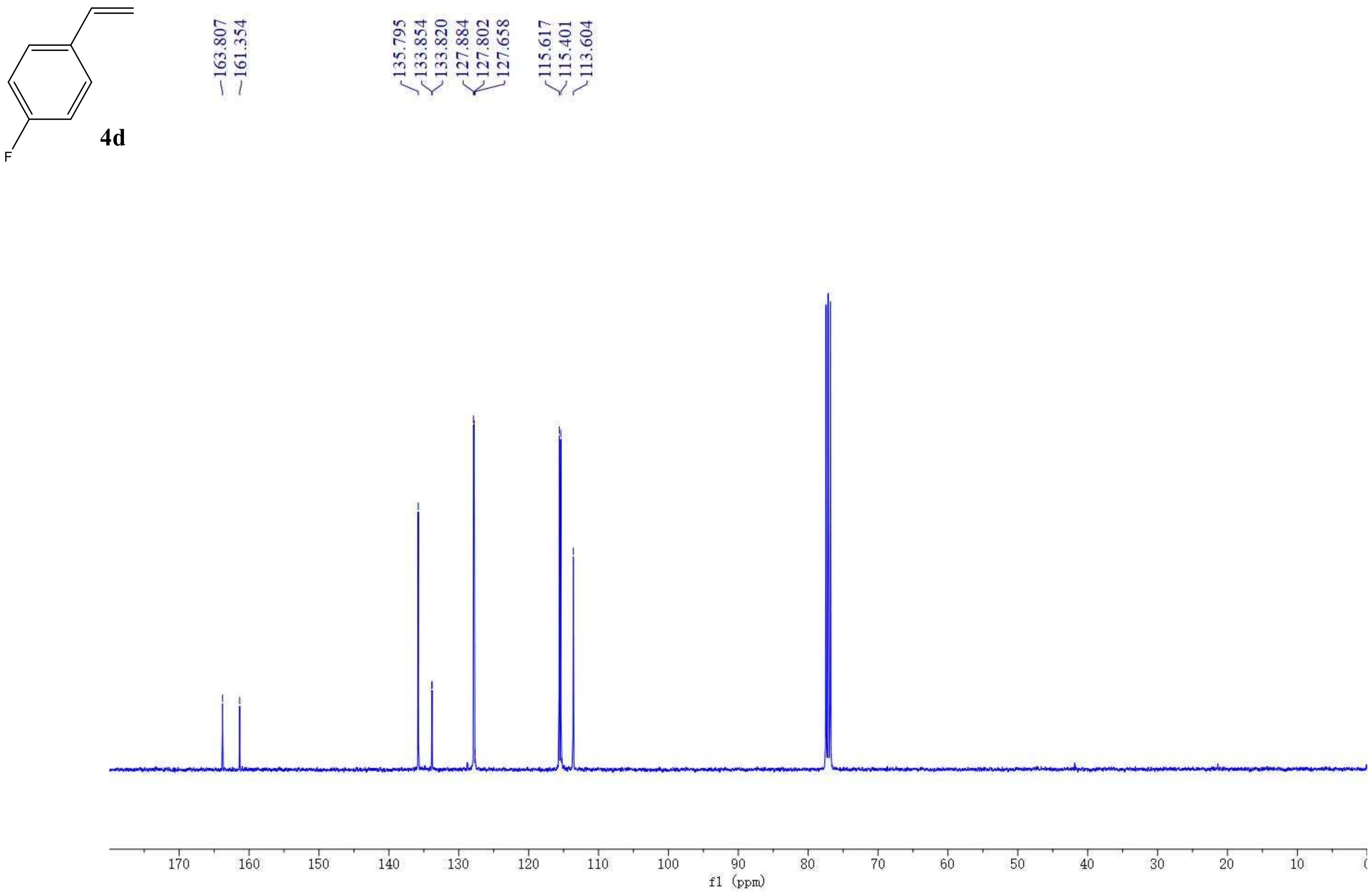
F
4d
163.807
161.354
135.795
133.854
133.820
127.884
127.802
127.658
115.617
115.401
113.604
170
160
150
140
130
120
110
100
90
80
70
60
50
40
30
20
10
f1 (ppm)

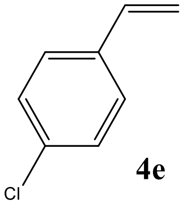
Cl

4e

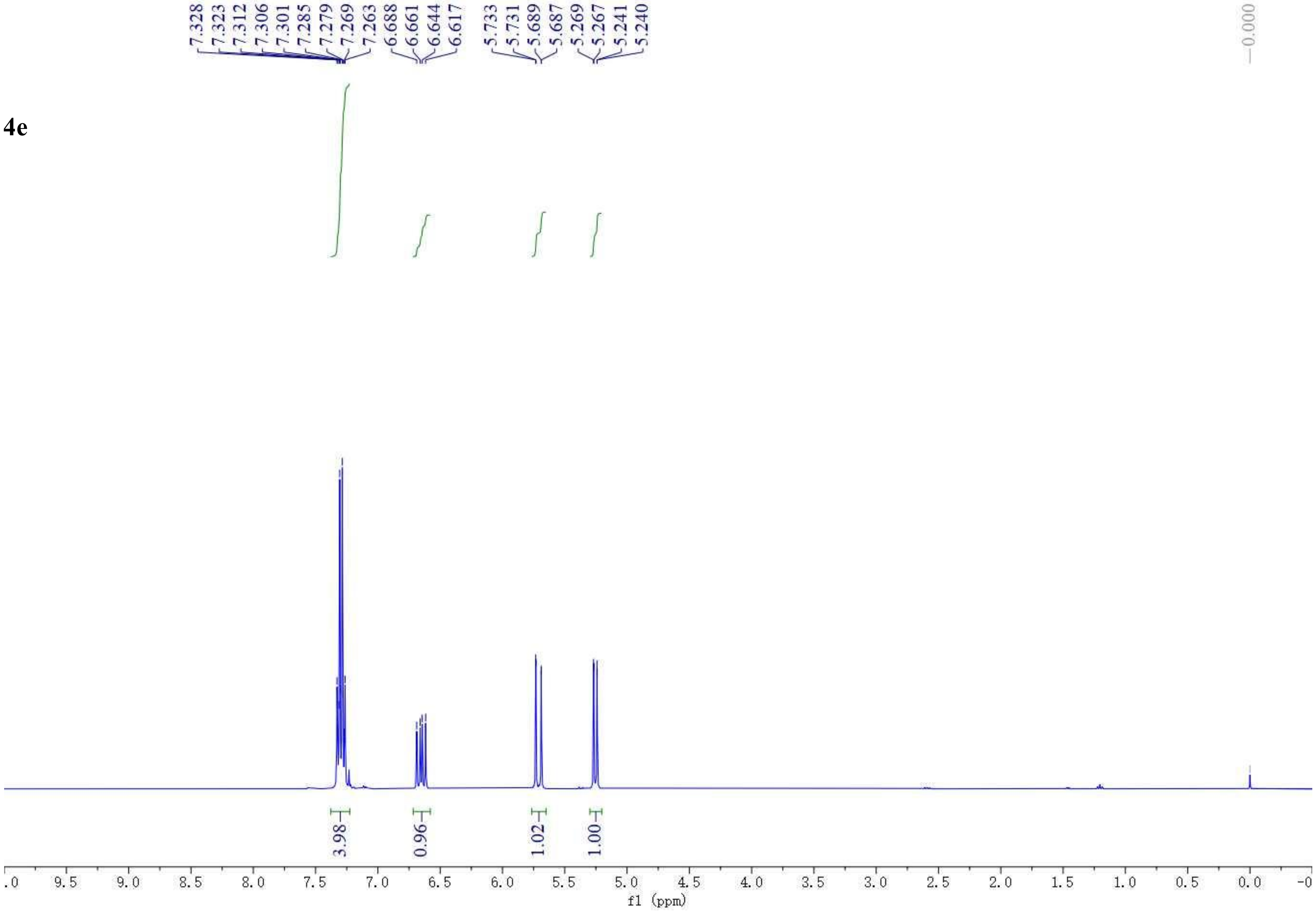
7.328
7.323
7.312
7.306
7.301
7.285
7.279
7.269
7.263
6.688
6.661
6.644
6.617
5.733
5.731
5.689
5.687
5.269
5.267
5.241
5.240
0.000
3.98
0.96
1.02
1.00
f1 (ppm)

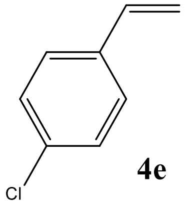

4e

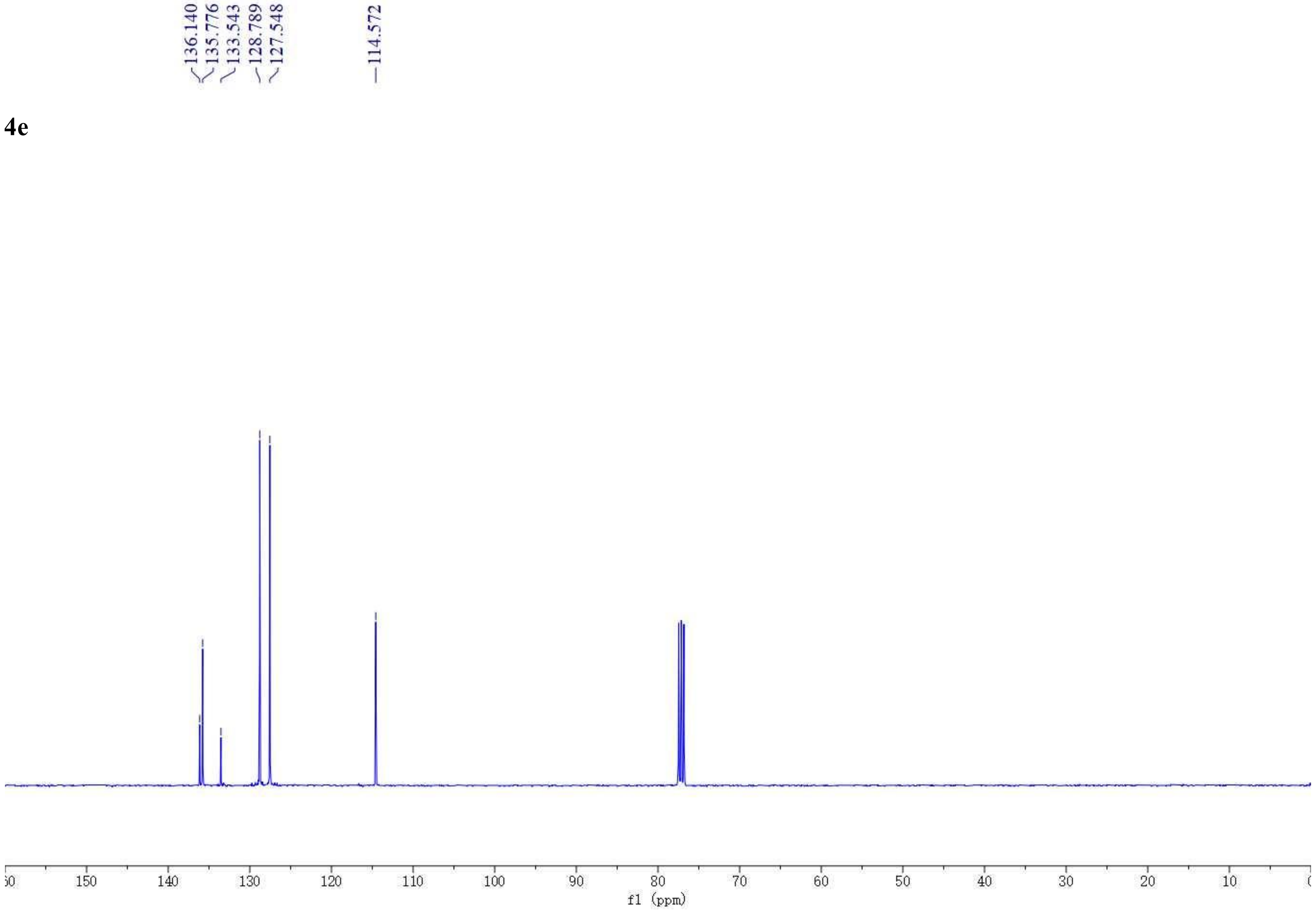
136.140
135.776
133.543
128.789
127.548
114.572
150
140
130
120
110
100
90
80
70
60
50
40
30
20
10
f1 (ppm)

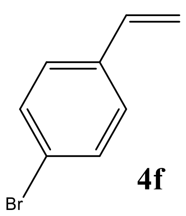

4f

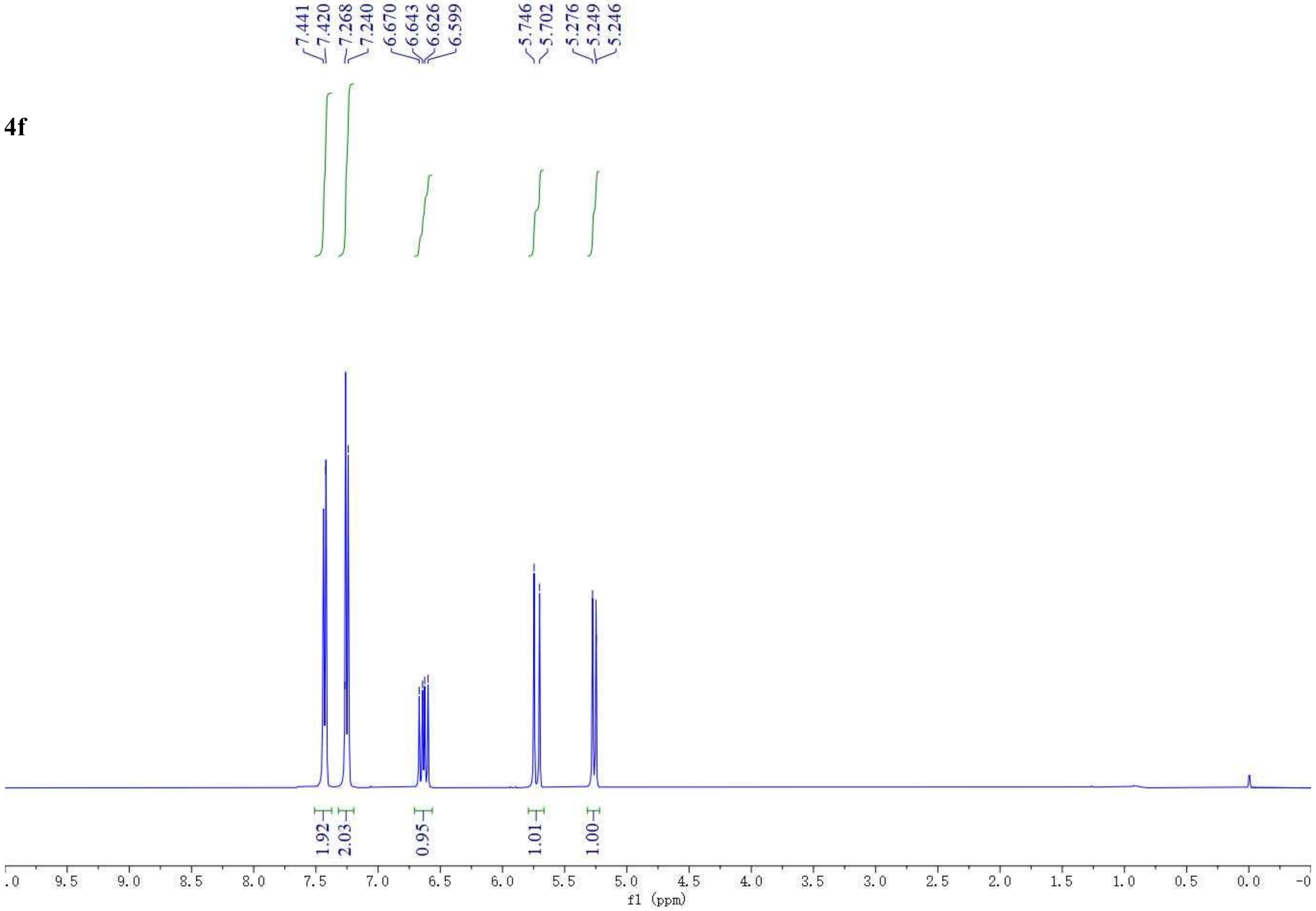

7.441
7.420
7.268
7.240
6.670
6.643
6.626
6.599
5.746
5.702
5.276
5.249
5.246
1.92
2.03
0.95
1.01
1.00
.0
9.5
9.0
8.5
8.0
7.5
7.0
6.5
6.0
5.5
5.0
4.5
4.0
3.5
3.0
2.5
2.0
1.5
1.0
0.5
0.0
-0
f1 (ppm)

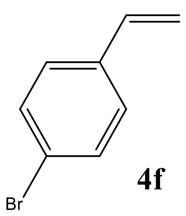
Br

4f

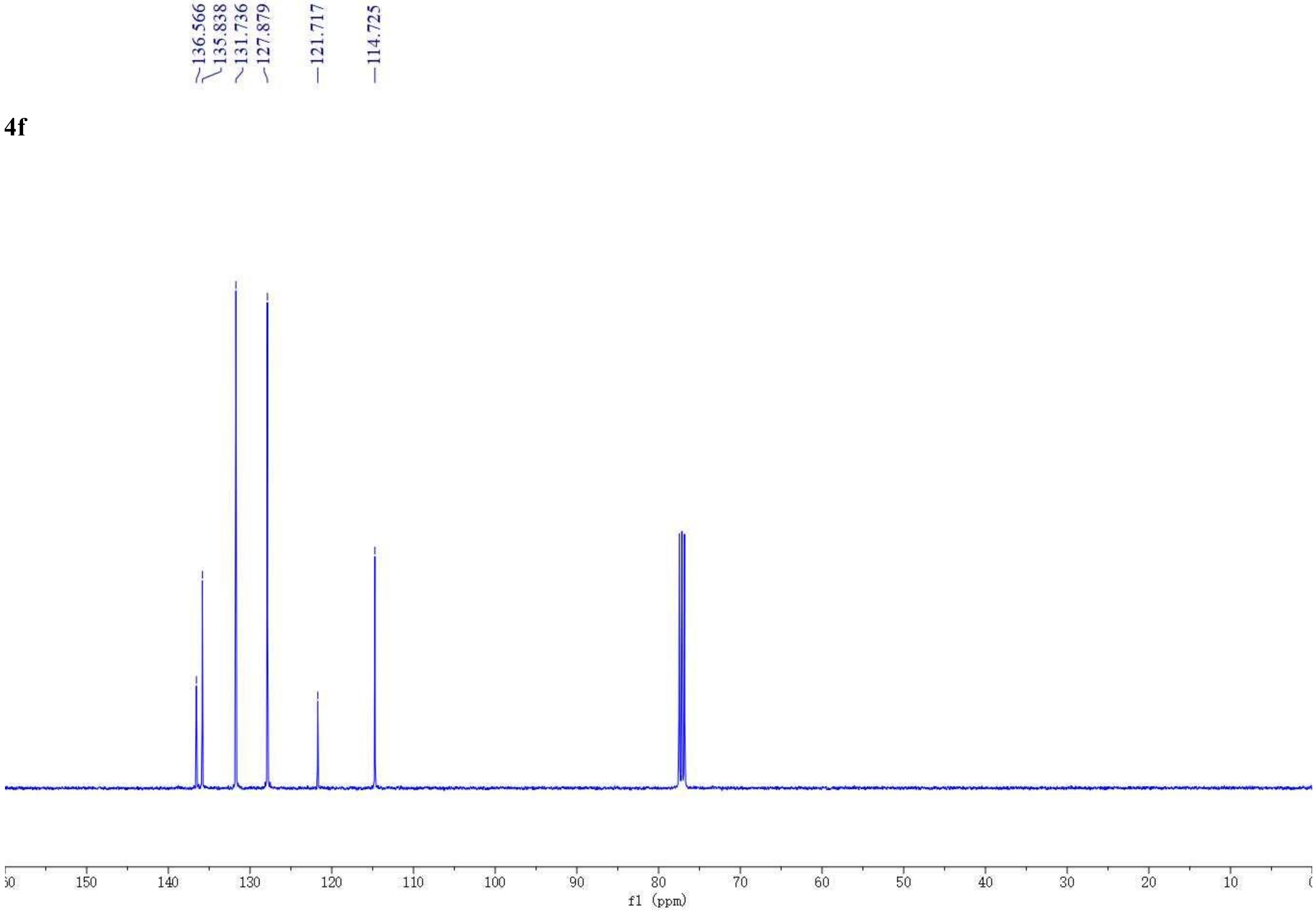
136.566
135.838
131.736
127.879
121.717
114.725
150
140
130
120
110
100
90
80
70
60
50
40
30
20
10
f1 (ppm)
